# Variation block-based genomics method for crop plants

**DOI:** 10.1186/1471-2164-15-477

**Published:** 2014-06-15

**Authors:** Yul Ho Kim, Hyang Mi Park, Tae-Young Hwang, Seuk Ki Lee, Man Soo Choi, Sungwoong Jho, Seungwoo Hwang, Hak-Min Kim, Dongwoo Lee, Byoung-Chul Kim, Chang Pyo Hong, Yun Sung Cho, Hyunmin Kim, Kwang Ho Jeong, Min Jung Seo, Hong Tai Yun, Sun Lim Kim, Young-Up Kwon, Wook Han Kim, Hye Kyung Chun, Sang Jong Lim, Young-Ah Shin, Ik-Young Choi, Young Sun Kim, Ho-Sung Yoon, Suk-Ha Lee, Sunghoon Lee

**Affiliations:** 1 National Institute of Crop Science, Rural Development Administration, Suwon 441-857, Republic of Korea; 2 Personal Genomics Institute, Genome Research Foundation, Suwon 443-270, Republic of Korea; 3 Korean Bioinformation Center, Korea Research Institute of Bioscience and Biotechnology, Daejeon 306-809, Republic of Korea; 4 Theragen Bio Institute, TheragenEtex, Suwon 443-270, Republic of Korea; 5 National Instrumentation Center for Environmental Management, College of Agriculture and Life Science, Seoul National University, Seoul 151-921, Republic of Korea; 6 Department of Biology, Kyungpook National University, Daegu 702-701, Republic of Korea; 7 Department of Plant Science and Research Institute for Agriculture and Life Sciences, Seoul National University, Seoul 151-921, Republic of Korea

**Keywords:** Comparative genomics, Recombination, Whole-genome sequencing, Soybean, Crop plants

## Abstract

**Background:**

In contrast with wild species, cultivated crop genomes consist of reshuffled recombination blocks, which occurred by crossing and selection processes. Accordingly, recombination block-based genomics analysis can be an effective approach for the screening of target loci for agricultural traits.

**Results:**

We propose the variation block method, which is a three-step process for recombination block detection and comparison. The first step is to detect variations by comparing the short-read DNA sequences of the cultivar to the reference genome of the target crop. Next, sequence blocks with variation patterns are examined and defined. The boundaries between the variation-containing sequence blocks are regarded as recombination sites. All the assumed recombination sites in the cultivar set are used to split the genomes, and the resulting sequence regions are termed variation blocks. Finally, the genomes are compared using the variation blocks. The variation block method identified recurring recombination blocks accurately and successfully represented block-level diversities in the publicly available genomes of 31 soybean and 23 rice accessions. The practicality of this approach was demonstrated by the identification of a putative locus determining soybean hilum color.

**Conclusions:**

We suggest that the variation block method is an efficient genomics method for the recombination block-level comparison of crop genomes. We expect that this method will facilitate the development of crop genomics by bringing genomics technologies to the field of crop breeding.

## Background

**Figure 1:**
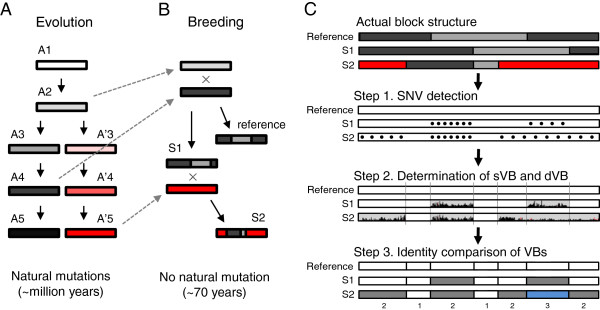
**Schematic diagram of soybean comparative genomics using variation profiles and blocks. (A)** Sequence diversity accumulation by random mutation. The flowchart shows the process of spontaneous mutation accumulation via long-term evolution. **(B)** Sequence diversity generation by recombination events during the cross-breeding process. The rectangles represent sequences. The different shades of colors represent the degrees of divergence. **(C)** The three steps that were used in the sequence comparisons of the cultivar genomes. In step 2, the dVBs are shaded in gray. The sections that are divided by gray vertical lines are VBs. In step 3, the VB types are shown in white, gray, and blue. At each chromosomal position, VBs of identical types are represented by the same color. For each VB, the total number of types that was observed is indicated at the bottom.

Crop cultivars have low levels of genetic diversity but high frequencies of recombination [1,2]. Cultivars contain specific sequence blocks in their chromosomes, which may be associated with artificially selected phenotypic variations from many generations of breeding. In contrast with wild species (Figure 1A), cultivar genomes consist of genetically reshuffled recombination blocks that arose from breeding ancestors (Figure 1B) [3-5]. For example, because the history of soybean breeding is too short for mutation accumulations (~70 years), the recombination blocks from common ancestors are usually identical in different cultivars, except for a small number of variations that are adjacent to the recombination points [6]. Therefore, as large-scale genome sequence data become available, the recombination block-based analysis is emerging as an efficient approach for comparing bred cultivar genomes with enough precision to detect molecular breeding targets.

Traditionally, recombination block identification has focused primarily on detecting linkage disequilibrium (LD) blocks, which can be determined by calculating the correlations of neighboring alleles. Combinations of alleles that are observed in the expected correlations are said to be in linkage equilibrium. In contrast, LD is higher-than-expected correlations between alleles at two loci that originate from single, ancestral chromosomes [7]. Based on the LD block calculation, it is possible to discover the haplotype block structure of a whole-genome [8]. Whole haplotype maps of a few model organisms have been created using LD blocks [9-11]. To generate a reliable whole-genome haplotype map, it is needed to calculate the pairwise linkage disequilibrium between the single nucleotide variations (SNVs) in many samples [12]. For this reason, maps have been generated from only a few model plants, including *Arabidopsis* and maize [13,14].

Recently, large-scale whole-genome sequencing by next-generation sequencing technology has been employed for recombinant inbred line (RIL) genotyping and even for linkage analyses to search for recombination breakpoints. However, the resulting data are prone to high error rates due to the relatively low levels of sequencing coverage that are typically attained. To overcome this drawback, a “bin” concept was introduced for the rice genome. Specifically, the sliding-window approach [15] and the hidden Markov model [16] were used to construct “bin maps” using the low-coverage sequence data. The bin map was successfully employed to reveal quantitative trait loci (QTL) that contained genes that are related to rice grain width [16]. However, there are some limitations to the usefulness of the bin-based method. Namely, almost all of the RIL individuals have to be sequenced to detect useful QTLs. Furthermore, a bin map of a RIL group cannot be reused for other RIL groups. However, if generally applicable comparative analysis methods are developed to identify bins, the effort and expense required to search for genes that are related to the target traits will be reduced.

Genomic analyses of various crops using whole-genome sequencing data have been previously reported. These include the analysis of 31 cultivated and wild soybean genomes using ~5× sequencing [17], genome-wide association studies of 950 rice varieties using ~1× sequencing [18], the identification of candidate regions that were selected during the domestication of 50 rice accessions using ~15× sequencing [19], and a breeding-associated genetic regulation analysis of 90 chickpea genomes using ~9.5× sequencing [20]. Integrating such large-scale genomic data will accelerate the screening of loci that are related to the valuable target traits.

Here, we propose a variation block (VB) method using next-generation sequencing data for the detection and analysis of recombination patterns in the genomes of crop species. The rationale behind the VB method is the existence of reshuffled sequence blocks within crop varieties that originated from a limited number of ancestral contributors and were introduced relatively recently over the course of the past several decades [21]. We suggest that such sequence blocks can be detected by identifying the SNV density profiles and that the resulting sequence blocks represent recombination blocks. We demonstrate the general applicability of the VB method by applying it to the publicly accessible genomes of 31 soybean and 23 rice accessions. Finally, by using a small number of insertion/deletion (indel) markers, each of which is specific to a recombination block, we identified a putative locus for soybean hilum color with minimal screening. With the increasing availability of genome sequences, the VB method shows promise as a useful genomic selection technology for crop improvement.

## Results

### Whole-genome sequencing of cultivated soybeans

Five soybean (*Glycine max* (L.) Merr.) cultivar genomes were sequenced. Two were parental cultivars (Baekun and Sinpaldal2), and two represented their crossed descendants (Daepoong and Shingi) (Additional file 1: Figure S1) and were used to detect inherited genome-wide recombination events. One of the descendants, Daepoong, has the highest productivity among Korean soybean cultivars (3.2 ton/ha) with excellent yield stability. We also used another elite line, Hwangkeum, which is not a member of this family but is popular for its attractive color and bean size. We produced paired-end DNA reads of 40–60-fold depths (Additional file 2: Table S1) for the five cultivar genomes and mapped them to the Williams 82 reference (PI 518671) [22]. The sequencing qualities of all the samples were high; 94%–99% of the cultivar sample reads were mapped to the reference, and 97%–99% of the reference genome was covered. A Williams 82 genome was also sequenced under the same conditions at a ~60-fold depth to reduce the base-calling noise. Additionally, *G. soja*[23], which is an undomesticated ancestor of *G. max*, was used as a control and analyzed with the same method.

### Comparative analysis procedure for cultivated soybean genomes using VB

The main objective of the VB method is to compare the reshuffled genome sequences of bred cultivars. A three-step process was applied to determine and compare the recombination blocks in the five soybean genomes (Figure 1C, Methods).

**Figure 2:**
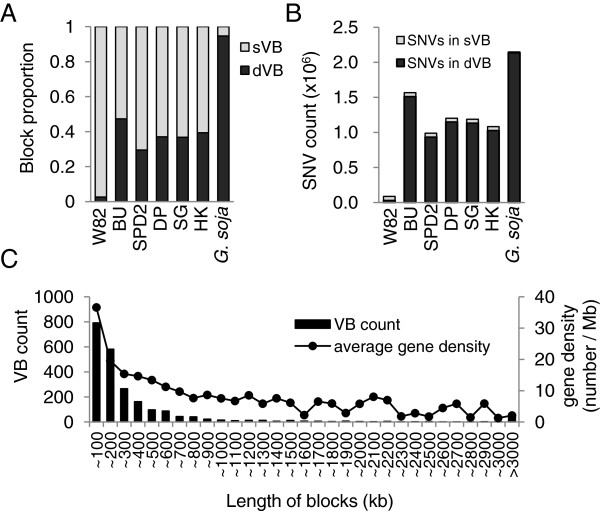
**Characteristics of soybean variation blocks. (A)** Proportions of the sVBs and dVBs in the soybean genomes with respect to length. **(B)** Counts of SNVs in the dVBs and sVBs. **(C)** Length distributions of the VBs and average gene densities of the corresponding blocks. W82, Williams 82; BU, Baekun; SPD2, Sinpaldal2; DP, Daepoong; SG, Shingi; HK, Hwangkeum.

• Step 1. SNV detection: SNVs and indels were detected by comparing bred soybean genomes with a reference genome (step 1 of Figure 1C). There were a total of 2,546,207 non-redundant SNVs (1,163,371–1,788,424 SNVs per cultivar) and a total of 486,010 small indels (225,815–348,642 indels per cultivar) in the five Korean soybean cultivars (Additional file 3: Table S2). Of these SNVs, 1,404,301 were novel (Additional file 4: Figure S2). The SNVs were highly clustered in certain chromosomal regions, whereas the regions that were genomically identical to the Williams 82 reference showed few or no SNVs.

• Step 2. Determination of VBs: Two types of blocks were determined: the sparse variation blocks (sVBs), which are identical or nearly identical to the reference sequence, and the dense variation blocks (dVBs), which contain many variations (step 2 of Figure 1C, Methods). The boundaries of the dVBs and sVBs were regarded as recombination sites. The VBs are thus defined as the sequence fragments that are split by all of the assumed recombination sites. In the five soybean genomes, 30–47% of the regions were dVBs (Figure 2A). As expected, in the resequenced Williams 82 genome, only a fraction of the regions (3%) were dVBs, which appeared probably due to individual differences between the two Williams 82 cultivars. By contrast, most of the regions in the *G. soja* genome (95%) were dVBs, indicating that the genetic pool of *G. soja* has been rarely used to breed Williams 82 and the five cultivars that were sequenced in this study.

**Figure 3:**
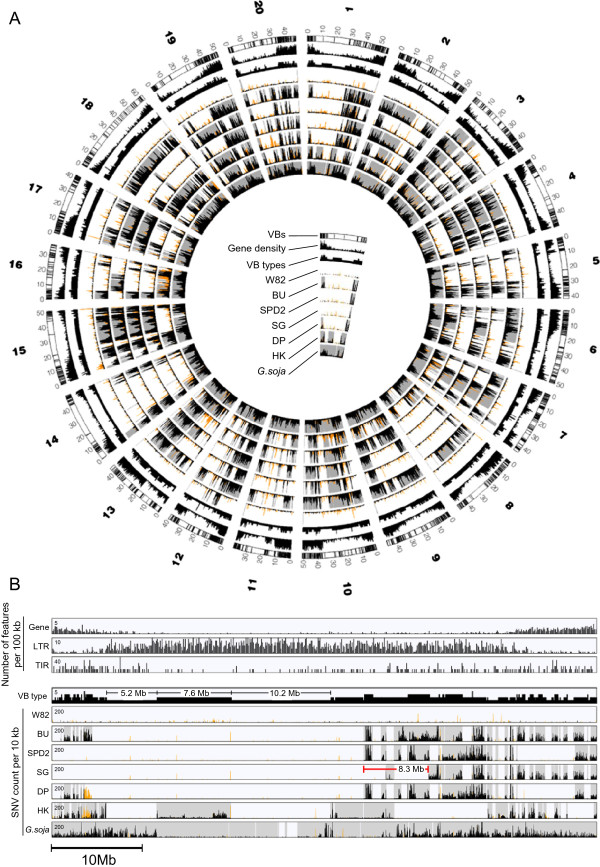
**Overview of chromosomal features and variations in soybean genomes. (A)** Circos plot of the block structures of the soybean genomes. **(B)** Chromosomal features and variations of chromosome 1. The numbers of genes, long terminal repeats (LTRs), and terminal inverted repeats (TIRs) are plotted in 100-kb windows. The SNV counts of the soybean cultivars are plotted in 10-kb windows. The shaded boxes represent the dVBs, and the remaining regions represent the sVBs. The black and orange SNV density peaks represent homo- and hetero-SNV densities, respectively. The numbers in the top left of the rectangular lanes are the maximum values of the y-axis. Five Korean soybeans were used to determine the VB type (Baekun, Sinpaldal2, Shingi, Daepoong, and Hwangkeum), and the minimum and maximum possible VB types are therefore one and five, respectively. VBs that were longer than 3 Mb are displayed. W82, Williams 82; BU, Baekun; SPD2, Sinpaldal2; DP, Daepoong; SG, Shingi; HK, Hwangkeum.

Most of the SNVs (96%) were located in dVBs, even though the dVBs occupied less than half of the genome (Figure 2B). A total of 4,332 sVBs and dVBs were identified in the five genomes, along with 4,132 boundary sites that demarcated the VBs. Figure 3 shows an overview of the block structures of all of the chromosomes (Figure 3A) and the detailed structure of chromosome 1 (Figure 3B). After eliminating redundancy, a total of 2,254 recombination sites and a set of 2,274 VBs covered the entire genomic region (Additional file 5: Table S3).

Recombination occurs more frequently in gene-rich regions than in gene-sparse regions [24-27]. Consistent with this, we confirmed a strong positive correlation (r = 0.85) between the gene density and VB density in the soybean genome. As a result, short VBs (<100 kb) were found mainly in regions with the highest gene densities (Figure 2C), whereas most of the very long VBs were located in heterochromatic regions (Figure 3B). This observation is consistent with previous studies that reported the suppression of recombination in the heterochromatin of various crops, such as sorghum and tomato [26,28].

**Figure 4:**
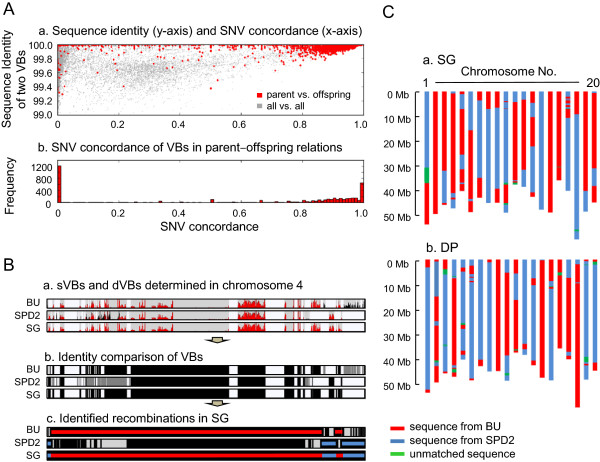
**Comparison of VB types between genomes and genetic diversities of plants. (A)** Grounds for determining the VB identity threshold. **(B)** Comparison of the VB types between Shingi and its parents, Baekun and Sinpaldal2. Panel a: red SNV density peaks represent the SNVs that are identical to those of Shingi. Panel b: black and gray regions represent the VBs whose types are identical to or different from those of Shingi, respectively. Panel c: red and blue regions depict the longest contiguous regions whose VB types are identical to Baekun and Sinpaldal2. **(C)** Recombination maps of the two descendants, Shingi and Daepoong. BU, Baekun; SPD2, Sinpaldal2; SG, Shingi.

Interestingly, there were identical variation patterns consistently appearing in the same regions of the examined genomes, indicating that these regions were inherited from common ancestors. The existence of boundaries that demarcate the dVBs and sVBs indicates that recombination has occurred at least once during breeding.• Step 3. Block comparison: The identity of each VB at specific locations was compared to all of the other VBs within the aligned column among the cultivar genomes (step 3 of Figure 1C). To compare the VBs among the genomes, two VBs were considered to be of an identical type and thus to have originated from a common parental genome when they had ≥99.8% sequence identity as well as ≥0.8 SNV concordance (Figure 4A). SNV concordance refers to the extent to which all the SNVs that are present in a VB are identical between genomes. Applying these thresholds, 98% of the VB types in the descendants (Daepoong and Shingi) were present in the parents (Baekun and Sinpaldal2). Figure 4B shows a comparison of VB types between Shingi and its parents. The resulting information was used to analyze the reshuffling patterns of the parental genomes in the descendants (Figure 4C).Almost all of the descendant chromosomal regions were present in the corresponding parental lines. However, some of the remaining (~1%) regions, such as an 8.3-Mb block in chromosome 1 of Shingi (shown in red in Figure 3B), were not observed similarly in the parental genomes, likely because the two individual parental plants that were used in this analysis are not the direct ancestors of the descendant cultivars.

### Robustness of VB detection with respect to sequencing depth

**Figure 5:**
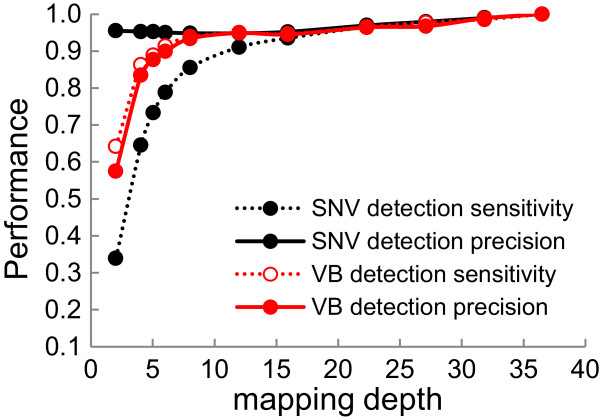
Mapping depth-dependent SNV and VB detection performances with respect to soybean reference genome.

We evaluated the performance of the VB detection at various sequencing depths and found that the VB method remained accurate even with 5-fold depths of mapping data (red curves in Figure 5). The performance of the VB method depends on that of the SNV calling, which, in turn, depends on the sequencing read depths. In our analysis, the sensitivity of the homologous SNV detection decreased dramatically at depths of <10-fold, amounting to 79% at a 6-fold depth and 73% at a 5-fold depth (black dotted curve in Figure 5). Nevertheless, even with the deterioration of the SNV-calling sensitivity, the sensitivity of the VB method remained greater than 90%, even at a 6-fold depth. At an 8-fold depth, the sensitivity and precision of VB detection reached approximately 95% of the highest values. Therefore, the VB method is very robust with respect to sequencing depths.

### VB-based analysis of publicly available soybean and rice genomes

To assess its general applicability, we applied the VB method to two sets of publicly available genomes of 31 soybean lines and 23 rice accessions [17,19].

The 31 publicly available soybean genomes consisted of 14 cultivated and 17 wild soybeans [17]. All of them were Chinese except for three cultivars from Brazil, Taiwan, and the USA. Nevertheless, for simplicity, all the 31 soybean types will be referred to collectively as “Chinese soybeans”. On average, the sequencing depth was 5-fold, which should thus allow for the attainment of at least 89% sensitivity and 88% precision according to depth-performance calibration (Figure 5). The VB method was successfully employed to analyze all but one cultivar genome that had distinctly fewer SNVs than did the others. As in the five Korean soybean cultivars, each of the 13 Chinese cultivar genomes also contained SNVs that were clustered in certain chromosomal regions with distinct SNV density profiles (Additional file 6: Figure S3). There were a total of 6,604 recombination sites, which was 2.9-fold higher than those that were observed in the five Korean soybean genomes, likely due to the higher number of analyzed genomes. More than half (62%, 1,390 of 2,254) of the recombination sites in the five Korean soybean genomes coincided with those in the 13 Chinese cultivar genomes. The remaining 38% may reflect differences in the genetic pools between the Chinese and Korean soybeans.

The 17 wild soybeans had much fewer sVBs than did the cultivated soybeans. An average of 31% of the genomic regions of the wild soybeans contained sVBs, and there were a total of 5,895 recombination sites, 43% (2,525) of which coincided with those of the 13 cultivars. These observations suggest that the 17 wild soybeans might have had opportunities to outcross with the ancestors of the cultivated soybeans. To assess the value of the wild soybean genomes as genetic resources, we determined the number of VBs that are shared between the cultivated and wild soybean genomes. Many (60–75%) VBs from the cultivar genomes were present in at least one of the wild soybean genomes. By contrast, far fewer (17–71%) VBs from the wild soybean genomes were present in the cultivar genomes (Additional file 7: Figure S4), suggesting that wild soybeans have a much more diverse genetic pool than do the cultivars.

**Figure 6:**
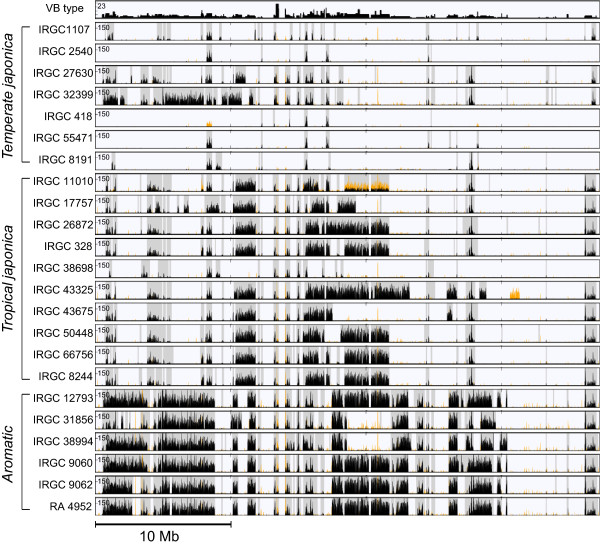
**Overview of chromosomal features and variations of chromosome 3 for 23 publicly available rice genomes.** The minimum and maximum possible VB types are one and twenty-three, respectively. This figure is represented in the same manner as in Figure 3B.

We next demonstrated that the VB method can be applied to monocot crops, such as rice. The procedures that were used in the soybean genome analysis were directly applied to rice genomes (Methods). We selected a total of 23 *Oryza sativa spp. japonica* genomes from 50 rice accessions that were reported in a previous study [19]. The following three varieties were included: seven *temperate japonica* (TEJ), ten *tropical japonica* (TRJ), and six *aromatic* (ARO) varieties. The average mapping depth of the sequencing data was approximately 14-fold, which is sufficient for accurate VB-based analysis (Figure 5). A total of 4,361 recombination sites were found in the rice genomes. Only 6.6% (289) of the sites were present in all three groups (Additional file 8: Figure S5). The seven *temperate* and ten *tropical japonica* genomes shared more than twice as many recombination sites as those that were shared with the six *aromatic* genomes. The resulting VB patterns indicated that the three groups are characterized by visually distinct SNV density profiles (Figure 6). The six *aromatic* varieties had the most distinct patterns. These findings are consistent with the conclusions of the original report, which were based on sequence homology [19].

### Quantification of genome diversity of crop population in terms of VBs

VBs were used to quantitatively estimate the genome diversities of crop populations. Whereas a sequence-based comparison infers the homology of genomes that have diverged via natural evolution (Additional file 9: Figure S6A), a block-based comparison determines whether two blocks from two genomes originated from the same parental genome (Additional file 9: Figure S6B).

**Figure 7:**
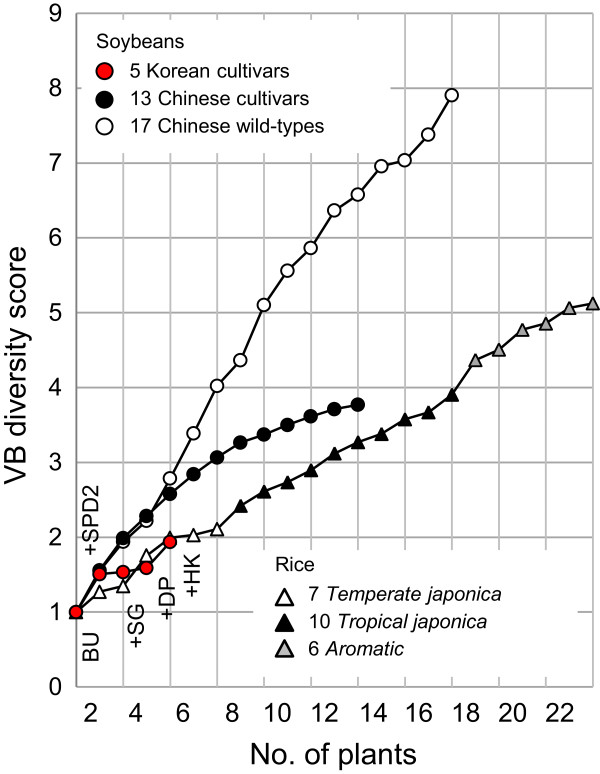
**VB diversity scores of soybean and rice genomes.** Plot of VB diversity scores with respect to the numbers of successively added soybean and rice genomes. BU, Baekun; SPD2, Sinpaldal2; DP, Daepoong; SG, Shingi; HK, Hwangkeum.

We examined the genome diversity by calculating the “VB diversity score”, which is defined as the number of unique VB types per the number of all VB sites in a genome. The steps for the calculation of the VB diversity score are illustrated in Additional file 10: Figure S7A–C. The resulting graphs revealed differences in diversity among the four groups of crops (Figure 7). The 13 Chinese soybean cultivar genomes produced the most smoothly increasing curve of VB diversity scores that eventually leveled off, indicating limited genome diversity in the cultivars. In comparison, wild soybeans produced a steeply increasing irregular curve that did not level off, indicating diverse VB compositions. The genetic diversity of the five Korean soybeans can also be seen (Figure 7). The two parental genomes (the first two red dots) had a score of 1.50, which was similar to the score of the first two genomes in the other soybean groups.

The three subgroups of rice genomes showed distinguishable patterns representing subgroups of unique recombination sites (Figure 7). The curve for *temperate japonica* was the most irregular among the three subgroups. The VB diversity scores of the rice genomes were much lower than those of the 13 cultivated soybean genomes.

### VBs as recombination blocks

**Figure 8:**
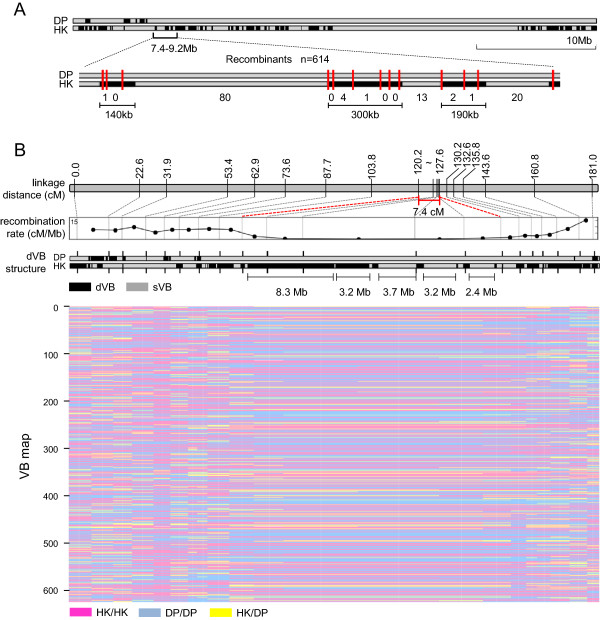
**Experimental confirmation of VBs as recombination blocks. (A)** Validation of the recombination frequencies of 614 F4 RILs on chromosome 8. The top two lanes depict chromosome 8 of the Daepoong and Hwangkeum cultivars. The black and gray sections in the lanes represent the dVBs and sVBs, respectively. The red vertical lines indicate the locations of the small indel markers that were used for the PCR validations. The numbers at the bottom represent the counts of recombination events that occurred in the 614 tested RILs. **(B)** Genetic and physical linkage maps and resulting recombination rates for chromosome 6. The recombination rates were calculated using 20 indel markers by mapping 614 RILs, through which the VB map was constructed. DP, Daepoong; HK, Hwangkeum.

Experimental validation supported that VBs are genuine recombination blocks. We determined the recombination frequencies in the 7.4–9.2 Mb region of chromosome 8 in 614 F4 progenies of RILs, which were the progenies of the cross that was made between Hwangkeum and Daepoong (Figure 8A). In this 1.8-Mb region, Hwangkeum had three dVBs that were 140 kb, 300 kb, and 190 kb in length alternating with three sVBs, whereas Daepoong had only one long sVB. The three sVB regions were of identical types between Daepoong and Hwangkeum.The analysis of the recombination frequencies in the 614 RILs showed that at least 6.6 times more recombination events occurred in the sVBs than in the dVBs. A total of nine and 113 recombination events occurred in 630 kb of dVBs and 1,190 kb of sVBs, respectively. This indicates that the more similar two sequences are, the more frequently recombination events occur. This relationship is consistent with a previous study that reported reduced recombination rates in regions surrounding non-alignable flanking sequences, such as SNVs and indels [29].

The genetic linkage distances and VB lengths were also compared. VB-specific indel markers were used to determine the recombination status of chromosomes 6 and 8 for the F4 RILs that are described above. Genetic and physical maps were constructed using markers that could distinguish between the dVB types of two cultivars, as described in the Methods section. We found very large VBs within very short genetic distances. Five VBs that were larger than 2 Mb were located within 7.4 cM of chromosome 6 (120.2–127.6 cM) (Figure 8B), and two large VBs of 12 Mb and 3.5 Mb were found within 13.9 cM of chromosome 8 (131.7–145.6 cM) (Additional file 11: Figure S8). These results indicate that VBs are recombination units that rarely split.

### Identification of the locus determining soybean hilum color

**Figure 9:**
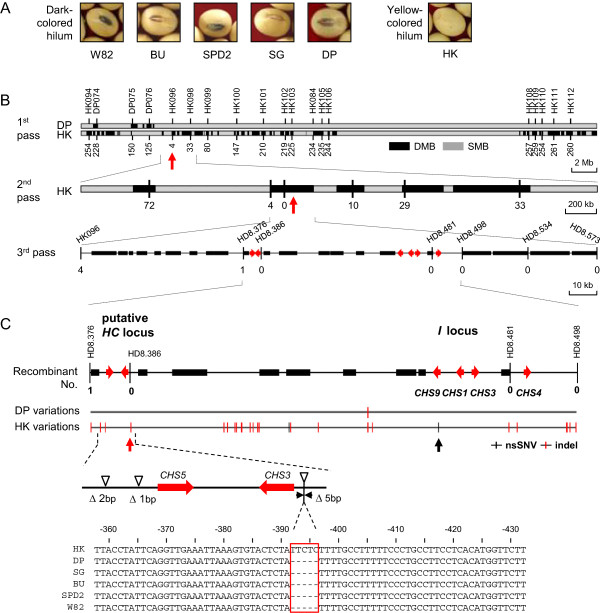
**VB-based mapping of hilum color-determining locus. (A)** Hilum colors of the soybeans. **(B)** The three-step process for dVB-based locus mapping. The black sectors in the 1st and 2nd passes represent the dVBs. The vertical red arrows indicate the identified dVB that contains the hilum color-determining locus. The *CHS* genes are shown as red horizontal arrows. The other genes are shown as black bars. The numbers below the chromosome lanes represent the counts of the recombinants, whose hilum color does not match with its genotype as confirmed by indel markers. **(C)** Genome structure of *I* and putative *HC* loci. The sequence variations of Daepoong and Hwangkeum are indicated at the bottom lines. The vertical black bars and arrows represent a non-synonymous SNV of *CHS9* in the *I* locus and a 5-bp deletion of the *CHS3* promoter in the HC locus, respectively. A multiple sequence alignment shows the promoter region of the *CHS3* gene. The five-base deletion position is indicated as a red box.

Finally, we showed the practicability of the VB method for map-based screening by identifying a putative locus that determines soybean hilum color. Although hilum color is thought to be related to the *I* locus on chromosome 8 [30-32], the exact locus has not yet been identified. Using the VB method, we attempted to detect the hilum color-determining locus. As a result, the entire screening process for narrowing down the target region required only the following three steps in the 614 F4 progenies of RILs that were selected from the cross of Hwangkeum (yellow hilum) and Daepoong (brown hilum) (Figure 9A).

We began by selecting 32,000 indel markers that were longer than four base pairs, which allowed for the discrimination of the VBs from Hwangkeum from those from Daepoong (Methods). Among these indel markers, the first screening was performed with 464 markers in all 20 chromosomes, each of which represented a single VB that was identified from the Hwangkeum and Daepoong genomes. The HK096 marker on chromosome 8 had the highest correlation (r = 0.993) with the hilum color phenotype (Additional file 12: Figure S9 and Figure 9B, 1st pass, indicated by the upward red arrow). The second step was performed with five markers that represented one of the five dVBs near HK096. Because recombination events within the dVBs occurred rarely, as described in the previous section, one marker was sufficient to represent one dVB. We found that the target locus was in a 300-kb dVB spanning the 8.28–8.58 Mb region (Figure 9B, 2nd pass, indicated by the upward red arrow), which showed the highest correlation with the hilum color phenotype. The final step was performed with seven markers (Figure 9B, 3rd pass) to locate the exact hilum color-determining locus, which was located in a very large block of approximately 197 kb.

To identify the locus that determines hilum color, a comparative analysis was conducted. Soybean seed coats and hilum color are regulated by chalcone synthase (*CHS*) genes that control the anthocyanin and proanthocyanidin pigments via a posttranscriptional mode of gene silencing [33,34]. Therefore, we compared the sequence differences for Daepoong and Hwangkeum of *CHS*s in the *I* locus, which is located close to the HD8.481 marker in the 197-kb block. There were no significant sequence differences except for one non-synonymous SNV in *CHS9* of the *I* locus (Figure 9C, indicated by the upward black arrow), which cannot affect the regulation of gene silencing mechanisms between two sequences in this region. Instead, we found that two highly similar (98.0%) genes, *CHS3* and *CHS5*, were present as inverted repeats near the HD8.386 marker downstream of the *I* locus (Figure 9C). Except for these *CHSs*, there were no genes that are involved in the anthocyanin metabolic pathway in this 197-kb block. We concluded that an inverted repeat structure of *CHS3* and *CHS5* is a candidate locus for hilum color determination.

We compared the sequence differences among the six soybean cultivars in the 197-kb block and found that the only notable difference was a 5-bp deletion in the *CHS3* promoter regions of every genome except for Hwangkeum (Figure 9C, indicated by the upward red arrow and multiple sequence alignment). If the expression of *CHS3* and *CHS5* is regulated by a gene silencing mechanism that is similar to that of the *i*^*i*^ allele on the *I* locus, then these two genes may be silenced in Hwangkeum, which has an intact *CHS3* promoter. However, if the 5-bp deletion inhibits promoter activity, *CHS5* would not be silenced due to the absence of interfering RNA. Therefore, the 5-bp deletion may produce dark-colored hila in all of the soybeans except for Hwangkeum. We also examined 86 other soybean cultivars and found that the 5-bp deletion perfectly correlated with colored hila (Additional file 13: Table S4). We named this putative locus *HC*, in which the *I*^*h*^ and *i*^*h*^ alleles control the yellow and brown hilum colors, respectively. These results suggest that the seed color phenotype of Hwangkeum is determined by two linked loci, consisting of the *i*^*i*^ allele (yellow seed coat) of the *I* locus and the *I*^*h*^ allele (yellow hilum color) of the *HC* locus.

## Discussion

We proposed an efficient recombination block detection method that is based on genomic variation patterns. This method provides key information for the map-based screening of bred cultivars. The VB method can be applied to various crop species that have available reference genomes. In this study, representative monocot and dicot model crops, rice and soybeans, were successfully analyzed using the VB method.

The VB-based comparative genomics method has several advantages over other methods. The first advantage is that the samples can be compared directly at the block level. Therefore, an agricultural trait-associated locus or gene can be identified with reduced screening efforts by using a small number of markers that represent the VBs. We demonstrated this advantage by identifying a putative locus determining hilum color in soybeans. There is no need to use markers on the VBs of the same type that are present in two genomes, such as the rear half of Gm02, the middle of Gm14, and the front half of Gm20 in the 1st pass (Additional file 12: Figure S9). The VBs were also useful in the 2nd pass of the screening. Since VBs are the units that rarely split by recombination, only one marker can represent one dVB. In this report, only five markers were used to screen five dVBs in the 2 Mb target region (Figure 9B). These results indicate that the VB method enables the minimal use of molecular markers and efficient screening via an accurate recombination map. Map-based cloning in soybeans has thus far identified few genes [35], and this method represents a significant advancement.

The second advantage is that the VB method does not depend on the number of samples. In contrast to other statistical methods, such as the LD analysis, the VB method can identify recombination blocks using only one genome if a reference genome sequence is available. This feature is especially useful in genomic screening against RIL populations. Even with the availability of only two parental genome sequences, researchers can still define the VB blocks, compare the two genomes at the recombination block level, and predict the possible recombination sites that are likely to occur in the RIL population.

The third advantage is that the VB method can accurately detect recombination blocks even with low-depth sequencing data. The VB method detected recombination blocks with more than 90% sensitivity and precision even with 6-fold depth data.There were many chromosomal regions that have the same types of VBs across multiple genomes, reflecting the limited genetic diversity resulting from artificial selection during the breeding history. As shown in Figure 8A, the identical regions showed 6.6 times higher recombination rates than those of the other regions. In contrast, recombination occurs randomly in wild-type genomes, which rarely share identical regions with each other. These results imply that low genetic diversity can lead to non-random recombination, followed by the conservation of VBs.

## Conclusions

In conclusion, we propose the VB method for the identification and comparison of the reshuffled genome sequences of bred cultivars. We demonstrated the usefulness and generality of the VB method by applying it to the publicly available genomes of 31 soybeans and 23 rice accessions. The VB-representing indel markers accurately identified a putative locus that determines the yellow hilum color in soybean. Thus, the VB method is applicable in the cloning of agronomically important genes in a simple and fast manner.

## Methods

### DNA extraction and massively parallel sequencing

The total genomic DNA was extracted from the leaf tissues of the soybean cultivars using the cetyltrimethyl ammonium bromide method [36]. DNA libraries that were constructed from Daepoong and Hwangkeum were sequenced using an Illumina GAIIx sequencer (Illumina Inc., San Diego, CA, USA). Four libraries of three Korean cultivars (Baekun, Shingi, and Sinpaldal2) and Williams 82 were sequenced using an Illumina HiSeq 2000 sequencer. Each sequenced sample was prepared using Illumina protocols. Paired-end 101-bp or 104-bp reads were generated.

### Reads alignment and variation detection

The sequence reads of five Korean soybean cultivars and twenty-three rice accessions were aligned to the Gmax109 soybean reference genome [22] and the IRGSP Build 4 rice reference genome [37], respectively, using the BWA algorithm [38] ver. 0.5.9. Two mismatches were permitted in the 45-bp seed sequence. To remove the PCR duplicates of the sequence reads, which can be generated during the library construction process, the rmdup command of SAMtools [39] was used. The aligned reads were realigned at indel positions with the GATK [40] IndelRealigner algorithm to enhance the mapping quality. The GATK TableRecalibration algorithm was used to recalibrate the base quality scores. The 23 rice genome sequence datasets were downloaded from the NCBI Short Read Archive (http://www.ncbi.nlm.nih.gov/Traces/sra/sra.cgi?study=SRP003189). The SNVs of 31 Chinese soybeans were downloaded from the BGI ftp site (ftp://public.genomics.org.cn/BGI/soybean_resequencing/).

### Analysis of SNVs and small indels

The SNVs were called and filtered using the UnifiedGenotyper and VariantFiltration commands in GATK, respectively. The options that were used for SNP calling were a minimum of 5- to a maximum of 200-read mapping depths with a consensus quality of 20 and a prior likelihood of heterozygosity value of 0.01 for the soybean genomes. For the rice genomes, the same options as in the soybean genomes were used except for the read mapping depths, in which a minimum of 3 to a maximum of 150 were used.

### Recombination block detection

The homozygous SNV densities for 10-kb bins were calculated for six cultivar genomes (Williams 82, Baekun, Shingi, Sinpaldal2, Hwangkeum, and Daepoong). The SNVs that were detected in Williams 82 were regarded as false positives and excluded from the other cultivars. Bins with < 4 SNVs were defined as members of similar-to-standard recombination blocks (sRBs), and those with ≥ 4 SNVs were defined as different-to-standard recombination blocks (dRBs). Neighboring dRBs that were ≥ 90 kb were merged into one dVB. Similarly, sRBs that were ≥ 30 kb were merged into one sVB. The distances of 90 kb and 30 kb were determined heuristically. VBs were defined as the sequence fragments that were split by all of the observed recombination sites, which were the boundaries of the dVBs and sVBs.

### Determination of thresholds for VB identity comparison

We employed two types of filters to determine whether two VBs from different genomes were of identical type. The first filter was sequence identity. When inherited, most of the VBs in the descendant genomes had ≥ 99.8% identity to those of the parents (red dots of top panel in Figure 4A). The second filter was the concordance of the SNV sets in the VBs. When inherited, most of the SNV concordances were ≥ 0.80 (bottom panel of Figure 4A). If two VBs had ≥ 99.8% sequence identity and ≥ 0.80 SNV concordance, they were considered to be of the same type, which originated from a common ancestor.

### Performance test of SNV and VB detection

The sequence reads of the cultivar soybean Baekun were divided into small read sets, each of which was able to cover a 2-fold depth. With the incremental addition of each small read set, a series of sequence read sets was generated, resulting in mapping depths of 2.0×, 4.0×, 5.0×, 6.0×, 7.9×, 9.8×, 11.8×, 15.6×, 22.0×, 26.7×, 31.4×, and 36.1×. These read sets were aligned to the Gm109 soybean reference genome, and the SNVs were called as described above, except for the minimum two-read mapping depth. By using the resulting SNV sets, the VB blocks were detected and compared as described above. The SNVs and VBs that were detected from the largest read set (36.1×) were used as standards for the performance assessment. Only the homozygous SNVs were used in the performance assessment and VB detection.

### Indel marker analysis

The PCR analysis was performed using 10-μl reaction mixtures containing 20 ng of total genomic DNA, 0.4 μM of primer, and 5 μl of GoTaq Green Master Mix (Promega, Madison, WI, USA) using a Biometra T1 Thermal Cycler (Biometra, Goettingen, Germany). The PCR conditions were as follows: initial denaturation for 5 min at 95°C; 34 cycles of 30 sec at 95°C, 30 sec at 48°C, and 30 sec at 72°C; and a final extension for 7 min at 72°C. The PCR products were separated by 3% agarose gel electrophoresis.

### Construction of genetic and physical maps

A genetic map of the Hwangkeum/Daepoong population was constructed using JoinMap ver. 4.0 (http://www.kyazma.nl/index.php/mc.JoinMap). Prior to the map construction, all of the segregated markers were subjected to the chi-square test using the locus genotype frequencies feature of JoinMap. The linkage groups of chromosome 6 and 8 were separated using an independence LOD (logarithm of the odds) score of 3.0. The marker orders within the linkage groups were established using the regression-mapping algorithm. The recombination values were converted to genetic distances (cM) using the Kosambi mapping function [41].

### Availability of supporting data

The raw reads for this project have been deposited in the Sequence Read Archive (SRA) project under the accession number SRA052312.

## Competing interests

The authors declare that they have no competing interests.

## Authors’ contributions

YHK, HMP, and SL conceived the project and planned the experiments. YHK and SL supervised the research. TYH, SKL, and MSC performed the genetic and physical mapping. MJS, KHJ, HTY, and YUK conducted the field experiments. IYC and SHL performed the resequencing analysis. YSK, HSY, SLK, WHK, HKC, SJ, HK, YSC, HK, BG, DL, YS, JP, and SL performed the data analysis. SL, YHK, HMP, and SH wrote the manuscript. All authors read and approved the final manuscript.

## Supplementary Material

Additional file 1: Figure S1 Breeding history of the five soybean cultivars. The black boxes represent the soybeans that were analyzed. The acronyms in parentheses are used in place of the full cultivar names in all figures and tables. Click here for file

Additional file 2: Table S1 Statistics of the short-read sequencing analysis results for the six cultivated soybean plants. Click here for file

Additional file 3: Table S2 Statistics of the SNVs and indels of the six cultivated soybean plants. Click here for file

Additional file 4: Figure S2 Venn diagram of the SNVs in the five Korean soybean and other publicly available soybean cultivars. Click here for file

Additional file 5: Table S3 List of the soybean VBs. Click here for file

Additional file 6: Figure S3 Overview of the chromosomal features and variations of chromosome 1 in 30 publicly available soybean genomes, which are represented in the same manner as in Figure 3B. Click here for file

Additional file 7: Figure S4 Extent of overlap between the VB pools of cultivated and wild soybean accessions. Click here for file

Additional file 8: Figure S5 Venn diagram of the number of recombination sites in 23 cultivated *Oryza sativa* genomes. Click here for file

Additional file 9: Figure S6 Differences between the sequence-based comparison **(A)** and block-based comparison **(B)**. Click here for file

Additional file 10: Figure S7 Method for calculation of VB diversity score of a population. **(A)** Schematic diagram of the procedure. The horizontal lanes represent the same chromosome in different cultivars. At each VB site, the same types are represented by the same color. With the successive addition of sample, the newly-appeared types of VBs are marked as red dots. **(B)** The resulting table of VB diversity score. The second column shows the sample sets at each successive addition of sample. The third column shows the number of VB types found in the sample set. The denominator “7” in the last column is the total of all of the VB sites on the chromosome. **(C)** The resulting plot of the VB diversity scores represents the genetic diversities of the population. Click here for file

Additional file 11: Figure S8 Linkage maps and recombination rates of chromosome 8. **(A)** Overview of the chromosomal features and variations of chromosome 8, which are represented in the same manner as in Figure 3B. **(B)** Genetic and physical linkage maps and the resulting recombination rates of chromosome 8. The recombination rates were calculated using 19 indel markers by mapping 614 RILs, through which the VB map was constructed. DP, Daepoong; HK, Hwangkeum. Click here for file

Additional file 12: Figure S9 Genome-wide linkage analysis for screening putative hilum color-determining loci. The LOD scores of 20 chromosomes were plotted. The X- and Y-axes represent the marker positions and LOD scores, respectively. Click here for file

Additional file 13: Table S4 Hilum colors and the HD8.386 indel marker analysis results for the 86 soybean cultivars. Click here for file
