# Supplementary figures and images for: Variation block-based genomics method for crop plants
Source: BMC Genomics. 2014 Jun 15;15:477. doi: 10.1186/1471-2164-15-477 (PMC4229737; doi:10.1186/1471-2164-15-477)

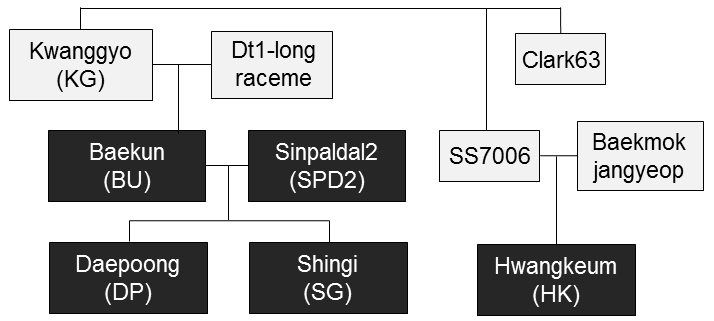

Supplement: Additional file 1: Figure S1 — Breeding history of the five soybean cultivars. The black boxes represent the soybeans that were analyzed. The acronyms in parentheses are used in place of the full cultivar names in all figures and tables. [file 1471-2164-15-477-S1.png]

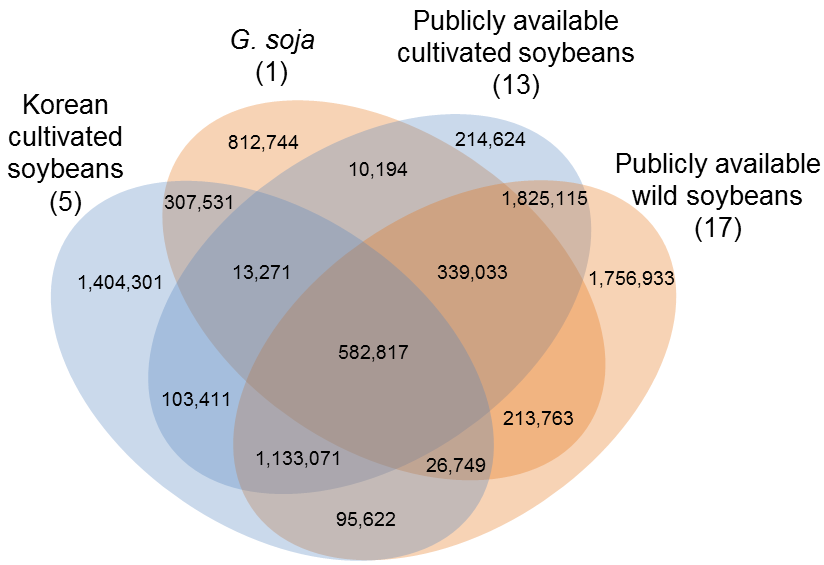

Supplement: Additional file 4: Figure S2 — Venn diagram of the SNVs in the five Korean soybean and other publicly available soybean cultivars. [file 1471-2164-15-477-S4.png]

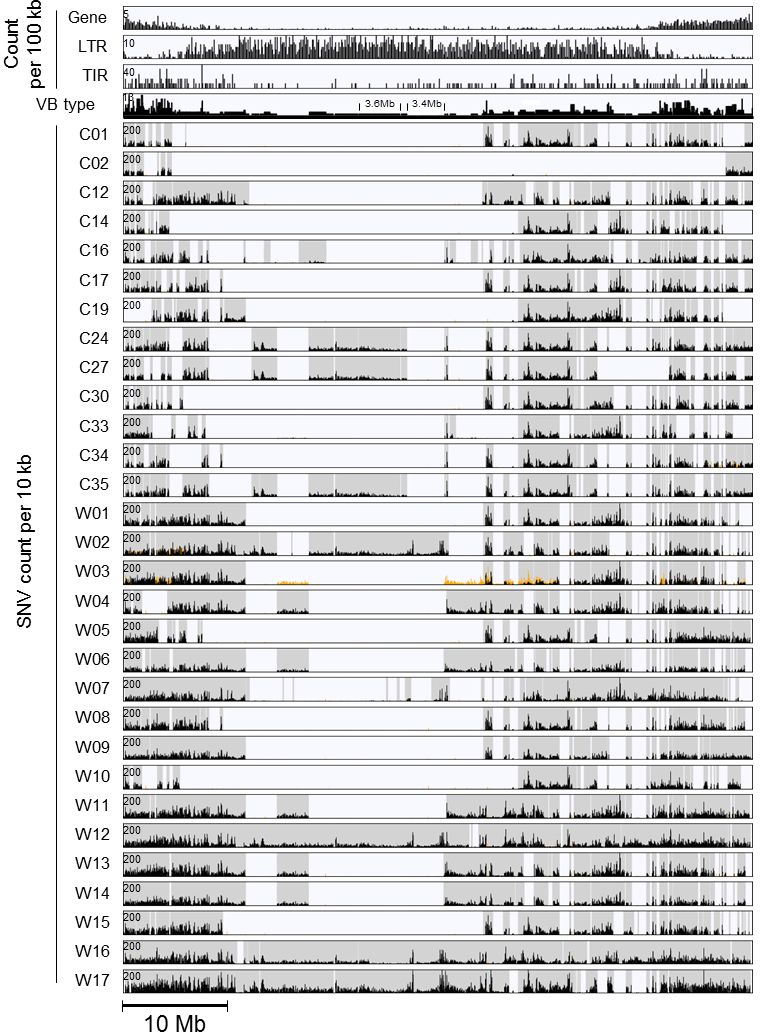

Supplement: Additional file 6: Figure S3 — Overview of the chromosomal features and variations of chromosome 1 in 30 publicly available soybean genomes, which are represented in the same manner as in Figure 3B. [file 1471-2164-15-477-S6.png]

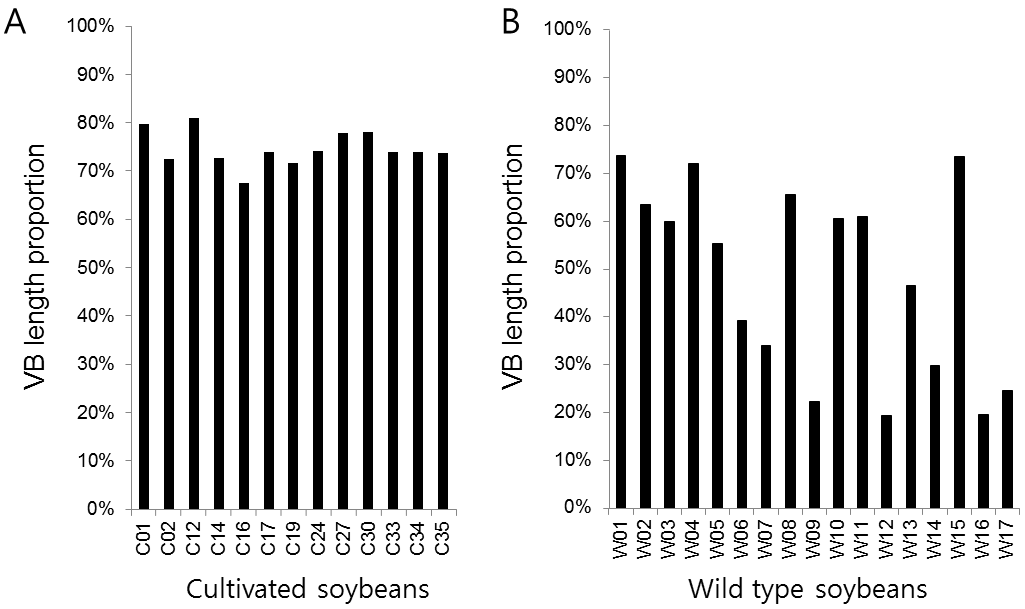

Supplement: Additional file 7: Figure S4 — Extent of overlap between the VB pools of cultivated and wild soybean accessions. [file 1471-2164-15-477-S7.png]

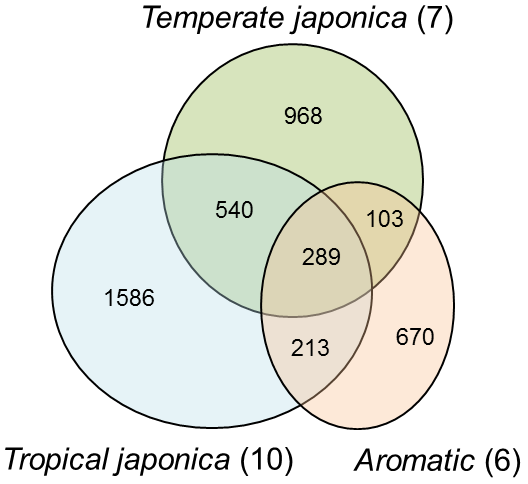

Supplement: Additional file 8: Figure S5 — Venn diagram of the number of recombination sites in 23 cultivated Oryza sativa genomes. [file 1471-2164-15-477-S8.png]

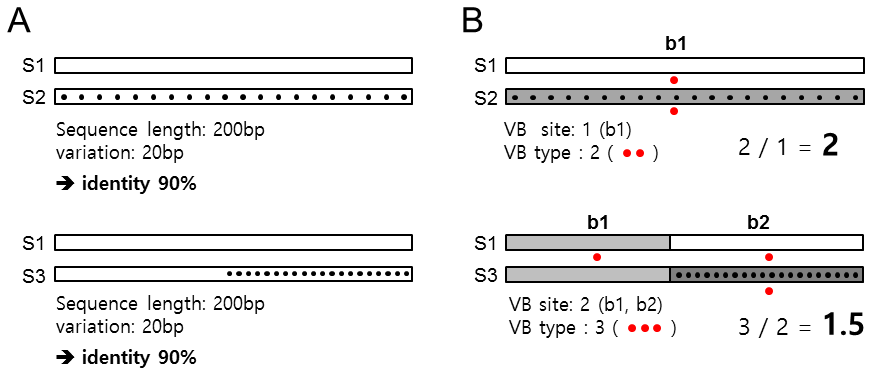

Supplement: Additional file 9: Figure S6 — Differences between the sequence-based comparison (A) and block-based comparison (B). [file 1471-2164-15-477-S9.png]

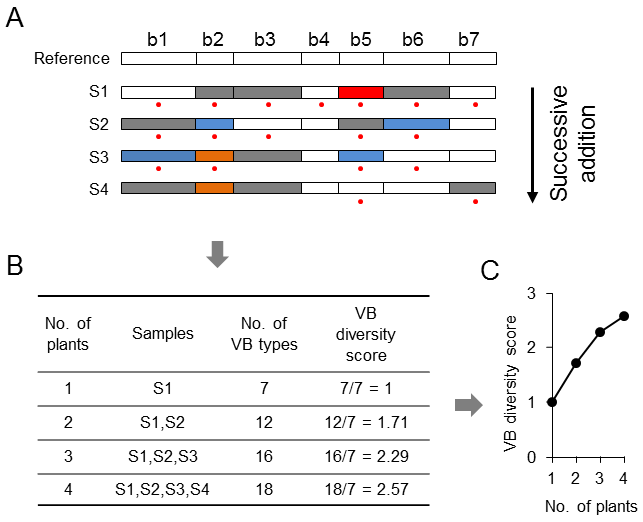

Supplement: Additional file 10: Figure S7 — Method for calculation of VB diversity score of a population. (A) Schematic diagram of the procedure. The horizontal lanes represent the same chromosome in different cultivars. At each VB site, the same types are represented by the same color. With the successive addition of sample, the newly-appeared types of VBs are marked as red dots. (B) The resulting table of VB diversity score. The second column shows the sample sets at each successive addition of sample. The third column shows the number of VB types found in the sample set. The denominator “7” in the last column is the total of all of the VB sites on the chromosome. (C) The resulting plot of the VB diversity scores represents the genetic diversities of the population. [file 1471-2164-15-477-S10.png]

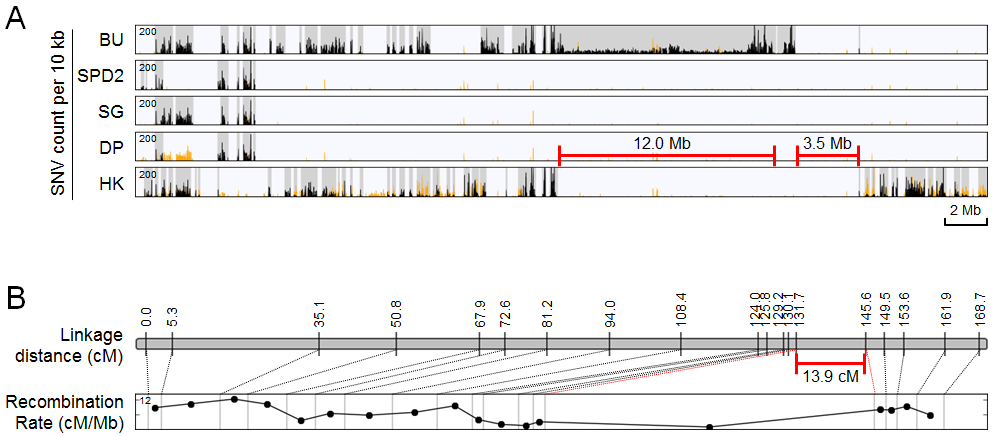

Supplement: Additional file 11: Figure S8 — Linkage maps and recombination rates of chromosome 8. (A) Overview of the chromosomal features and variations of chromosome 8, which are represented in the same manner as in Figure 3B. (B) Genetic and physical linkage maps and the resulting recombination rates of chromosome 8. The recombination rates were calculated using 19 indel markers by mapping 614 RILs, through which the VB map was constructed. DP, Daepoong; HK, Hwangkeum. [file 1471-2164-15-477-S11.png]

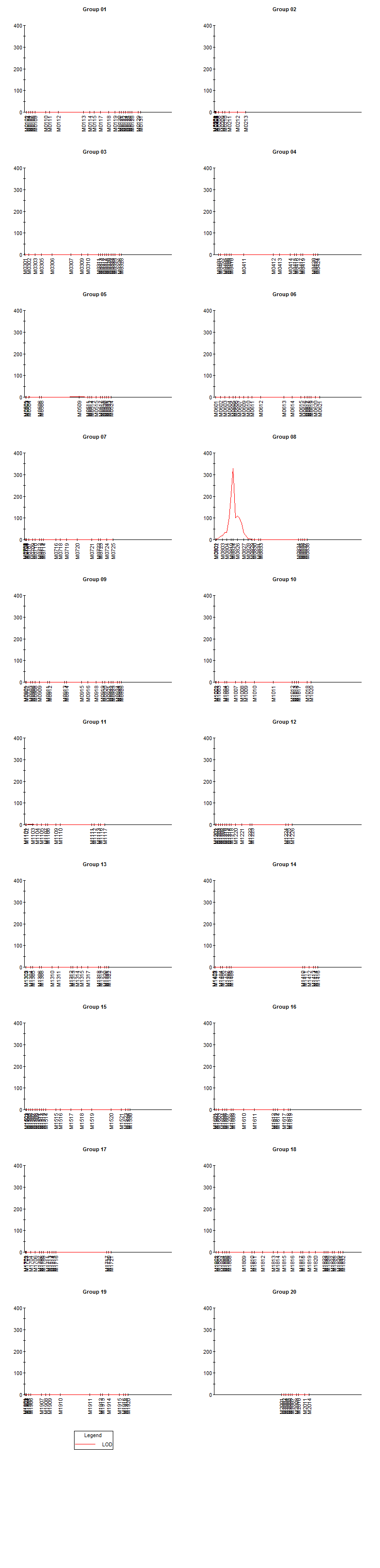

Supplement: Additional file 12: Figure S9 — Genome-wide linkage analysis for screening putative hilum color-determining loci. The LOD scores of 20 chromosomes were plotted. The X- and Y-axes represent the marker positions and LOD scores, respectively. [file 1471-2164-15-477-S12.png]
