# Supplementary material for: Variation block-based genomics method for crop plants
Source: BMC Genomics. 2014 Jun 15;15:477. doi: 10.1186/1471-2164-15-477 (PMC4229737; doi:10.1186/1471-2164-15-477)
Supplement: Additional file 2: Table S1 — Statistics of the short-read sequencing analysis results for the six cultivated soybean plants. [file 1471-2164-15-477-S2.pdf]

Table S1 - Statistics of short-read sequencing analysis results of six cultivated soybean plants.

Sample Name	Summary of sequencing and mapping						
	total base (Gb)	sequencing depth (fold)	mapped base (Gb)	mapping depth (fold)	mapping rate	mapping coverage (>=1)	mapping coverage (>=5)
Williams 82 (W82)	58.22	60.18	57.62	59.56	98.9%	98.5%	98.3%
Baekun (BU)	58.96	60.94	56.04	57.92	95.1%	97.0%	96.2%
Sinpaldal2 (SPD2)	58.6	60.57	57.82	59.76	98.7%	97.7%	97.1%
Shingi (SG)	59.53	61.53	55.78	57.65	93.7%	97.5%	96.8%
Daepoong (DP)	40.32	41.67	38.97	40.28	96.7%	96.9%	93.6%
Hwanggeum (HK)	44.29	45.78	42.19	43.61	95.3%	97.3%	94.7%
