# Supplementary material for: Variation block-based genomics method for crop plants
Source: BMC Genomics. 2014 Jun 15;15:477. doi: 10.1186/1471-2164-15-477 (PMC4229737; doi:10.1186/1471-2164-15-477)
Supplement: Additional file 3: Table S2 — Statistics of the SNVs and indels of the six cultivated soybean plants. [file 1471-2164-15-477-S3.pdf]

Table S2 - Statistics of SNVs and indels of six cultivated soybean plants.

Sample Name	Summary of variation			
	homo SNV	hetero SNV	homo indel	hetero indel
Williams 82 (W82)	86,172	103,149	35,903	22,397
Baekun (BU)	1,566,469	221,955	307,116	41,526
Sinpaldal2 (SPD2)	988,173	175,198	194,711	31,104
Shingi (SG)	1,188,325	195,693	239,875	33,835
Daepoong (DP)	1,202,056	248,111	198,126	31,544
Hwanggeum (HK)	1,082,204	487,530	202,012	66,223
