# Supplementary material for: Variation block-based genomics method for crop plants
Source: BMC Genomics. 2014 Jun 15;15:477. doi: 10.1186/1471-2164-15-477 (PMC4229737; doi:10.1186/1471-2164-15-477)
Supplement: Additional file 5: Table S3 — List of the soybean VBs. [file 1471-2164-15-477-S5.pdf]

Table S3 - List of soybean VBs.
VB types were named by the initial of cultivar that has unique VB type, in the order of Williams 82 (W82), Baekun (BU), Sinpadal2 (SPD2), Shingi (SG), Daepoong (DP), Hwangkeum (HK), and G. soja.

VB ID	Chromosome	Start position	End position	Length	Gene No.	VB type							SNV No.						
						W82 (W)	BU (B)	SPD2 (S)	SG (G)	DP (D)	HK (H)	G.soja (I)	W82	BU	SPD2	SG	DP	HK	G.soja
Gm01.VB.1	Gm01	0	280000	280000	13	W	W	W	W	W	W	I	20	19	21	15	24	25	681
Gm01.VB.2	Gm01	280000	350000	70000	11	W	W	W	W	W	H	I	1	1	1	1	1	113	200
Gm01.VB.3	Gm01	350000	560000	210000	29	W	W	W	W	W	W	I	6	9	6	7	7	8	642
Gm01.VB.4	Gm01	560000	590000	30000	5	W	W	W	W	W	H	I	0	0	0	0	0	49	104
Gm01.VB.5	Gm01	590000	700000	110000	9	W	W	W	W	W	W	I	1	1	2	2	1	6	249
Gm01.VB.6	Gm01	700000	940000	240000	24	W	W	W	W	W	H	I	38	38	38	29	32	341	520
Gm01.VB.7	Gm01	940000	960000	20000	1	W	B	W	W	B	H	I	0	73	0	0	72	72	35
Gm01.VB.8	Gm01	960000	1020000	60000	7	W	W	W	W	W	H	I	1	2	1	1	2	182	132
Gm01.VB.9	Gm01	1020000	1260000	240000	24	W	W	W	W	W	W	I	26	34	21	26	29	32	352
Gm01.VB.10	Gm01	1260000	1270000	10000	0	W	B	W	W	B	W	I	2	36	2	2	32	2	20
Gm01.VB.11	Gm01	1270000	1760000	490000	43	W	B	W	W	B	H	I	62	1432	66	55	1353	1126	971
Gm01.VB.12	Gm01	1760000	1900000	140000	10	W	B	W	W	B	W	I	3	398	3	3	354	5	269
Gm01.VB.13	Gm01	1900000	2050000	150000	12	W	W	W	W	W	W	I	4	8	4	3	8	10	311
Gm01.VB.14	Gm01	2050000	2190000	140000	12	W	W	W	W	W	H	I	12	20	12	11	16	614	490
Gm01.VB.15	Gm01	2190000	2360000	170000	17	W	B	W	W	B	H	I	14	925	14	17	854	523	589
Gm01.VB.16	Gm01	2360000	2530000	170000	10	W	W	W	W	W	H	I	14	21	13	13	22	92	379
Gm01.VB.17	Gm01	2530000	2560000	30000	3	W	B	W	W	B	H	I	0	96	0	0	91	115	61
Gm01.VB.18	Gm01	2560000	2670000	110000	4	W	B	W	W	D	W	I	18	185	18	18	139	19	333
Gm01.VB.19	Gm01	2670000	2710000	40000	3	W	B	W	W	B	H	I	0	177	1	1	169	228	97
Gm01.VB.20	Gm01	2710000	2870000	160000	15	W	W	W	W	W	H	I	12	13	12	11	10	973	444
Gm01.VB.21	Gm01	2870000	3000000	130000	10	W	W	W	W	W	W	I	14	20	16	13	18	18	353
Gm01.VB.22	Gm01	3000000	3030000	30000	2	W	B	W	W	B	H	I	0	190	0	0	177	207	106
Gm01.VB.23	Gm01	3030000	3040000	10000	1	W	B	W	W	W	W	I	0	5	0	0	4	1	17
Gm01.VB.24	Gm01	3040000	3270000	230000	22	W	W	W	W	W	W	I	3	12	3	3	12	47	782
Gm01.VB.25	Gm01	3270000	3330000	60000	2	W	B	W	W	D	H	I	4	554	7	4	469	89	187
Gm01.VB.26	Gm01	3330000	3390000	60000	5	W	B	W	W	W	H	I	5	479	5	4	6	74	192
Gm01.VB.27	Gm01	3390000	3490000	100000	5	W	B	W	W	W	W	I	31	437	36	17	18	25	216
Gm01.VB.28	Gm01	3490000	4120000	630000	43	W	B	W	W	W	H	I	54	3367	54	53	39	1002	2393
Gm01.VB.29	Gm01	4120000	4660000	540000	36	W	W	W	W	W	H	I	10	10	10	10	8	1591	2626
Gm01.VB.30	Gm01	4660000	4850000	190000	12	W	W	W	W	W	W	I	1	2	1	2	1	14	617
Gm01.VB.31	Gm01	4850000	5340000	490000	20	W	W	W	W	W	H	I	70	67	72	54	56	1616	1350
Gm01.VB.32	Gm01	5340000	5450000	110000	6	W	W	W	W	W	W	I	1	1	1	1	1	9	151
Gm01.VB.33	Gm01	5450000	5570000	120000	5	W	W	W	W	W	H	I	5	5	6	5	1	1149	584
Gm01.VB.34	Gm01	5570000	10760000	5190000	133	W	W	W	W	W	W	I	249	240	261	218	194	526	16455
Gm01.VB.35	Gm01	10760000	18390000	7630000	75	W	W	W	W	W	H	I	412	486	511	355	474	10969	4810
Gm01.VB.36	Gm01	18390000	28610000	10220000	102	W	W	W	W	W	W	I	418	367	410	343	332	1106	8154
Gm01.VB.37	Gm01	28610000	28890000	280000	6	W	W	W	W	W	H	I	6	14	15	7	14	947	1193
Gm01.VB.38	Gm01	28890000	29070000	180000	3	W	W	W	W	W	W	I	7	13	12	6	13	24	101
Gm01.VB.39	Gm01	29070000	31910000	2840000	38	W	W	W	W	W	H	I	144	248	282	116	248	2756	1418
Gm01.VB.40	Gm01	31910000	32040000	130000	4	W	W	W	W	W	W	I	16	25	27	11	19	22	144
Gm01.VB.41	Gm01	32040000	32050000	10000	0	W	B	B	W	B	W	I	0	6	6	0	5	4	9
Gm01.VB.42	Gm01	32050000	32930000	880000	16	W	B	B	W	B	H	I	43	5679	5741	42	5678	621	529
Gm01.VB.43	Gm01	32930000	33790000	860000	8	W	W	W	W	W	H	I	32	265	280	31	278	666	542
Gm01.VB.44	Gm01	33790000	33830000	40000	0	W	W	W	W	W	H	I	0	11	11	0	9	23	20
Gm01.VB.45	Gm01	33830000	34300000	470000	7	W	B	B	W	B	B	I	16	1113	1126	11	1082	1064	697
Gm01.VB.46	Gm01	34300000	34330000	30000	0	W	B	B	B	B	B	I	17	236	231	236	213	225	167
Gm01.VB.47	Gm01	34330000	35130000	800000	11	W	W	W	G	W	G	I	231	251	260	848	256	840	787
Gm01.VB.48	Gm01	35130000	35140000	10000	1	W	W	W	W	W	W	W	0	3	3	0	3	0	3
Gm01.VB.49	Gm01	35140000	35840000	700000	15	W	B	B	W	B	W	I	57	1707	1728	39	1645	42	2234
Gm01.VB.50	Gm01	35840000	36470000	630000	21	W	B	W	W	W	W	I	38	3932	135	30	120	29	2069
Gm01.VB.51	Gm01	36470000	36540000	70000	0	W	B	W	W	W	W	I	11	91	24	10	24	10	337
Gm01.VB.52	Gm01	36540000	38690000	2150000	32	W	B	S	W	S	W	I	91	9362	7445	88	7287	83	6752
Gm01.VB.53	Gm01	38690000	38760000	70000	3	W	B	S	B	S	W	I	22	377	423	316	374	19	181
Gm01.VB.54	Gm01	38760000	39700000	940000	16	W	B	W	B	W	W	I	48	4923	173	4898	149	39	2598
Gm01.VB.55	Gm01	39700000	39790000	90000	0	W	B	S	B	S	W	I	5	122	489	121	478	5	454
Gm01.VB.56	Gm01	39790000	40100000	310000	11	W	B	W	B	W	W	I	45	582	90	590	75	34	750
Gm01.VB.57	Gm01	40100000	40250000	150000	0	W	B	S	B	S	W	I	9	76	506	77	484	5	337
Gm01.VB.58	Gm01	40250000	40370000	120000	0	W	W	S	W	S	W	I	0	14	280	8	282	0	194
Gm01.VB.59	Gm01	40370000	40400000	30000	0	W	W	S	W	S	W	I	0	7	120	3	120	0	119
Gm01.VB.60	Gm01	40400000	42130000	1730000	32	W	B	S	B	S	W	I	71	3711	5446	3618	5358	51	4866
Gm01.VB.61	Gm01	42130000	43140000	1010000	28	W	W	S	W	S	W	I	34	74	5464	78	5236	39	3244
Gm01.VB.62	Gm01	43140000	43180000	40000	4	W	B	S	B	D	W	I	5	209	250	200	204	4	172
Gm01.VB.63	Gm01	43180000	43290000	110000	4	W	W	S	W	S	W	I	5	14	626	13	585	5	399
Gm01.VB.64	Gm01	43290000	44400000	1110000	33	W	B	B	B	B	W	I	72	7243	7390	7128	6986	71	5973
Gm01.VB.65	Gm01	44400000	44570000	170000	6	W	W	S	W	S	W	I	3	10	997	10	938	3	391
Gm01.VB.66	Gm01	44570000	44710000	140000	2	W	W	W	W	W	W	I	18	22	18	19	19	20	193
Gm01.VB.67	Gm01	44710000	44760000	50000	1	W	B	B	B	B	W	I	1	156	155	155	154	1	182
Gm01.VB.68	Gm01	44760000	45180000	420000	11	W	B	B	B	B	B	I	23	782	806	774	802	802	653
Gm01.VB.69	Gm01	45180000	45570000	390000	10	W	W	W	W	W	W	I	8	36	36	39	33	33	1368
Gm01.VB.70	Gm01	45570000	45810000	240000	11	W	B	B	B	B	B	I	35	134	138	133	130	132	1005

Gm01.VB.71	Gm01	45810000	46530000	720000	20	W	W	W	W	W	H	I	40	100	104	94	91	1583	1858
Gm01.VB.72	Gm01	46530000	46780000	250000	11	W	B	B	B	B	H	I	4	473	485	472	475	137	1239
Gm01.VB.73	Gm01	46780000	46950000	170000	8	W	W	W	W	W	W	I	20	27	29	25	33	39	613
Gm01.VB.74	Gm01	46950000	47020000	70000	2	W	W	W	W	W	W	I	41	45	46	43	34	39	214
Gm01.VB.75	Gm01	47020000	47370000	350000	15	W	B	B	B	B	H	I	40	1463	1519	1481	1406	1802	1023
Gm01.VB.76	Gm01	47370000	47560000	190000	10	W	W	W	W	W	H	I	10	30	28	28	25	467	251
Gm01.VB.77	Gm01	47560000	47710000	150000	10	W	W	W	W	W	W	I	0	19	19	19	16	3	468
Gm01.VB.78	Gm01	47710000	47760000	50000	3	W	B	B	B	D	W	I	0	43	44	42	37	0	83
Gm01.VB.79	Gm01	47760000	47910000	150000	14	W	B	W	B	W	W	I	5	854	9	850	9	8	740
Gm01.VB.80	Gm01	47910000	47980000	70000	9	W	B	W	B	W	H	I	5	393	7	377	7	274	203
Gm01.VB.81	Gm01	47980000	48270000	290000	20	W	B	W	B	W	W	I	43	1744	42	1685	34	49	747
Gm01.VB.82	Gm01	48270000	48550000	280000	21	W	B	W	B	W	H	I	1	1611	1	1486	1	801	918
Gm01.VB.83	Gm01	48550000	48880000	330000	26	W	W	W	W	W	W	I	19	31	21	32	8	14	1336
Gm01.VB.84	Gm01	48880000	49060000	180000	18	W	W	W	W	W	H	I	18	20	20	21	16	332	398
Gm01.VB.85	Gm01	49060000	49420000	360000	39	W	B	W	B	W	H	I	29	1044	32	1042	29	1301	961
Gm01.VB.86	Gm01	49420000	49590000	170000	12	W	W	W	W	W	H	I	2	15	2	15	2	1080	677
Gm01.VB.87	Gm01	49590000	49670000	80000	8	W	W	W	W	W	W	I	25	32	24	30	20	24	300
Gm01.VB.88	Gm01	49670000	49930000	260000	23	W	B	W	B	W	W	I	10	765	13	753	11	17	694
Gm01.VB.89	Gm01	49930000	50340000	410000	44	W	B	W	B	W	H	I	15	1334	23	1285	14	960	901
Gm01.VB.90	Gm01	50340000	50500000	160000	17	W	B	W	B	W	W	I	12	110	11	108	8	13	200
Gm01.VB.91	Gm01	50500000	50610000	110000	13	W	B	W	B	W	B	I	3	193	1	195	1	172	313
Gm01.VB.92	Gm01	50610000	50730000	120000	11	W	W	W	W	W	H	I	3	10	4	9	1	201	164
Gm01.VB.93	Gm01	50730000	51310000	580000	60	W	W	W	W	W	W	I	18	36	19	35	17	28	1423
Gm01.VB.94	Gm01	51310000	51500000	190000	18	W	B	W	B	W	W	I	18	647	20	642	20	17	483
Gm01.VB.95	Gm01	51500000	51610000	110000	14	W	W	W	W	W	W	I	2	18	3	16	3	5	274
Gm01.VB.96	Gm01	51610000	51940000	330000	32	W	B	W	B	W	H	I	16	1336	17	1313	21	1531	1083
Gm01.VB.97	Gm01	51940000	51970000	30000	5	W	W	W	W	W	H	I	3	3	3	3	3	82	53
Gm01.VB.98	Gm01	51970000	52100000	130000	16	W	W	W	W	W	W	I	1	13	1	13	1	4	247
Gm01.VB.99	Gm01	52100000	52150000	50000	3	W	W	W	W	W	W	I	2	17	2	17	2	5	96
Gm01.VB.100	Gm01	52150000	52200000	50000	3	W	B	W	B	W	B	I	9	154	9	159	7	140	97
Gm01.VB.101	Gm01	52200000	52530000	330000	34	W	W	W	W	W	W	I	24	34	25	34	17	25	876
Gm01.VB.102	Gm01	52530000	52850000	320000	36	W	B	W	B	W	W	I	30	989	33	980	29	41	756
Gm01.VB.103	Gm01	52850000	52910000	60000	8	W	B	W	B	W	H	I	0	256	0	248	0	190	96
Gm01.VB.104	Gm01	52910000	53570000	660000	77	W	W	W	W	W	W	I	23	56	24	49	20	22	1491
Gm01.VB.105	Gm01	53570000	53730000	160000	20	W	B	W	B	W	W	I	2	615	2	615	2	2	375
Gm01.VB.106	Gm01	53730000	54620000	890000	103	W	B	B	B	B	W	I	68	3701	3571	3646	3235	59	1782
Gm01.VB.107	Gm01	54620000	54990000	370000	57	W	W	S	W	W	W	I	7	21	872	18	23	7	666
Gm01.VB.108	Gm01	54990000	55000000	10000	1	W	B	B	B	D	W	I	0	21	21	21	14	0	19
Gm01.VB.109	Gm01	55000000	55250000	250000	32	W	W	W	W	W	W	I	6	17	18	17	15	5	525
Gm01.VB.110	Gm01	55250000	55340000	90000	13	W	W	S	W	W	W	I	13	17	395	17	17	13	188
Gm01.VB.111	Gm01	55340000	55650000	310000	39	W	B	B	B	B	W	I	9	1061	1040	1045	981	9	708
Gm01.VB.112	Gm01	55650000	55915595	265595	33	W	B	S	B	B	B	I	3	833	881	823	773	792	467
Gm02.VB.1	Gm02	0	210000	210000	18	W	B	W	B	B	W	I	16	552	16	534	532	29	265
Gm02.VB.2	Gm02	210000	510000	300000	23	W	B	W	B	B	H	I	7	1186	7	1076	1039	1086	842
Gm02.VB.3	Gm02	510000	670000	160000	13	W	W	W	W	W	H	I	13	24	13	25	18	684	386
Gm02.VB.4	Gm02	670000	720000	50000	7	W	B	W	B	B	H	I	9	269	9	271	228	200	92
Gm02.VB.5	Gm02	720000	770000	50000	6	W	W	W	W	W	H	I	2	3	2	3	3	130	111
Gm02.VB.6	Gm02	770000	1330000	560000	69	W	W	W	W	W	W	I	17	27	17	28	13	34	1248
Gm02.VB.7	Gm02	1330000	1440000	110000	11	W	B	W	B	W	H	I	12	123	20	117	19	328	229
Gm02.VB.8	Gm02	1440000	1560000	120000	16	W	W	W	W	W	H	I	10	19	19	19	19	611	371
Gm02.VB.9	Gm02	1560000	1810000	250000	20	W	W	W	W	W	W	I	22	22	32	24	27	22	330
Gm02.VB.10	Gm02	1810000	1890000	80000	9	W	W	S	W	S	W	I	2	2	306	2	269	1	207
Gm02.VB.11	Gm02	1890000	1900000	10000	2	W	W	W	W	W	W	I	0	0	5	0	4	0	9
Gm02.VB.12	Gm02	1900000	2120000	220000	18	W	W	W	W	W	W	I	21	28	39	30	33	26	351
Gm02.VB.13	Gm02	2120000	2400000	280000	24	W	B	B	B	B	B	I	18	1004	896	1003	825	851	695
Gm02.VB.14	Gm02	2400000	2560000	160000	16	W	B	S	B	D	W	I	12	450	448	413	409	19	376
Gm02.VB.15	Gm02	2560000	2870000	310000	23	W	W	S	W	S	W	I	20	35	1491	34	1276	61	720
Gm02.VB.16	Gm02	2870000	2950000	80000	6	W	B	S	B	S	B	I	5	463	462	460	418	445	230
Gm02.VB.17	Gm02	2950000	3220000	270000	21	W	B	S	B	S	W	I	24	922	983	911	867	24	448
Gm02.VB.18	Gm02	3220000	3330000	110000	12	W	W	S	W	S	W	W	4	10	483	10	431	6	5
Gm02.VB.19	Gm02	3330000	3840000	510000	46	W	B	S	B	S	W	I	40	1806	2005	1776	1780	39	1074
Gm02.VB.20	Gm02	3840000	5260000	1420000	157	W	B	S	B	S	H	I	70	3640	4604	3587	4070	3746	2343
Gm02.VB.21	Gm02	5260000	5270000	10000	0	W	B	S	G	W	G	W	5	12	20	12	3	6	0
Gm02.VB.22	Gm02	5270000	5370000	100000	8	W	W	S	W	W	W	I	0	13	296	14	4	10	173
Gm02.VB.23	Gm02	5370000	5700000	330000	33	W	B	S	B	D	H	I	14	1453	1380	1385	895	923	639
Gm02.VB.24	Gm02	5700000	5830000	130000	17	W	B	S	B	S	W	I	11	497	474	490	436	15	252
Gm02.VB.25	Gm02	5830000	5840000	10000	0	W	B	S	G	S	H	I	0	14	26	11	27	24	14
Gm02.VB.26	Gm02	5840000	5990000	150000	24	W	W	S	W	S	H	I	1	4	586	4	529	663	280
Gm02.VB.27	Gm02	5990000	6000000	10000	1	W	B	S	B	S	H	I	0	9	20	9	17	16	8
Gm02.VB.28	Gm02	6000000	6030000	30000	3	W	B	S	B	S	W	I	2	128	145	124	136	2	62
Gm02.VB.29	Gm02	6030000	6180000	150000	15	W	W	S	W	S	W	I	1	14	587	13	538	10	154
Gm02.VB.30	Gm02	6180000	6620000	440000	49	W	B	S	B	S	H	I	7	1043	1689	1035	1480	934	915
Gm02.VB.31	Gm02	6620000	6810000	190000	26	W	W	S	W	S	H	I	11	17	766	19	726	511	517
Gm02.VB.32	Gm02	6810000	7250000	440000	32	W	B	S	B	S	H	I	60	746	1619	735	1478	1583	854
Gm02.VB.33	Gm02	7250000	7350000	100000	11	W	W	S	W	S	S	I	1	6	463	7	404	408	234
Gm02.VB.34	Gm02	7350000	7380000	30000	3	W	W	S	W	S	S	I	0	8	76	8	71	72	63
Gm02.VB.35	Gm02	7380000	7870000	490000	40	W	B	W	B	W	H	I	23	685	32	668	21	1358	816
Gm02.VB.36	Gm02	7870000	8020000	150000	11	W	B	W	B	W	W	I	14	762	15	736	5	30	455

Gm02.VB.37	Gm02	8020000	8060000	40000	4	W	W	W	W	W	H	I	0	8	0	8	0	152	131
Gm02.VB.38	Gm02	8060000	8340000	280000	19	W	B	W	B	W	W	I	10	901	9	888	9	32	308
Gm02.VB.39	Gm02	8340000	8380000	40000	5	W	B	W	B	W	H	I	0	107	0	108	0	71	47
Gm02.VB.40	Gm02	8380000	8650000	270000	21	W	W	W	W	W	H	I	5	16	8	16	4	615	819
Gm02.VB.41	Gm02	8650000	8740000	90000	5	W	W	W	W	W	W	I	6	13	11	12	4	6	226
Gm02.VB.42	Gm02	8740000	10900000	2160000	136	W	B	W	B	W	W	I	212	6379	215	6308	151	163	3947
Gm02.VB.43	Gm02	10900000	11060000	160000	16	W	W	W	W	W	W	I	15	17	16	17	9	11	267
Gm02.VB.44	Gm02	11060000	11160000	100000	5	W	B	W	B	W	W	I	2	100	2	99	2	2	93
Gm02.VB.45	Gm02	11160000	11490000	330000	27	W	W	W	W	W	W	I	41	60	37	51	11	18	499
Gm02.VB.46	Gm02	11490000	11640000	150000	11	W	B	W	B	W	W	I	15	362	15	361	11	14	199
Gm02.VB.47	Gm02	11640000	11940000	300000	19	W	W	W	W	W	W	I	9	25	10	22	9	10	352
Gm02.VB.48	Gm02	11940000	12100000	160000	11	W	B	W	B	W	H	I	5	560	5	542	4	431	258
Gm02.VB.49	Gm02	12100000	12250000	150000	11	W	B	W	B	W	W	I	17	318	17	313	13	14	224
Gm02.VB.50	Gm02	12250000	13520000	1270000	62	W	W	W	W	W	W	I	169	202	161	200	117	127	2148
Gm02.VB.51	Gm02	13520000	13560000	40000	4	W	B	W	B	W	W	I	11	123	15	123	11	9	149
Gm02.VB.52	Gm02	13560000	13720000	160000	17	W	W	W	W	W	W	I	8	14	8	14	7	10	366
Gm02.VB.53	Gm02	13720000	13940000	220000	18	W	B	W	B	W	H	I	15	592	16	544	6	288	413
Gm02.VB.54	Gm02	13940000	14270000	330000	22	W	W	W	W	W	W	I	26	37	25	38	21	137	666
Gm02.VB.55	Gm02	14270000	14870000	600000	58	W	B	W	B	W	B	I	28	898	30	881	19	763	851
Gm02.VB.56	Gm02	14870000	15310000	440000	45	W	W	W	W	W	W	I	29	52	31	52	35	63	705
Gm02.VB.57	Gm02	15310000	15480000	170000	19	W	B	W	B	W	B	I	21	356	20	364	11	357	238
Gm02.VB.58	Gm02	15480000	15580000	100000	9	W	W	W	W	W	W	I	25	33	30	26	15	19	218
Gm02.VB.59	Gm02	15580000	15610000	30000	1	W	B	W	B	B	B	W	0	101	0	99	99	96	8
Gm02.VB.60	Gm02	15610000	15710000	100000	8	W	W	W	W	W	W	I	4	11	4	10	7	8	62
Gm02.VB.61	Gm02	15710000	22520000	6810000	58	W	B	W	B	B	B	I	557	16532	577	16323	16784	16832	3404
Gm02.VB.62	Gm02	22520000	22940000	420000	1	W	B	S	G	D	W	W	137	1273	1251	1161	1270	128	159
Gm02.VB.63	Gm02	22940000	23030000	90000	0	W	B	S	G	D	W	W	38	128	146	124	133	37	44
Gm02.VB.64	Gm02	23030000	24340000	1310000	5	W	B	W	B	B	B	I	30	2357	38	2318	2367	2380	587
Gm02.VB.65	Gm02	24340000	24450000	110000	0	W	W	W	W	W	W	I	2	13	1	13	10	18	49
Gm02.VB.66	Gm02	24450000	24460000	10000	0	W	B	W	G	D	H	W	0	10	0	7	11	9	3
Gm02.VB.67	Gm02	24460000	24500000	40000	0	W	B	W	W	W	W	I	0	20	0	1	5	3	36
Gm02.VB.68	Gm02	24500000	24750000	250000	0	W	W	W	W	W	W	I	6	20	5	28	18	7	129
Gm02.VB.69	Gm02	24750000	24780000	30000	0	W	W	W	W	W	W	I	0	7	0	0	0	0	20
Gm02.VB.70	Gm02	24780000	25940000	1160000	6	W	B	W	B	B	B	I	38	2344	37	2306	2384	2394	606
Gm02.VB.71	Gm02	25940000	26080000	140000	8	W	W	W	W	W	H	I	36	36	34	32	43	280	413
Gm02.VB.72	Gm02	26080000	27560000	1480000	15	W	B	W	B	W	H	I	40	2455	36	2401	73	2180	1317
Gm02.VB.73	Gm02	27560000	27770000	210000	7	W	W	W	W	W	W	I	5	57	5	53	49	50	553
Gm02.VB.74	Gm02	27770000	31330000	3560000	58	W	B	W	B	B	B	I	262	5344	250	5339	5397	5442	3130
Gm02.VB.75	Gm02	31330000	34300000	2970000	43	W	W	W	W	W	W	I	124	192	133	199	179	174	2129
Gm02.VB.76	Gm02	34300000	34320000	20000	1	W	B	W	B	B	B	B	0	96	0	95	97	96	83
Gm02.VB.77	Gm02	34320000	41970000	7650000	153	W	W	W	W	W	W	I	261	362	275	361	384	366	18266
Gm02.VB.78	Gm02	41970000	42210000	240000	26	W	B	W	B	B	B	I	3	440	2	429	425	444	517
Gm02.VB.79	Gm02	42210000	42310000	100000	8	W	W	W	W	W	W	I	0	6	0	5	5	5	142
Gm02.VB.80	Gm02	42310000	42360000	50000	7	W	B	W	B	D	H	I	3	103	3	103	39	25	49
Gm02.VB.81	Gm02	42360000	42600000	240000	16	W	B	W	B	W	W	I	26	1063	24	1061	65	45	676
Gm02.VB.82	Gm02	42600000	43140000	540000	38	W	B	W	B	D	H	I	55	3403	53	3320	1190	830	1535
Gm02.VB.83	Gm02	43140000	43220000	80000	9	W	B	W	B	W	W	I	11	246	12	248	11	10	113
Gm02.VB.84	Gm02	43220000	43610000	390000	22	W	W	W	W	W	W	I	17	37	17	38	12	29	282
Gm02.VB.85	Gm02	43610000	43630000	20000	1	W	B	W	B	W	W	I	0	10	0	10	0	0	20
Gm02.VB.86	Gm02	43630000	43930000	300000	21	W	B	W	B	W	B	I	32	763	38	765	26	681	1047
Gm02.VB.87	Gm02	43930000	44060000	130000	16	W	W	W	W	W	H	I	4	7	3	7	6	88	256
Gm02.VB.88	Gm02	44060000	44160000	100000	7	W	B	W	B	W	H	I	0	402	0	401	0	141	279
Gm02.VB.89	Gm02	44160000	44240000	80000	7	W	W	W	W	W	H	I	0	3	0	4	1	50	170
Gm02.VB.90	Gm02	44240000	44260000	20000	4	W	W	W	W	W	W	I	1	6	1	5	1	3	45
Gm02.VB.91	Gm02	44260000	44420000	160000	19	W	B	W	B	W	W	I	11	836	12	837	12	14	513
Gm02.VB.92	Gm02	44420000	44530000	110000	11	W	B	W	B	W	H	I	3	433	3	429	10	308	429
Gm02.VB.93	Gm02	44530000	44540000	10000	1	W	W	W	W	W	H	I	0	0	0	0	1	8	34
Gm02.VB.94	Gm02	44540000	44620000	80000	3	W	W	W	W	W	W	I	2	10	2	13	2	2	332
Gm02.VB.95	Gm02	44620000	44770000	150000	11	W	W	W	W	W	W	I	2	16	2	16	6	6	354
Gm02.VB.96	Gm02	44770000	44850000	80000	7	W	B	W	B	D	D	I	0	355	0	337	186	166	230
Gm02.VB.97	Gm02	44850000	45160000	310000	28	W	W	W	W	W	W	I	7	29	7	30	22	21	784
Gm02.VB.98	Gm02	45160000	46380000	1220000	104	W	B	W	B	B	B	I	100	4501	102	4483	4088	4175	3282
Gm02.VB.99	Gm02	46380000	46850000	470000	46	W	W	W	W	W	W	I	21	33	30	32	25	28	1322
Gm02.VB.100	Gm02	46850000	47060000	210000	20	W	W	S	W	W	W	I	3	8	798	8	7	7	423
Gm02.VB.101	Gm02	47060000	47140000	80000	5	W	B	S	B	B	B	I	5	208	245	200	198	190	135
Gm02.VB.102	Gm02	47140000	47250000	110000	13	W	W	W	W	W	W	I	2	8	8	8	6	5	138
Gm02.VB.103	Gm02	47250000	47300000	50000	5	W	W	S	W	W	W	I	1	5	197	5	4	5	91
Gm02.VB.104	Gm02	47300000	47690000	390000	32	W	B	S	B	B	B	I	26	1381	1096	1325	1264	1292	840
Gm02.VB.105	Gm02	47690000	48130000	440000	52	W	B	W	B	B	B	I	22	1085	30	1068	944	983	1163
Gm02.VB.106	Gm02	48130000	48280000	150000	15	W	B	S	B	B	B	I	6	349	294	349	330	338	183
Gm02.VB.107	Gm02	48280000	48400000	120000	11	W	B	W	B	B	B	I	0	331	3	329	313	324	235
Gm02.VB.108	Gm02	48400000	48580000	180000	21	W	W	W	W	W	W	I	6	15	11	16	15	15	384
Gm02.VB.109	Gm02	48580000	48950000	370000	41	W	B	B	B	B	B	I	10	1127	1036	1104	1010	1069	575
Gm02.VB.110	Gm02	48950000	49050000	100000	11	W	W	W	W	W	W	I	7	9	10	9	6	6	233
Gm02.VB.111	Gm02	49050000	49070000	20000	3	W	B	S	B	D	D	I	1	44	58	46	36	35	46
Gm02.VB.112	Gm02	49070000	49150000	80000	9	W	W	S	W	W	W	I	2	4	276	4	3	4	332
Gm02.VB.113	Gm02	49150000	49270000	120000	15	W	W	W	W	W	W	I	7	11	9	12	12	12	211
Gm02.VB.114	Gm02	49270000	49310000	40000	2	W	B	B	B	B	B	I	13	62	57	59	46	46	25

Gm02.VB.115	Gm02	49310000	49600000	290000	29	W	W	W	W	W	W	I	23	27	39	26	22	24	930
Gm02.VB.116	Gm02	49600000	49790000	190000	25	W	W	S	W	W	W	I	20	18	511	19	15	13	456
Gm02.VB.117	Gm02	49790000	49950000	160000	19	W	W	W	W	W	W	I	30	33	36	31	32	31	320
Gm02.VB.118	Gm02	49950000	49980000	30000	4	W	B	W	B	B	B	I	0	87	0	85	81	82	82
Gm02.VB.119	Gm02	49980000	50000000	20000	2	W	B	S	B	B	B	I	0	128	98	127	123	124	66
Gm02.VB.120	Gm02	50000000	50140000	140000	18	W	B	W	B	B	B	I	25	177	33	177	171	175	107
Gm02.VB.121	Gm02	50140000	50750000	610000	82	W	B	S	B	B	B	I	23	2042	1883	2025	1884	1922	1284
Gm02.VB.122	Gm02	50750000	50930000	180000	27	W	W	W	W	W	W	I	11	19	15	19	12	11	440
Gm02.VB.123	Gm02	50930000	51656713	726713	81	W	B	B	B	B	B	I	30	2155	2057	2104	1970	2024	1467
Gm03.VB.1	Gm03	0	160000	160000	12	W	W	W	W	W	H	I	11	20	27	20	22	98	125
Gm03.VB.2	Gm03	160000	200000	40000	4	W	W	S	W	W	H	I	0	0	263	0	0	22	241
Gm03.VB.3	Gm03	200000	600000	400000	44	W	B	S	B	B	H	I	24	1219	1312	1187	1107	1121	1199
Gm03.VB.4	Gm03	600000	740000	140000	11	W	B	W	B	B	W	I	19	861	23	820	718	25	380
Gm03.VB.5	Gm03	740000	990000	250000	22	W	B	W	B	B	H	I	23	567	23	558	526	316	695
Gm03.VB.6	Gm03	990000	1530000	540000	37	W	B	W	B	B	W	I	35	1796	35	1728	1683	46	2068
Gm03.VB.7	Gm03	1530000	2000000	470000	37	W	B	W	B	B	H	I	43	2233	43	2205	2084	1871	1337
Gm03.VB.8	Gm03	2000000	2240000	240000	16	W	B	S	B	B	H	I	65	1353	1211	1298	1196	1102	762
Gm03.VB.9	Gm03	2240000	2910000	670000	38	W	B	W	B	B	H	I	61	3750	94	3606	3395	2827	2211
Gm03.VB.10	Gm03	2910000	3600000	690000	45	W	B	S	B	B	B	I	80	4620	3862	4535	4285	4216	2894
Gm03.VB.11	Gm03	3600000	3910000	310000	16	W	B	W	B	B	B	I	64	1947	72	1948	1788	1814	1029
Gm03.VB.12	Gm03	3910000	6260000	2350000	105	W	B	S	B	D	H	I	1088	12495	12869	12162	12472	11445	9002
Gm03.VB.13	Gm03	6260000	6340000	80000	0	W	W	S	W	S	H	I	4	7	104	7	93	88	191
Gm03.VB.14	Gm03	6340000	6360000	20000	0	W	W	W	W	W	W	W	0	0	0	0	0	0	0
Gm03.VB.15	Gm03	6360000	6370000	10000	0	W	W	W	G	W	W	W	0	1	0	6	0	0	1
Gm03.VB.16	Gm03	6370000	6380000	10000	0	W	B	W	G	D	H	I	0	20	2	13	5	38	23
Gm03.VB.17	Gm03	6380000	6510000	130000	5	W	B	S	W	S	H	I	93	115	9	101	8	154	121
Gm03.VB.18	Gm03	6510000	6520000	10000	0	W	W	S	G	D	H	I	13	15	1	10	9	11	9
Gm03.VB.19	Gm03	6520000	7240000	720000	28	W	W	S	W	S	H	I	1114	1096	2111	1079	1993	2675	3123
Gm03.VB.20	Gm03	7240000	7250000	10000	0	W	W	S	W	D	W	I	0	0	5	0	11	0	25
Gm03.VB.21	Gm03	7250000	7260000	10000	0	W	W	S	W	S	S	I	9	9	1	9	0	2	172
Gm03.VB.22	Gm03	7260000	7560000	300000	3	W	W	W	W	W	W	I	13	18	62	18	35	10	1374
Gm03.VB.23	Gm03	7560000	7580000	20000	0	W	B	B	B	B	B	I	9	7	1	2	0	0	78
Gm03.VB.24	Gm03	7580000	7600000	20000	1	W	W	W	W	D	W	I	1	1	3	1	9	0	122
Gm03.VB.25	Gm03	7600000	7610000	10000	1	W	W	S	W	D	H	I	2	2	98	2	62	15	47
Gm03.VB.26	Gm03	7610000	7660000	50000	2	W	W	S	W	S	H	I	66	65	79	65	70	169	129
Gm03.VB.27	Gm03	7660000	7780000	120000	3	W	W	W	W	W	H	I	41	51	12	47	19	347	171
Gm03.VB.28	Gm03	7780000	8840000	1060000	32	W	W	S	W	S	H	I	1203	1229	2899	1207	2752	2767	3526
Gm03.VB.29	Gm03	8840000	9070000	230000	8	W	W	S	W	D	H	I	36	41	235	40	203	407	372
Gm03.VB.30	Gm03	9070000	9280000	210000	4	W	W	S	W	S	H	I	363	355	603	343	598	470	422
Gm03.VB.31	Gm03	9280000	9350000	70000	0	W	W	S	W	D	W	W	21	22	82	16	95	38	27
Gm03.VB.32	Gm03	9350000	9380000	30000	0	W	W	W	W	W	W	W	0	0	0	0	0	0	0
Gm03.VB.33	Gm03	9380000	9450000	70000	0	W	B	S	G	S	S	S	40	43	12	44	10	23	23
Gm03.VB.34	Gm03	9450000	12370000	2920000	25	W	W	S	W	S	H	I	2102	2036	3357	2064	3380	4117	8828
Gm03.VB.35	Gm03	12370000	12390000	20000	0	W	W	W	W	W	H	W	2	6	0	10	0	13	0
Gm03.VB.36	Gm03	12390000	12520000	130000	1	W	W	W	W	W	H	I	8	19	44	10	48	124	294
Gm03.VB.37	Gm03	12520000	12620000	100000	2	W	W	S	W	W	W	I	9	13	49	15	41	12	524
Gm03.VB.38	Gm03	12620000	13170000	550000	4	W	W	S	W	S	H	I	248	229	369	229	374	678	2072
Gm03.VB.39	Gm03	13170000	13220000	50000	0	W	W	W	W	W	W	W	0	0	9	0	11	11	7
Gm03.VB.40	Gm03	13220000	13260000	40000	0	W	W	W	W	W	H	I	4	4	0	12	3	28	24
Gm03.VB.41	Gm03	13260000	13360000	100000	0	W	W	W	W	W	W	W	2	3	0	3	0	6	9
Gm03.VB.42	Gm03	13360000	13410000	50000	0	W	W	W	W	W	H	I	5	3	12	5	3	48	23
Gm03.VB.43	Gm03	13410000	13420000	10000	0	W	B	S	W	D	H	W	0	5	10	0	12	14	2
Gm03.VB.44	Gm03	13420000	13430000	10000	0	W	W	S	W	S	H	I	29	32	3	32	2	29	26
Gm03.VB.45	Gm03	13430000	13540000	110000	5	W	W	W	W	W	W	W	9	16	6	8	7	39	25
Gm03.VB.46	Gm03	13540000	14000000	460000	1	W	W	S	W	S	H	I	258	245	339	253	340	731	1965
Gm03.VB.47	Gm03	14000000	14030000	30000	0	W	W	S	W	D	H	I	7	6	38	5	25	31	70
Gm03.VB.48	Gm03	14030000	14050000	20000	0	W	W	W	W	W	H	I	4	7	2	7	0	36	37
Gm03.VB.49	Gm03	14050000	14170000	120000	1	W	W	W	W	W	W	W	1	1	3	3	5	3	5
Gm03.VB.50	Gm03	14170000	14180000	10000	0	W	W	W	W	D	W	W	1	1	3	1	5	2	1
Gm03.VB.51	Gm03	14180000	14200000	20000	0	W	W	S	W	D	W	W	4	4	14	3	21	11	5
Gm03.VB.52	Gm03	14200000	14500000	300000	6	W	W	S	W	S	H	I	275	275	481	265	473	419	344
Gm03.VB.53	Gm03	14500000	14510000	10000	0	W	W	W	W	D	H	W	0	0	0	0	5	23	0
Gm03.VB.54	Gm03	14510000	14540000	30000	0	W	W	W	W	W	H	I	0	0	6	2	0	49	17
Gm03.VB.55	Gm03	14540000	14660000	120000	1	W	W	W	W	W	H	I	11	9	9	12	16	145	72
Gm03.VB.56	Gm03	14660000	15620000	960000	14	W	W	S	W	S	H	I	941	905	1527	908	1543	1393	2141
Gm03.VB.57	Gm03	15620000	16150000	530000	1	W	W	S	W	S	H	I	120	119	323	112	316	786	1026
Gm03.VB.58	Gm03	16150000	18620000	2470000	28	W	W	W	W	W	H	I	114	188	186	179	178	2392	3653
Gm03.VB.59	Gm03	18620000	19220000	600000	19	W	W	W	W	W	W	I	30	45	46	47	40	41	830
Gm03.VB.60	Gm03	19220000	23420000	4200000	69	W	B	S	B	S	H	I	4094	6866	7435	6852	7408	8911	8651
Gm03.VB.61	Gm03	23420000	23660000	240000	1	W	W	W	W	W	W	W	51	48	43	44	36	60	116
Gm03.VB.62	Gm03	23660000	24010000	350000	5	W	W	W	W	W	H	I	10	33	16	33	16	162	246
Gm03.VB.63	Gm03	24010000	24160000	150000	1	W	W	W	W	W	W	I	2	9	6	9	4	7	73
Gm03.VB.64	Gm03	24160000	24450000	290000	4	W	W	W	W	W	H	I	5	20	17	19	20	173	229
Gm03.VB.65	Gm03	24450000	24620000	170000	1	W	W	W	W	W	W	I	5	18	8	7	14	37	91
Gm03.VB.66	Gm03	24620000	28120000	3500000	39	W	W	W	W	W	H	I	143	240	227	227	259	2595	3334
Gm03.VB.67	Gm03	28120000	28220000	100000	3	W	W	W	W	W	W	I	5	6	6	7	5	16	76
Gm03.VB.68	Gm03	28220000	28440000	220000	3	W	W	W	W	W	H	I	9	13	12	15	12	704	1851
Gm03.VB.69	Gm03	28440000	28720000	280000	10	W	W	W	W	W	W	I	5	12	10	10	10	45	843

Gm03.VB.70	Gm03	28720000	29670000	950000	20	W	W	W	W	W	H	I	66	106	91	95	74	550	480
Gm03.VB.71	Gm03	29670000	29780000	110000	0	W	W	W	W	W	W	I	0	8	4	7	4	4	375
Gm03.VB.72	Gm03	29780000	30260000	480000	11	W	W	W	W	W	H	I	18	40	29	35	21	343	714
Gm03.VB.73	Gm03	30260000	30630000	370000	16	W	W	W	W	W	W	I	46	524	349	520	334	282	703
Gm03.VB.74	Gm03	30630000	30820000	190000	9	W	W	W	W	W	H	I	32	532	326	525	321	302	663
Gm03.VB.75	Gm03	30820000	31070000	250000	10	W	W	W	W	W	W	W	43	706	350	707	343	264	319
Gm03.VB.76	Gm03	31070000	31360000	290000	4	W	W	W	W	W	H	I	53	474	455	481	466	776	1057
Gm03.VB.77	Gm03	31360000	31550000	190000	5	W	W	W	W	W	W	I	16	175	173	167	158	169	628
Gm03.VB.78	Gm03	31550000	31750000	200000	5	W	B	B	B	W	W	I	63	402	391	377	338	352	1130
Gm03.VB.79	Gm03	31750000	31760000	10000	0	W	B	W	B	B	B	I	5	33	32	34	35	36	74
Gm03.VB.80	Gm03	31760000	33050000	1290000	45	W	W	W	W	W	W	I	111	170	142	163	127	137	2928
Gm03.VB.81	Gm03	33050000	33410000	360000	5	W	B	W	B	W	W	I	76	2679	83	2642	67	78	801
Gm03.VB.82	Gm03	33410000	33960000	550000	16	W	W	W	W	W	W	I	21	118	39	98	35	43	2248
Gm03.VB.83	Gm03	33960000	34170000	210000	15	W	B	W	W	W	W	I	17	959	29	30	23	27	329
Gm03.VB.84	Gm03	34170000	34700000	530000	23	W	W	W	W	W	W	I	92	120	117	107	106	123	2435
Gm03.VB.85	Gm03	34700000	34790000	90000	3	W	B	W	W	W	W	I	27	462	29	24	26	29	250
Gm03.VB.86	Gm03	34790000	34830000	40000	2	W	B	W	W	W	H	I	0	440	2	2	2	94	91
Gm03.VB.87	Gm03	34830000	34850000	20000	1	W	B	W	W	W	W	I	2	125	3	3	3	3	80
Gm03.VB.88	Gm03	34850000	34970000	120000	4	W	W	W	W	W	W	I	13	17	18	12	6	13	459
Gm03.VB.89	Gm03	34970000	34980000	10000	1	W	B	W	W	W	W	I	1	23	0	0	0	2	12
Gm03.VB.90	Gm03	34980000	35220000	240000	13	W	B	W	W	W	H	I	34	929	48	40	37	443	583
Gm03.VB.91	Gm03	35220000	35230000	10000	1	W	W	W	W	W	H	I	0	2	0	0	0	6	23
Gm03.VB.92	Gm03	35230000	35310000	80000	7	W	W	W	W	W	W	I	4	5	12	12	12	7	359
Gm03.VB.93	Gm03	35310000	35460000	150000	6	W	W	S	G	D	W	I	8	113	434	425	436	104	395
Gm03.VB.94	Gm03	35460000	35510000	50000	3	W	W	W	W	W	W	I	1	6	55	54	54	7	168
Gm03.VB.95	Gm03	35510000	35540000	30000	2	W	W	W	W	W	H	I	10	11	11	11	11	71	89
Gm03.VB.96	Gm03	35540000	35770000	230000	15	W	B	W	W	W	H	I	11	582	17	18	16	483	722
Gm03.VB.97	Gm03	35770000	36020000	250000	10	W	B	B	B	W	B	I	52	1818	1634	1578	62	1640	1172
Gm03.VB.98	Gm03	36020000	36110000	90000	6	W	B	W	W	W	W	I	2	95	27	26	62	62	196
Gm03.VB.99	Gm03	36110000	36250000	140000	7	W	W	W	W	W	W	I	32	41	40	41	26	31	513
Gm03.VB.100	Gm03	36250000	36280000	30000	4	W	W	S	S	W	H	I	2	4	159	158	6	89	88
Gm03.VB.101	Gm03	36280000	36560000	280000	19	W	B	S	S	W	S	I	21	767	799	778	19	678	1051
Gm03.VB.102	Gm03	36560000	36640000	80000	3	W	B	B	B	B	H	I	24	702	686	664	626	269	163
Gm03.VB.103	Gm03	36640000	36940000	300000	24	W	B	B	B	B	W	I	27	433	444	431	393	27	684
Gm03.VB.104	Gm03	36940000	37090000	150000	9	W	B	S	S	S	H	I	15	660	441	437	386	308	357
Gm03.VB.105	Gm03	37090000	37240000	150000	9	W	B	W	W	W	H	I	3	879	9	11	10	466	350
Gm03.VB.106	Gm03	37240000	37500000	260000	16	W	B	S	S	S	H	I	10	1319	1218	1197	1121	822	829
Gm03.VB.107	Gm03	37500000	37600000	100000	11	W	W	S	S	S	W	I	2	18	91	93	93	4	108
Gm03.VB.108	Gm03	37600000	37740000	140000	8	W	W	S	S	S	W	I	17	26	313	309	281	10	232
Gm03.VB.109	Gm03	37740000	37750000	10000	0	W	B	S	S	S	W	I	0	104	18	18	18	0	43
Gm03.VB.110	Gm03	37750000	37810000	60000	4	W	B	S	S	S	B	I	3	84	320	322	267	73	234
Gm03.VB.111	Gm03	37810000	37910000	100000	14	W	W	S	S	D	W	I	3	11	355	325	285	24	163
Gm03.VB.112	Gm03	37910000	37970000	60000	5	W	B	S	S	S	H	I	0	148	67	67	64	178	132
Gm03.VB.113	Gm03	37970000	38130000	160000	16	W	B	S	S	S	W	I	0	647	869	843	799	3	456
Gm03.VB.114	Gm03	38130000	38160000	30000	4	W	B	B	B	B	H	I	0	106	137	132	125	80	110
Gm03.VB.115	Gm03	38160000	38250000	90000	8	W	W	S	S	S	H	I	13	9	322	317	292	143	138
Gm03.VB.116	Gm03	38250000	38360000	110000	10	W	W	W	W	W	H	I	15	16	18	20	7	153	128
Gm03.VB.117	Gm03	38360000	38380000	20000	1	W	W	S	S	W	W	I	5	5	14	14	12	5	49
Gm03.VB.118	Gm03	38380000	38450000	70000	6	W	W	S	S	S	W	I	7	9	305	296	254	11	184
Gm03.VB.119	Gm03	38450000	38520000	70000	10	W	B	W	W	W	B	I	0	225	4	4	4	224	229
Gm03.VB.120	Gm03	38520000	38680000	160000	14	W	W	W	W	W	W	I	9	22	14	14	18	21	417
Gm03.VB.121	Gm03	38680000	38770000	90000	8	W	B	W	W	W	B	I	16	246	22	20	20	232	268
Gm03.VB.122	Gm03	38770000	39000000	230000	15	W	B	W	W	W	W	I	9	707	13	12	12	14	522
Gm03.VB.123	Gm03	39000000	39340000	340000	28	W	W	W	W	W	W	I	13	28	22	22	19	22	812
Gm03.VB.124	Gm03	39340000	39490000	150000	16	W	W	S	S	S	H	I	19	26	353	348	320	407	513
Gm03.VB.125	Gm03	39490000	39760000	270000	25	W	B	B	B	B	H	I	34	756	751	734	683	418	640
Gm03.VB.126	Gm03	39760000	40080000	320000	30	W	B	W	W	W	W	I	19	1889	31	29	26	35	791
Gm03.VB.127	Gm03	40080000	40870000	790000	80	W	B	W	W	W	H	I	58	3906	67	59	46	2317	2166
Gm03.VB.128	Gm03	40870000	41040000	170000	24	W	B	S	S	S	H	I	0	808	738	726	688	540	146
Gm03.VB.129	Gm03	41040000	41350000	310000	33	W	B	W	W	W	H	I	18	1572	26	22	17	1081	715
Gm03.VB.130	Gm03	41350000	41500000	150000	13	W	W	W	W	W	W	I	6	10	7	5	7	8	293
Gm03.VB.131	Gm03	41500000	41530000	30000	3	W	W	W	W	W	W	I	0	5	0	0	0	5	30
Gm03.VB.132	Gm03	41530000	41680000	150000	11	W	B	W	W	W	H	I	21	365	24	22	20	427	286
Gm03.VB.133	Gm03	41680000	41730000	50000	5	W	B	W	W	W	W	I	17	79	12	12	5	8	40
Gm03.VB.134	Gm03	41730000	41870000	140000	16	W	W	W	W	W	W	I	9	9	9	8	7	8	215
Gm03.VB.135	Gm03	41870000	42130000	260000	32	W	B	W	W	W	W	I	1	611	2	2	3	13	623
Gm03.VB.136	Gm03	42130000	42320000	190000	16	W	B	W	W	W	H	I	6	614	9	9	9	346	415
Gm03.VB.137	Gm03	42320000	42880000	560000	68	W	W	W	W	W	W	I	31	67	35	32	30	63	1123
Gm03.VB.138	Gm03	42880000	43020000	140000	21	W	W	W	W	W	H	I	1	3	2	1	3	579	328
Gm03.VB.139	Gm03	43020000	43310000	290000	28	W	W	W	W	W	W	I	7	9	12	10	12	15	553
Gm03.VB.140	Gm03	43310000	43360000	50000	6	W	W	S	S	S	W	I	0	1	75	77	74	1	103
Gm03.VB.141	Gm03	43360000	43470000	110000	14	W	W	W	W	W	W	I	0	1	0	0	0	3	161
Gm03.VB.142	Gm03	43470000	43620000	150000	19	W	W	W	W	W	H	I	14	14	16	17	16	534	256
Gm03.VB.143	Gm03	43620000	44020000	400000	55	W	W	W	W	W	W	I	6	12	9	8	5	22	961
Gm03.VB.144	Gm03	44020000	44110000	90000	9	W	W	W	W	W	H	I	6	8	6	6	6	173	202
Gm03.VB.145	Gm03	44110000	44400000	290000	38	W	W	W	W	W	W	I	7	13	14	12	9	13	280
Gm03.VB.146	Gm03	44400000	44480000	80000	10	W	W	S	S	S	S	I	21	21	256	244	218	229	136
Gm03.VB.147	Gm03	44480000	44560000	80000	8	W	W	W	W	W	W	I	3	6	10	11	10	10	130

Gm03.VB.148	Gm03	44560000	44700000	140000	18	W	B	W	W	W	W	I	5	159	9	8	6	5	324
Gm03.VB.149	Gm03	44700000	44710000	10000	2	W	B	S	S	D	D	I	0	37	31	30	22	22	23
Gm03.VB.150	Gm03	44710000	44810000	100000	8	W	B	S	S	S	W	I	5	421	322	327	270	6	214
Gm03.VB.151	Gm03	44810000	45010000	200000	21	W	B	B	B	B	H	I	24	719	744	731	698	195	352
Gm03.VB.152	Gm03	45010000	45130000	120000	11	W	B	B	B	B	W	I	15	459	467	449	408	18	71
Gm03.VB.153	Gm03	45130000	45230000	100000	16	W	B	B	B	B	H	I	13	376	386	378	349	183	163
Gm03.VB.154	Gm03	45230000	45350000	120000	15	W	B	B	B	B	W	I	0	392	396	384	371	9	229
Gm03.VB.155	Gm03	45350000	45370000	20000	2	W	B	B	B	B	H	I	0	50	52	52	42	40	39
Gm03.VB.156	Gm03	45370000	46050000	680000	90	W	W	W	W	W	H	I	40	91	49	51	82	1231	1496
Gm03.VB.157	Gm03	46050000	46430000	380000	45	W	B	B	B	B	H	I	35	1361	1360	1339	1244	858	648
Gm03.VB.158	Gm03	46430000	47020000	590000	69	W	W	W	W	W	W	I	49	71	66	53	57	45	1309
Gm03.VB.159	Gm03	47020000	47370000	350000	41	W	B	W	B	B	B	I	59	1292	68	1281	1183	1202	863
Gm03.VB.160	Gm03	47370000	47780000	410000	49	W	W	S	W	W	W	I	32	39	882	40	32	41	799
Gm03.VB.161	Gm03	47780000	47781076	1076	0	W	B	W	G	D	W	I	0	2	0	1	1	0	1
Gm04.VB.1	Gm04	0	230000	230000	24	W	B	W	W	W	W	I	15	507	39	33	20	21	391
Gm04.VB.2	Gm04	230000	270000	40000	5	W	W	W	W	W	W	I	0	2	3	3	2	3	121
Gm04.VB.3	Gm04	270000	520000	250000	35	W	W	S	S	S	S	I	7	31	1053	1030	913	962	647
Gm04.VB.4	Gm04	520000	600000	80000	9	W	W	W	W	W	W	I	7	25	15	24	13	12	134
Gm04.VB.5	Gm04	600000	650000	50000	5	W	W	W	W	W	W	I	0	8	8	8	8	8	28
Gm04.VB.6	Gm04	650000	680000	30000	1	W	W	S	W	S	S	W	0	2	57	2	55	56	8
Gm04.VB.7	Gm04	680000	880000	200000	25	W	W	W	W	W	W	I	1	20	10	22	10	13	286
Gm04.VB.8	Gm04	880000	910000	30000	2	W	W	W	W	W	W	I	3	4	12	1	10	13	33
Gm04.VB.9	Gm04	910000	920000	10000	0	W	W	S	W	D	D	I	0	0	7	0	5	5	38
Gm04.VB.10	Gm04	920000	1120000	200000	24	W	B	S	B	S	S	I	7	663	665	654	610	615	236
Gm04.VB.11	Gm04	1120000	1160000	40000	6	W	W	S	W	S	S	I	1	3	191	3	171	176	70
Gm04.VB.12	Gm04	1160000	1520000	360000	37	W	W	W	W	W	W	I	7	12	24	13	18	17	495
Gm04.VB.13	Gm04	1520000	1610000	90000	12	W	W	S	W	S	S	I	0	1	394	1	341	358	184
Gm04.VB.14	Gm04	1610000	1620000	10000	0	W	W	S	W	W	S	I	0	3	5	3	4	5	5
Gm04.VB.15	Gm04	1620000	1950000	330000	38	W	W	W	W	W	W	I	25	65	55	62	41	44	664
Gm04.VB.16	Gm04	1950000	2140000	190000	16	W	W	S	W	S	S	I	12	23	296	23	277	282	287
Gm04.VB.17	Gm04	2140000	2700000	560000	65	W	B	B	B	B	B	I	32	1864	1870	1831	1689	1684	1090
Gm04.VB.18	Gm04	2700000	3830000	1130000	126	W	W	W	W	W	H	I	58	59	66	59	57	3740	2399
Gm04.VB.19	Gm04	3830000	4710000	880000	86	W	W	W	W	W	W	I	42	47	52	48	42	36	1868
Gm04.VB.20	Gm04	4710000	4820000	110000	12	W	W	S	W	S	W	I	12	13	154	12	147	12	184
Gm04.VB.21	Gm04	4820000	5000000	180000	18	W	W	W	W	W	W	I	13	15	22	14	21	15	335
Gm04.VB.22	Gm04	5000000	5040000	40000	3	W	W	S	W	S	W	I	0	0	31	0	29	0	30
Gm04.VB.23	Gm04	5040000	5130000	90000	7	W	W	W	W	W	W	I	0	3	0	3	0	0	99
Gm04.VB.24	Gm04	5130000	5270000	140000	12	W	B	W	B	W	W	I	31	393	33	378	35	30	213
Gm04.VB.25	Gm04	5270000	5280000	10000	1	W	B	S	B	S	W	I	0	34	20	34	20	0	28
Gm04.VB.26	Gm04	5280000	5290000	10000	1	W	W	S	W	S	W	I	0	0	85	0	79	0	26
Gm04.VB.27	Gm04	5290000	5740000	450000	51	W	W	W	W	W	W	I	31	28	60	28	60	29	783
Gm04.VB.28	Gm04	5740000	5760000	20000	1	W	W	S	W	S	W	I	0	0	62	0	54	0	52
Gm04.VB.29	Gm04	5760000	6030000	270000	23	W	W	W	W	W	W	I	39	50	47	40	36	31	482
Gm04.VB.30	Gm04	6030000	6040000	10000	2	W	W	S	W	S	W	I	0	0	12	0	12	0	7
Gm04.VB.31	Gm04	6040000	6140000	100000	11	W	B	B	B	B	B	I	1	322	349	326	316	300	249
Gm04.VB.32	Gm04	6140000	6240000	100000	8	W	W	S	W	S	W	I	12	13	551	10	484	11	303
Gm04.VB.33	Gm04	6240000	6350000	110000	9	W	B	S	B	S	B	I	9	540	94	535	86	508	288
Gm04.VB.34	Gm04	6350000	6600000	250000	26	W	W	S	W	S	W	I	11	19	243	17	210	17	293
Gm04.VB.35	Gm04	6600000	6650000	50000	3	W	B	S	B	S	B	I	20	192	284	205	257	153	140
Gm04.VB.36	Gm04	6650000	6740000	90000	11	W	W	S	W	S	W	I	0	1	107	1	100	0	148
Gm04.VB.37	Gm04	6740000	6870000	130000	10	W	W	W	W	W	W	I	37	39	77	39	45	41	322
Gm04.VB.38	Gm04	6870000	7320000	450000	37	W	B	S	B	D	B	I	26	2430	2506	2413	1822	2050	1664
Gm04.VB.39	Gm04	7320000	7530000	210000	17	W	W	W	W	W	W	I	9	22	21	24	19	10	503
Gm04.VB.40	Gm04	7530000	7550000	20000	1	W	B	B	B	B	W	I	0	96	98	97	96	0	89
Gm04.VB.41	Gm04	7550000	7750000	200000	9	W	W	W	W	W	W	I	12	46	51	41	41	30	907
Gm04.VB.42	Gm04	7750000	7880000	130000	6	W	B	W	B	W	W	I	13	296	13	289	9	15	475
Gm04.VB.43	Gm04	7880000	7900000	20000	2	W	W	S	W	W	W	I	0	0	35	0	0	0	69
Gm04.VB.44	Gm04	7900000	7960000	60000	6	W	B	S	B	D	W	I	1	115	215	120	64	1	145
Gm04.VB.45	Gm04	7960000	8100000	140000	14	W	W	S	W	W	W	I	12	21	722	19	14	12	376
Gm04.VB.46	Gm04	8100000	8330000	230000	15	W	B	S	B	D	W	I	9	1381	1007	1357	506	9	819
Gm04.VB.47	Gm04	8330000	8450000	120000	10	W	W	W	W	W	W	I	1	5	7	8	5	1	334
Gm04.VB.48	Gm04	8450000	8960000	510000	30	W	W	S	W	W	W	I	30	69	2483	67	63	22	944
Gm04.VB.49	Gm04	8960000	9210000	250000	15	W	B	S	B	B	W	I	22	1454	1258	1444	1320	20	860
Gm04.VB.50	Gm04	9210000	9340000	130000	8	W	B	W	B	B	W	I	0	764	4	762	748	0	608
Gm04.VB.51	Gm04	9340000	12370000	3030000	89	W	B	S	B	B	W	I	234	7788	10107	7755	7582	222	6791
Gm04.VB.52	Gm04	12370000	12500000	130000	1	W	W	W	W	W	W	I	1	21	18	20	21	1	291
Gm04.VB.53	Gm04	12500000	12540000	40000	0	W	W	W	W	D	W	I	0	15	16	14	17	0	137
Gm04.VB.54	Gm04	12540000	12750000	210000	5	W	W	W	W	W	W	I	8	59	54	54	55	8	553
Gm04.VB.55	Gm04	12750000	12790000	40000	1	W	B	B	B	B	W	I	1	19	21	20	20	1	198
Gm04.VB.56	Gm04	12790000	12940000	150000	5	W	W	W	W	W	W	I	2	18	16	19	23	5	78
Gm04.VB.57	Gm04	12940000	13010000	70000	0	W	W	W	W	W	W	I	10	19	21	18	14	5	37
Gm04.VB.58	Gm04	13010000	28140000	15130000	205	W	B	B	B	B	W	I	692	24569	24932	24473	24557	676	19436
Gm04.VB.59	Gm04	28140000	29580000	1440000	10	W	W	W	W	W	W	I	75	169	173	158	157	58	1323
Gm04.VB.60	Gm04	29580000	33780000	4200000	38	W	B	B	B	B	W	I	174	25086	25105	24681	25231	157	4189
Gm04.VB.61	Gm04	33780000	36780000	3000000	31	W	W	W	W	W	W	I	130	307	284	309	276	164	4293
Gm04.VB.62	Gm04	36780000	36790000	10000	0	W	B	W	B	W	W	I	0	5	3	5	4	0	13
Gm04.VB.63	Gm04	36790000	37400000	610000	14	W	B	B	B	B	W	I	27	1670	1707	1663	1703	22	2196
Gm04.VB.64	Gm04	37400000	37510000	110000	1	W	W	W	W	W	W	I	1	22	23	25	20	1	638

Gm04.VB.65	Gm04	37510000	38230000	720000	12	W	B	B	B	B	W	I	25	932	929	929	899	17	1546
Gm04.VB.66	Gm04	38230000	38780000	550000	15	W	W	W	W	W	W	I	35	71	79	65	70	39	745
Gm04.VB.67	Gm04	38780000	39240000	460000	14	W	B	B	B	B	W	I	15	2395	2459	2360	2372	16	535
Gm04.VB.68	Gm04	39240000	39410000	170000	3	W	W	W	W	W	W	I	3	51	45	49	46	2	746
Gm04.VB.69	Gm04	39410000	39630000	220000	7	W	W	W	W	W	W	I	33	49	52	42	56	22	1245
Gm04.VB.70	Gm04	39630000	39660000	30000	1	W	W	W	W	W	W	I	0	9	9	10	10	0	155
Gm04.VB.71	Gm04	39660000	39760000	100000	6	W	B	B	B	B	W	I	8	263	264	256	261	5	70
Gm04.VB.72	Gm04	39760000	39770000	10000	0	W	B	S	B	W	W	W	2	8	10	7	4	2	3
Gm04.VB.73	Gm04	39770000	39870000	100000	3	W	W	W	W	W	W	I	16	31	29	30	32	15	69
Gm04.VB.74	Gm04	39870000	39910000	40000	0	W	W	W	W	W	W	I	0	2	1	2	2	0	21
Gm04.VB.75	Gm04	39910000	40440000	530000	12	W	W	S	W	W	W	I	67	86	536	78	74	52	610
Gm04.VB.76	Gm04	40440000	40640000	200000	8	W	W	W	W	W	W	I	13	30	34	33	28	21	989
Gm04.VB.77	Gm04	40640000	40650000	10000	0	W	W	S	W	W	W	I	0	2	19	2	1	0	42
Gm04.VB.78	Gm04	40650000	40850000	200000	9	W	W	W	W	W	W	I	24	42	42	46	45	24	606
Gm04.VB.79	Gm04	40850000	41060000	210000	10	W	B	W	B	B	W	I	33	962	32	939	892	19	669
Gm04.VB.80	Gm04	41060000	41080000	20000	1	W	W	W	W	W	W	I	0	7	0	7	5	0	46
Gm04.VB.81	Gm04	41080000	41200000	120000	9	W	W	W	W	W	W	I	1	7	3	8	7	1	626
Gm04.VB.82	Gm04	41200000	42300000	1100000	51	W	B	W	B	B	W	I	41	6574	41	6503	6272	36	3761
Gm04.VB.83	Gm04	42300000	42790000	490000	29	W	W	W	W	W	W	I	12	21	12	16	22	11	1630
Gm04.VB.84	Gm04	42790000	43060000	270000	20	W	B	W	W	B	W	I	22	1202	22	22	1098	20	1088
Gm04.VB.85	Gm04	43060000	43640000	580000	44	W	W	W	W	W	W	I	30	37	26	23	27	25	1493
Gm04.VB.86	Gm04	43640000	44390000	750000	58	W	W	S	S	W	W	I	58	79	3263	3196	50	47	2061
Gm04.VB.87	Gm04	44390000	44720000	330000	32	W	W	W	W	W	W	I	50	58	56	49	43	40	784
Gm04.VB.88	Gm04	44720000	44800000	80000	7	W	B	W	B	W	W	I	0	184	0	181	1	0	178
Gm04.VB.89	Gm04	44800000	45020000	220000	19	W	W	W	W	W	W	I	3	17	2	18	4	9	732
Gm04.VB.90	Gm04	45020000	45410000	390000	36	W	B	W	B	W	H	I	14	1823	16	1784	19	1209	1209
Gm04.VB.91	Gm04	45410000	45510000	100000	11	W	W	W	W	W	W	I	11	17	14	15	10	14	264
Gm04.VB.92	Gm04	45510000	45620000	110000	11	W	W	S	S	W	W	I	10	9	260	261	10	10	333
Gm04.VB.93	Gm04	45620000	45930000	310000	31	W	W	W	W	W	W	I	15	29	24	24	25	25	772
Gm04.VB.94	Gm04	45930000	45980000	50000	2	W	B	W	W	W	W	I	4	175	4	4	5	4	137
Gm04.VB.95	Gm04	45980000	46080000	100000	8	W	B	W	W	D	D	I	22	334	20	20	265	265	194
Gm04.VB.96	Gm04	46080000	46210000	130000	14	W	B	W	W	W	W	I	3	246	3	3	17	15	205
Gm04.VB.97	Gm04	46210000	46880000	670000	76	W	B	W	W	D	D	I	46	2917	50	42	2653	2540	1697
Gm04.VB.98	Gm04	46880000	46980000	100000	12	W	B	W	W	W	W	I	0	304	0	0	2	2	122
Gm04.VB.99	Gm04	46980000	46990000	10000	0	W	W	W	W	W	W	I	0	2	0	0	0	0	19
Gm04.VB.100	Gm04	46990000	47120000	130000	9	W	W	W	W	D	H	I	5	9	5	5	476	407	290
Gm04.VB.101	Gm04	47120000	47380000	260000	28	W	B	W	W	D	H	I	4	917	4	3	501	417	589
Gm04.VB.102	Gm04	47380000	47530000	150000	15	W	W	W	W	D	H	I	14	19	14	13	260	193	218
Gm04.VB.103	Gm04	47530000	47760000	230000	23	W	B	W	W	B	H	I	2	786	2	2	729	539	346
Gm04.VB.104	Gm04	47760000	47960000	200000	16	W	W	W	W	W	H	I	9	18	10	9	16	501	316
Gm04.VB.105	Gm04	47960000	48230000	270000	26	W	B	W	W	B	H	I	17	1213	17	17	1111	566	830
Gm04.VB.106	Gm04	48230000	48370000	140000	15	W	W	W	W	W	H	I	0	18	0	0	15	342	532
Gm04.VB.107	Gm04	48370000	48470000	100000	13	W	B	W	W	B	H	I	3	411	0	2	373	42	270
Gm04.VB.108	Gm04	48470000	48770000	300000	29	W	W	W	W	W	W	I	11	28	14	11	29	33	690
Gm04.VB.109	Gm04	48770000	48880000	110000	10	W	B	W	W	B	H	I	2	357	2	2	318	200	343
Gm04.VB.110	Gm04	48880000	48990000	110000	14	W	W	W	W	W	H	I	8	15	9	5	14	369	198
Gm04.VB.111	Gm04	48990000	49100000	110000	14	W	B	W	W	B	W	W	0	330	0	0	322	4	41
Gm04.VB.112	Gm04	49100000	49240000	140000	9	W	W	W	W	W	W	W	6	15	6	6	11	11	42
Gm04.VB.113	Gm04	49240000	49243852	3852	1	W	W	W	W	W	W	I	0	0	0	0	0	1	2
Gm05.VB.1	Gm05	0	380000	380000	43	W	B	W	B	W	W	I	10	442	12	441	8	13	911
Gm05.VB.2	Gm05	380000	420000	40000	4	W	B	W	B	W	B	I	0	138	0	141	0	135	112
Gm05.VB.3	Gm05	420000	660000	240000	27	W	W	W	W	W	H	I	7	15	3	13	2	477	386
Gm05.VB.4	Gm05	660000	710000	50000	4	W	B	W	B	W	H	I	12	189	12	183	12	224	155
Gm05.VB.5	Gm05	710000	860000	150000	17	W	W	W	W	W	H	I	14	27	14	27	13	176	366
Gm05.VB.6	Gm05	860000	1170000	310000	29	W	B	W	B	W	H	I	5	986	5	970	5	912	820
Gm05.VB.7	Gm05	1170000	1240000	70000	9	W	B	W	W	W	H	I	0	332	0	0	0	145	194
Gm05.VB.8	Gm05	1240000	1530000	290000	30	W	B	W	W	W	W	I	25	1790	23	27	13	26	928
Gm05.VB.9	Gm05	1530000	1610000	80000	8	W	B	W	W	W	H	I	2	290	1	2	2	337	243
Gm05.VB.10	Gm05	1610000	1790000	180000	20	W	B	W	W	W	W	I	1	860	0	0	0	6	391
Gm05.VB.11	Gm05	1790000	1970000	180000	12	W	W	W	W	W	H	I	15	23	15	13	10	428	466
Gm05.VB.12	Gm05	1970000	2070000	100000	12	W	B	W	W	W	H	I	2	381	2	2	2	370	286
Gm05.VB.13	Gm05	2070000	2090000	20000	1	W	B	W	W	W	W	I	0	79	0	0	0	1	82
Gm05.VB.14	Gm05	2090000	2640000	550000	61	W	W	W	W	W	W	I	44	66	42	42	41	59	1330
Gm05.VB.15	Gm05	2640000	2720000	80000	7	W	B	W	W	W	B	I	12	449	15	12	13	413	144
Gm05.VB.16	Gm05	2720000	2950000	230000	20	W	W	W	W	W	W	I	5	18	5	5	5	15	605
Gm05.VB.17	Gm05	2950000	2970000	20000	4	W	B	W	W	W	B	I	3	104	3	2	2	89	20
Gm05.VB.18	Gm05	2970000	3410000	440000	39	W	W	W	W	W	W	I	49	66	51	49	47	60	1233
Gm05.VB.19	Gm05	3410000	3740000	330000	25	W	B	W	W	B	B	I	57	2278	49	40	2153	2176	1177
Gm05.VB.20	Gm05	3740000	3880000	140000	11	W	W	W	W	W	W	I	16	25	17	18	12	21	382
Gm05.VB.21	Gm05	3880000	3910000	30000	2	W	B	W	W	W	B	I	0	90	0	0	0	86	74
Gm05.VB.22	Gm05	3910000	5070000	1160000	53	W	W	W	W	W	W	I	135	175	135	119	127	142	3898
Gm05.VB.23	Gm05	5070000	5140000	70000	7	W	W	S	S	S	W	I	19	21	138	142	137	16	328
Gm05.VB.24	Gm05	5140000	5700000	560000	25	W	W	W	W	W	W	I	55	86	98	103	97	74	1184
Gm05.VB.25	Gm05	5700000	5710000	10000	1	W	W	W	W	D	W	I	0	1	4	4	5	1	35
Gm05.VB.26	Gm05	5710000	5750000	40000	1	W	W	S	S	D	W	I	5	6	365	363	327	4	147
Gm05.VB.27	Gm05	5750000	7450000	1700000	33	W	W	W	W	W	W	I	68	120	166	143	156	97	6906
Gm05.VB.28	Gm05	7450000	7510000	60000	6	W	B	W	B	W	W	I	1	410	1	414	1	5	229
Gm05.VB.29	Gm05	7510000	7900000	390000	42	W	B	W	B	W	H	I	5	980	4	947	4	963	815

Gm05.VB.30	Gm05	7900000	8000000	100000	9	W	W	W	W	W	H	I	2	12	2	13	2	200	182
Gm05.VB.31	Gm05	8000000	8200000	200000	14	W	B	W	B	W	H	I	39	346	41	341	36	820	465
Gm05.VB.32	Gm05	8200000	8250000	50000	4	W	B	W	B	W	W	I	22	148	28	138	19	29	108
Gm05.VB.33	Gm05	8250000	8470000	220000	26	W	W	W	W	W	W	I	18	26	22	30	16	24	540
Gm05.VB.34	Gm05	8470000	8520000	50000	2	W	B	W	B	W	W	I	0	150	0	147	0	1	106
Gm05.VB.35	Gm05	8520000	8580000	60000	5	W	W	W	W	W	W	I	0	0	0	0	0	3	152
Gm05.VB.36	Gm05	8580000	8920000	340000	34	W	W	W	W	W	H	I	18	20	16	23	10	570	723
Gm05.VB.37	Gm05	8920000	8960000	40000	7	W	W	W	W	W	W	I	0	0	0	0	0	1	63
Gm05.VB.38	Gm05	8960000	9120000	160000	17	W	B	W	B	W	W	I	0	313	0	322	0	3	325
Gm05.VB.39	Gm05	9120000	22160000	13040000	121	W	W	W	W	W	W	I	699	1190	1953	1862	1885	972	17695
Gm05.VB.40	Gm05	22160000	22200000	40000	0	W	W	W	W	D	W	I	26	25	37	43	37	36	68
Gm05.VB.41	Gm05	22200000	26660000	4460000	56	W	W	W	W	W	W	I	240	381	604	564	629	329	11391
Gm05.VB.42	Gm05	26660000	26730000	70000	6	W	W	S	S	S	W	I	0	5	413	404	375	1	173
Gm05.VB.43	Gm05	26730000	27030000	300000	11	W	W	W	W	D	W	I	3	6	23	23	2049	7	1286
Gm05.VB.44	Gm05	27030000	27210000	180000	2	W	W	W	W	W	W	I	13	18	23	24	20	14	388
Gm05.VB.45	Gm05	27210000	27350000	140000	6	W	W	S	S	W	W	I	4	11	235	231	13	9	449
Gm05.VB.46	Gm05	27350000	27440000	90000	3	W	W	S	S	D	W	I	47	39	782	756	551	42	269
Gm05.VB.47	Gm05	27440000	27530000	90000	3	W	W	W	W	D	W	I	0	4	2	2	329	2	443
Gm05.VB.48	Gm05	27530000	28690000	1160000	35	W	B	W	W	B	B	I	110	6984	141	135	6656	6698	5139
Gm05.VB.49	Gm05	28690000	28900000	210000	8	W	W	S	S	W	W	I	22	26	1311	1267	25	25	1259
Gm05.VB.50	Gm05	28900000	29140000	240000	3	W	W	W	W	W	W	I	56	62	79	69	68	54	875
Gm05.VB.51	Gm05	29140000	29150000	10000	1	W	B	W	W	W	W	I	0	34	0	0	0	2	24
Gm05.VB.52	Gm05	29150000	29340000	190000	3	W	B	W	W	W	H	I	20	517	35	26	32	119	375
Gm05.VB.53	Gm05	29340000	29600000	260000	12	W	B	W	W	W	W	I	19	1923	39	38	32	27	748
Gm05.VB.54	Gm05	29600000	30630000	1030000	27	W	W	W	W	W	W	I	31	42	120	113	93	92	3731
Gm05.VB.55	Gm05	30630000	30730000	100000	7	W	W	W	W	W	H	I	26	25	32	35	30	365	331
Gm05.VB.56	Gm05	30730000	30810000	80000	9	W	W	S	S	D	H	I	7	7	207	199	164	184	225
Gm05.VB.57	Gm05	30810000	30920000	110000	5	W	W	W	W	W	W	I	3	2	2	2	4	5	122
Gm05.VB.58	Gm05	30920000	31100000	180000	14	W	W	S	S	S	H	I	3	2	642	628	610	478	376
Gm05.VB.59	Gm05	31100000	31200000	100000	10	W	W	S	S	S	W	I	5	5	581	556	535	11	329
Gm05.VB.60	Gm05	31200000	31300000	100000	5	W	W	S	S	S	S	I	15	15	523	506	489	550	425
Gm05.VB.61	Gm05	31300000	31410000	110000	10	W	W	W	W	W	H	I	4	4	14	15	12	583	148
Gm05.VB.62	Gm05	31410000	31730000	320000	33	W	W	S	S	S	H	I	39	31	1062	1038	988	676	921
Gm05.VB.63	Gm05	31730000	31890000	160000	12	W	B	S	S	S	H	I	2	451	838	798	753	470	392
Gm05.VB.64	Gm05	31890000	31940000	50000	3	W	B	W	W	W	W	I	2	72	3	3	2	3	93
Gm05.VB.65	Gm05	31940000	32040000	100000	11	W	W	W	W	W	W	I	1	5	5	5	5	8	387
Gm05.VB.66	Gm05	32040000	32090000	50000	5	W	W	W	W	W	H	I	4	7	4	4	4	79	119
Gm05.VB.67	Gm05	32090000	32160000	70000	5	W	B	W	W	W	H	I	3	329	8	8	7	78	234
Gm05.VB.68	Gm05	32160000	32320000	160000	14	W	W	W	W	W	W	I	3	9	7	4	5	6	279
Gm05.VB.69	Gm05	32320000	32340000	20000	2	W	B	B	B	B	B	I	4	98	90	91	86	84	51
Gm05.VB.70	Gm05	32340000	32490000	150000	20	W	B	W	W	W	H	I	4	158	12	9	12	95	385
Gm05.VB.71	Gm05	32490000	32810000	320000	29	W	B	B	B	B	H	I	33	910	917	894	843	597	862
Gm05.VB.72	Gm05	32810000	33060000	250000	19	W	B	W	B	W	B	I	39	889	45	858	32	817	421
Gm05.VB.73	Gm05	33060000	33330000	270000	18	W	B	S	B	S	B	I	13	669	1289	648	1138	625	751
Gm05.VB.74	Gm05	33330000	34730000	1400000	140	W	W	S	W	S	W	I	65	71	5891	68	5120	86	3421
Gm05.VB.75	Gm05	34730000	34790000	60000	6	W	W	S	W	D	H	I	4	3	143	4	117	207	174
Gm05.VB.76	Gm05	34790000	34830000	40000	4	W	W	W	W	W	W	I	1	8	9	8	8	2	38
Gm05.VB.77	Gm05	34830000	34890000	60000	6	W	B	B	B	B	W	I	3	208	209	211	199	7	140
Gm05.VB.78	Gm05	34890000	35000000	110000	9	W	B	B	B	B	H	I	14	583	602	557	526	456	272
Gm05.VB.79	Gm05	35000000	35940000	940000	98	W	W	S	W	S	H	I	60	74	4234	72	3805	3367	2504
Gm05.VB.80	Gm05	35940000	36000000	60000	9	W	W	W	W	W	H	I	8	6	10	8	5	140	136
Gm05.VB.81	Gm05	36000000	36140000	140000	17	W	W	W	W	W	W	I	6	9	14	9	13	21	339
Gm05.VB.82	Gm05	36140000	36150000	10000	1	W	W	S	W	W	H	I	0	0	5	0	4	7	34
Gm05.VB.83	Gm05	36150000	36180000	30000	1	W	W	S	W	S	H	I	12	13	163	13	156	28	162
Gm05.VB.84	Gm05	36180000	36200000	20000	0	W	W	W	W	W	W	I	0	0	8	1	8	0	42
Gm05.VB.85	Gm05	36200000	36320000	120000	16	W	W	W	W	W	W	I	2	13	9	13	10	11	374
Gm05.VB.86	Gm05	36320000	36610000	290000	30	W	W	S	W	S	W	I	14	28	1222	28	1106	25	712
Gm05.VB.87	Gm05	36610000	36930000	320000	38	W	W	S	W	S	H	I	10	51	826	52	747	423	659
Gm05.VB.88	Gm05	36930000	37160000	230000	27	W	B	S	B	S	H	I	17	1245	619	1203	555	649	626
Gm05.VB.89	Gm05	37160000	38110000	950000	104	W	B	W	B	W	H	I	56	4145	61	4100	59	3370	2574
Gm05.VB.90	Gm05	38110000	38120000	10000	1	W	W	W	W	W	H	W	0	0	1	0	1	15	4
Gm05.VB.91	Gm05	38120000	38240000	120000	12	W	W	W	W	W	W	I	0	2	0	0	2	7	162
Gm05.VB.92	Gm05	38240000	38280000	40000	6	W	W	W	W	W	H	I	4	6	4	4	6	24	153
Gm05.VB.93	Gm05	38280000	38390000	110000	15	W	W	W	W	W	W	I	3	10	3	2	5	17	181
Gm05.VB.94	Gm05	38390000	38440000	50000	7	W	W	W	W	W	H	I	0	2	0	0	2	29	97
Gm05.VB.95	Gm05	38440000	38480000	40000	5	W	B	W	W	B	B	I	0	155	0	0	129	129	59
Gm05.VB.96	Gm05	38480000	38580000	100000	13	W	W	W	W	W	W	I	2	7	2	2	8	5	54
Gm05.VB.97	Gm05	38580000	38600000	20000	3	W	B	W	W	B	B	I	0	46	0	0	37	40	47
Gm05.VB.98	Gm05	38600000	38830000	230000	26	W	B	W	W	B	W	I	10	417	5	5	364	6	442
Gm05.VB.99	Gm05	38830000	38980000	150000	21	W	W	W	W	W	W	I	0	0	0	1	0	0	239
Gm05.VB.100	Gm05	38980000	38990000	10000	2	W	W	W	W	W	H	I	0	1	0	1	1	7	10
Gm05.VB.101	Gm05	38990000	39800000	810000	89	W	B	W	B	B	H	I	21	3513	24	3464	3215	2610	1917
Gm05.VB.102	Gm05	39800000	39910000	110000	12	W	W	W	W	W	W	I	7	17	7	17	16	10	147
Gm05.VB.103	Gm05	39910000	39990000	80000	10	W	B	W	B	B	W	I	0	134	0	135	126	7	59
Gm05.VB.104	Gm05	39990000	40000000	10000	2	W	B	W	B	B	H	I	0	58	0	58	51	13	42
Gm05.VB.105	Gm05	40000000	41930000	1930000	218	W	B	W	B	W	H	I	67	7409	68	7325	57	7031	4118
Gm05.VB.106	Gm05	41930000	41936504	6504	0	W	B	W	B	W	H	I	5	42	6	42	6	45	7
Gm06.VB.1	Gm06	0	610000	610000	70	W	W	W	W	W	H	I	29	55	51	46	41	1406	579

Gm06.VB.2	Gm06	610000	1620000	1010000	115	W	B	W	W	W	H	I	22	3290	37	34	35	758	1811
Gm06.VB.3	Gm06	1620000	1730000	110000	12	W	W	W	W	W	W	I	3	13	4	6	6	7	314
Gm06.VB.4	Gm06	1730000	1750000	20000	1	W	B	W	W	W	W	I	0	40	2	2	2	3	19
Gm06.VB.5	Gm06	1750000	1820000	70000	8	W	B	W	W	W	W	I	1	246	2	2	1	9	108
Gm06.VB.6	Gm06	1820000	1830000	10000	2	W	W	W	W	W	W	I	0	0	3	3	3	3	27
Gm06.VB.7	Gm06	1830000	1910000	80000	7	W	W	S	S	S	S	I	10	9	72	73	64	63	77
Gm06.VB.8	Gm06	1910000	2060000	150000	15	W	W	W	W	W	W	I	2	3	19	21	19	15	128
Gm06.VB.9	Gm06	2060000	2080000	20000	2	W	W	S	S	S	W	I	0	4	12	12	10	2	31
Gm06.VB.10	Gm06	2080000	2170000	90000	9	W	B	B	B	B	B	I	1	223	220	217	196	192	186
Gm06.VB.11	Gm06	2170000	2620000	450000	55	W	W	S	S	D	W	I	41	39	716	708	446	43	650
Gm06.VB.12	Gm06	2620000	3100000	480000	56	W	B	W	W	B	H	I	19	1515	38	38	1381	838	1072
Gm06.VB.13	Gm06	3100000	3110000	10000	1	W	B	S	S	B	B	I	0	41	43	48	38	40	21
Gm06.VB.14	Gm06	3110000	3240000	130000	11	W	B	S	S	B	W	I	6	500	528	512	458	5	268
Gm06.VB.15	Gm06	3240000	3480000	240000	32	W	W	S	S	W	W	I	8	13	615	600	10	5	503
Gm06.VB.16	Gm06	3480000	3640000	160000	20	W	B	S	S	B	W	I	3	337	481	459	303	6	328
Gm06.VB.17	Gm06	3640000	3780000	140000	10	W	W	W	W	W	W	I	18	31	19	19	25	18	176
Gm06.VB.18	Gm06	3780000	3930000	150000	20	W	B	W	W	B	W	I	0	479	2	2	440	0	291
Gm06.VB.19	Gm06	3930000	3970000	40000	4	W	W	S	S	W	W	I	8	20	150	151	22	6	69
Gm06.VB.20	Gm06	3970000	4090000	120000	10	W	W	W	W	W	W	I	14	55	36	36	52	13	173
Gm06.VB.21	Gm06	4090000	4110000	20000	4	W	B	S	G	D	W	I	0	32	33	30	27	1	21
Gm06.VB.22	Gm06	4110000	4520000	410000	46	W	B	W	W	B	W	I	27	449	35	32	413	36	539
Gm06.VB.23	Gm06	4520000	5020000	500000	51	W	W	W	W	W	W	I	15	39	18	20	37	18	676
Gm06.VB.24	Gm06	5020000	5090000	70000	8	W	B	W	W	B	H	W	1	281	2	2	260	52	2
Gm06.VB.25	Gm06	5090000	5340000	250000	27	W	B	W	W	B	W	W	31	1218	31	29	1051	28	15
Gm06.VB.26	Gm06	5340000	5700000	360000	40	W	W	W	W	W	W	W	28	35	40	39	29	26	7
Gm06.VB.27	Gm06	5700000	5950000	250000	26	W	W	S	S	W	W	I	5	7	648	623	7	5	388
Gm06.VB.28	Gm06	5950000	6010000	60000	5	W	W	W	W	W	W	I	31	35	37	38	34	34	174
Gm06.VB.29	Gm06	6010000	6140000	130000	16	W	B	W	W	W	W	I	1	223	1	1	0	1	150
Gm06.VB.30	Gm06	6140000	6260000	120000	11	W	W	W	W	W	W	I	1	6	5	4	4	1	143
Gm06.VB.31	Gm06	6260000	6320000	60000	6	W	B	W	W	W	W	I	0	35	1	1	1	1	79
Gm06.VB.32	Gm06	6320000	6450000	130000	16	W	W	W	W	W	W	I	7	16	8	7	2	1	199
Gm06.VB.33	Gm06	6450000	6670000	220000	23	W	B	W	W	W	W	I	33	525	35	35	29	30	273
Gm06.VB.34	Gm06	6670000	6940000	270000	27	W	W	W	W	W	W	W	17	31	19	16	18	13	11
Gm06.VB.35	Gm06	6940000	7350000	410000	32	W	B	W	W	W	W	I	8	492	10	10	30	10	177
Gm06.VB.36	Gm06	7350000	7360000	10000	0	W	B	W	W	B	W	I	0	54	0	0	47	0	23
Gm06.VB.37	Gm06	7360000	8010000	650000	64	W	W	W	W	W	W	I	29	68	29	30	41	20	959
Gm06.VB.38	Gm06	8010000	8380000	370000	36	W	B	W	W	W	W	I	15	503	18	13	11	14	503
Gm06.VB.39	Gm06	8380000	9010000	630000	57	W	W	W	W	W	W	I	46	69	48	50	48	41	1261
Gm06.VB.40	Gm06	9010000	9450000	440000	48	W	B	W	W	B	W	I	25	870	26	26	809	20	681
Gm06.VB.41	Gm06	9450000	9550000	100000	11	W	W	W	W	W	W	I	13	30	15	8	33	15	293
Gm06.VB.42	Gm06	9550000	9620000	70000	7	W	B	W	W	B	W	I	4	56	4	4	53	4	120
Gm06.VB.43	Gm06	9620000	9960000	340000	27	W	B	W	W	W	W	I	14	868	13	14	9	9	696
Gm06.VB.44	Gm06	9960000	10100000	140000	20	W	W	W	W	W	W	I	2	14	2	2	2	2	457
Gm06.VB.45	Gm06	10100000	10180000	80000	7	W	B	W	W	W	W	I	4	102	3	4	2	4	196
Gm06.VB.46	Gm06	10180000	10660000	480000	46	W	B	W	W	W	B	I	32	1025	33	31	25	961	653
Gm06.VB.47	Gm06	10660000	10780000	120000	12	W	W	W	W	W	H	I	9	15	11	11	10	86	207
Gm06.VB.48	Gm06	10780000	10810000	30000	1	W	B	W	W	W	H	I	3	85	3	2	2	36	47
Gm06.VB.49	Gm06	10810000	11140000	330000	40	W	B	W	W	W	W	I	11	918	10	10	10	13	418
Gm06.VB.50	Gm06	11140000	11300000	160000	22	W	W	W	W	W	W	I	11	9	10	12	2	7	393
Gm06.VB.51	Gm06	11300000	11500000	200000	22	W	B	W	W	W	W	I	4	409	4	7	5	5	440
Gm06.VB.52	Gm06	11500000	11620000	120000	14	W	W	W	W	W	W	I	0	13	0	0	0	2	386
Gm06.VB.53	Gm06	11620000	11960000	340000	35	W	B	W	W	W	W	I	16	551	17	15	15	14	766
Gm06.VB.54	Gm06	11960000	12040000	80000	6	W	B	B	B	B	W	I	1	108	120	120	116	6	138
Gm06.VB.55	Gm06	12040000	12120000	80000	8	W	W	W	W	W	W	I	5	10	11	11	7	7	208
Gm06.VB.56	Gm06	12120000	12210000	90000	10	W	W	W	W	W	H	I	0	6	4	4	4	105	225
Gm06.VB.57	Gm06	12210000	12550000	340000	39	W	B	B	B	B	H	I	12	964	978	967	834	613	875
Gm06.VB.58	Gm06	12550000	12580000	30000	5	W	W	W	W	W	H	I	0	2	2	2	2	42	39
Gm06.VB.59	Gm06	12580000	12960000	380000	36	W	W	W	W	W	W	I	7	19	20	20	19	13	1071
Gm06.VB.60	Gm06	12960000	13010000	50000	5	W	B	W	W	W	B	I	0	42	4	4	4	42	101
Gm06.VB.61	Gm06	13010000	13170000	160000	12	W	W	W	W	W	W	I	0	12	0	0	0	10	406
Gm06.VB.62	Gm06	13170000	13190000	20000	3	W	B	W	W	W	W	I	0	66	0	0	0	0	42
Gm06.VB.63	Gm06	13190000	13230000	40000	4	W	B	W	W	W	H	I	1	248	1	1	1	90	63
Gm06.VB.64	Gm06	13230000	13340000	110000	10	W	B	W	W	W	W	I	0	376	0	0	0	0	302
Gm06.VB.65	Gm06	13340000	13970000	630000	53	W	B	W	W	W	H	I	36	3052	32	29	23	1829	1772
Gm06.VB.66	Gm06	13970000	14160000	190000	19	W	B	W	W	W	W	I	19	645	25	24	14	25	534
Gm06.VB.67	Gm06	14160000	14430000	270000	25	W	B	W	W	W	B	I	26	962	27	20	21	886	523
Gm06.VB.68	Gm06	14430000	15210000	780000	55	W	W	W	W	W	H	I	86	66	73	66	53	651	1706
Gm06.VB.69	Gm06	15210000	15680000	470000	29	W	B	W	W	W	H	I	20	1490	19	20	14	1199	1036
Gm06.VB.70	Gm06	15680000	15710000	30000	1	W	B	W	W	W	W	I	5	88	5	5	5	11	55
Gm06.VB.71	Gm06	15710000	16380000	670000	48	W	W	W	W	W	W	I	43	50	43	40	37	39	2254
Gm06.VB.72	Gm06	16380000	16750000	370000	27	W	B	W	B	W	B	I	43	1819	43	1776	30	1696	1124
Gm06.VB.73	Gm06	16750000	17020000	270000	17	W	B	W	B	W	W	I	1	142	1	138	1	16	445
Gm06.VB.74	Gm06	17020000	25290000	8270000	183	W	B	W	B	W	B	I	315	28766	305	28595	247	28408	20185
Gm06.VB.75	Gm06	25290000	25300000	10000	0	W	B	W	G	W	W	W	17	24	15	18	12	8	4
Gm06.VB.76	Gm06	25300000	25450000	150000	0	W	W	W	W	W	W	W	10	27	12	27	7	12	7
Gm06.VB.77	Gm06	25450000	25470000	20000	0	W	W	W	W	W	W	I	8	3	5	6	3	6	10
Gm06.VB.78	Gm06	25470000	25810000	340000	3	W	B	W	B	W	B	I	22	491	24	488	35	504	420
Gm06.VB.79	Gm06	25810000	25930000	120000	1	W	W	W	W	W	W	I	1	10	1	9	1	18	203

Gm06.VB.80	Gm06	25930000	28660000	2730000	21	W	B	W	B	W	B	I	128	3962	143	3933	92	4016	3209
Gm06.VB.81	Gm06	28660000	28960000	300000	7	W	W	W	W	W	W	I	7	37	5	38	5	34	615
Gm06.VB.82	Gm06	28960000	29550000	590000	3	W	B	W	B	W	B	I	20	284	17	265	22	290	638
Gm06.VB.83	Gm06	29550000	31110000	1560000	10	W	W	W	W	W	W	I	303	866	296	841	299	852	1112
Gm06.VB.84	Gm06	31110000	31630000	520000	0	W	B	W	G	W	H	W	188	455	196	425	185	423	293
Gm06.VB.85	Gm06	31630000	32380000	750000	4	W	W	W	W	W	W	W	56	253	55	257	66	244	194
Gm06.VB.86	Gm06	32380000	32540000	160000	0	W	B	W	B	W	B	W	2	87	2	85	2	90	61
Gm06.VB.87	Gm06	32540000	33200000	660000	1	W	W	W	W	W	W	W	43	213	52	212	49	224	176
Gm06.VB.88	Gm06	33200000	33900000	700000	9	W	B	W	B	W	B	I	38	1856	39	1829	36	1870	1437
Gm06.VB.89	Gm06	33900000	37020000	3120000	39	W	W	W	W	W	W	I	148	334	161	321	125	338	11891
Gm06.VB.90	Gm06	37020000	37160000	140000	2	W	W	W	W	W	H	I	6	11	12	6	12	129	112
Gm06.VB.91	Gm06	37160000	37680000	520000	6	W	W	W	W	W	W	I	16	59	24	62	19	63	1275
Gm06.VB.92	Gm06	37680000	38300000	620000	15	W	B	W	B	W	B	I	30	1952	27	1939	30	1980	2994
Gm06.VB.93	Gm06	38300000	40690000	2390000	42	W	W	W	W	W	W	I	70	205	81	204	67	203	7516
Gm06.VB.94	Gm06	40690000	40840000	150000	3	W	B	W	B	W	B	I	31	826	38	810	33	825	1184
Gm06.VB.95	Gm06	40840000	41380000	540000	22	W	W	W	W	W	W	I	62	93	54	87	34	69	1962
Gm06.VB.96	Gm06	41380000	41480000	100000	5	W	B	W	B	W	B	I	0	869	0	865	0	826	390
Gm06.VB.97	Gm06	41480000	42750000	1270000	47	W	W	W	W	W	W	I	68	132	59	140	58	141	4685
Gm06.VB.98	Gm06	42750000	43460000	710000	19	W	B	W	B	W	B	I	31	3060	47	2954	43	2930	3326
Gm06.VB.99	Gm06	43460000	43800000	340000	14	W	W	W	W	W	W	I	3	19	5	20	3	19	1517
Gm06.VB.100	Gm06	43800000	44910000	1110000	58	W	B	W	B	W	B	I	81	7226	80	6933	69	6593	2701
Gm06.VB.101	Gm06	44910000	44930000	20000	2	W	B	W	W	W	B	I	0	116	0	3	0	116	68
Gm06.VB.102	Gm06	44930000	45290000	360000	15	W	W	W	W	W	W	I	12	29	13	11	12	24	1405
Gm06.VB.103	Gm06	45290000	45410000	120000	7	W	B	W	W	W	B	I	34	1095	35	35	29	1055	918
Gm06.VB.104	Gm06	45410000	45550000	140000	6	W	W	W	W	W	W	I	5	26	5	4	5	26	931
Gm06.VB.105	Gm06	45550000	45630000	80000	4	W	B	W	W	W	H	I	6	717	6	6	6	693	436
Gm06.VB.106	Gm06	45630000	45790000	160000	4	W	W	W	W	W	W	I	48	64	42	31	26	34	825
Gm06.VB.107	Gm06	45790000	46260000	470000	34	W	B	W	W	W	B	I	48	2818	42	42	36	2695	2125
Gm06.VB.108	Gm06	46260000	48330000	2070000	200	W	B	W	B	W	B	I	179	11430	187	11214	163	10560	7096
Gm06.VB.109	Gm06	48330000	48350000	20000	1	W	B	B	B	B	B	I	0	93	86	94	83	93	58
Gm06.VB.110	Gm06	48350000	49020000	670000	62	W	B	W	B	W	B	I	38	2622	39	2606	31	2472	2206
Gm06.VB.111	Gm06	49020000	49140000	120000	12	W	B	W	B	W	W	I	0	369	0	382	0	12	393
Gm06.VB.112	Gm06	49140000	49240000	100000	11	W	B	W	B	W	H	I	7	245	6	238	2	177	147
Gm06.VB.113	Gm06	49240000	49690000	450000	35	W	W	W	W	W	H	I	39	49	38	48	41	920	1460
Gm06.VB.114	Gm06	49690000	49730000	40000	3	W	B	W	B	W	H	I	0	122	0	121	0	115	71
Gm06.VB.115	Gm06	49730000	49850000	120000	13	W	W	W	W	W	H	I	1	9	2	9	2	277	393
Gm06.VB.116	Gm06	49850000	49860000	10000	2	W	W	W	W	W	W	I	0	0	0	0	0	1	64
Gm06.VB.117	Gm06	49860000	50020000	160000	17	W	B	W	B	W	W	I	0	942	0	931	0	8	576
Gm06.VB.118	Gm06	50020000	50050000	30000	3	W	B	W	B	W	H	I	0	159	0	160	0	73	92
Gm06.VB.119	Gm06	50050000	50530000	480000	53	W	B	W	B	B	H	I	43	1856	33	1825	1699	1099	1159
Gm06.VB.120	Gm06	50530000	50720000	190000	9	W	W	W	W	W	W	I	9	15	6	16	13	9	356
Gm06.VB.121	Gm06	50720000	50722821	2821	0	W	W	W	W	W	W	W	0	0	0	0	0	0	0
Gm07.VB.1	Gm07	0	210000	210000	23	W	B	S	B	S	H	I	8	505	451	506	410	408	282
Gm07.VB.2	Gm07	210000	330000	120000	16	W	W	S	W	S	H	I	3	7	206	9	194	182	143
Gm07.VB.3	Gm07	330000	430000	100000	15	W	W	W	W	W	H	I	0	1	8	2	6	371	261
Gm07.VB.4	Gm07	430000	450000	20000	3	W	W	S	W	S	H	I	0	0	25	0	22	47	34
Gm07.VB.5	Gm07	450000	590000	140000	17	W	W	S	W	S	W	I	16	20	265	20	250	14	133
Gm07.VB.6	Gm07	590000	1080000	490000	53	W	W	W	W	W	W	I	23	32	29	32	24	33	808
Gm07.VB.7	Gm07	1080000	1350000	270000	24	W	B	W	B	W	B	I	31	606	40	597	26	548	328
Gm07.VB.8	Gm07	1350000	1400000	50000	4	W	B	B	B	B	B	I	1	171	176	170	147	157	74
Gm07.VB.9	Gm07	1400000	1610000	210000	30	W	W	W	W	W	W	I	25	26	29	27	11	11	590
Gm07.VB.10	Gm07	1610000	1630000	20000	4	W	B	B	B	B	B	I	0	45	42	43	42	41	65
Gm07.VB.11	Gm07	1630000	1700000	70000	10	W	B	B	B	D	W	I	0	176	181	174	137	4	126
Gm07.VB.12	Gm07	1700000	1800000	100000	14	W	W	W	W	W	W	I	1	14	15	14	15	7	247
Gm07.VB.13	Gm07	1800000	1820000	20000	2	W	W	W	W	W	W	I	0	4	4	5	4	1	44
Gm07.VB.14	Gm07	1820000	1860000	40000	5	W	B	B	B	B	W	I	0	62	62	62	62	5	57
Gm07.VB.15	Gm07	1860000	1870000	10000	1	W	W	W	W	W	W	I	0	1	1	1	1	3	9
Gm07.VB.16	Gm07	1870000	2410000	540000	55	W	W	W	W	W	H	I	31	73	76	73	75	2230	1331
Gm07.VB.17	Gm07	2410000	2430000	20000	4	W	W	S	W	S	H	I	0	1	53	1	48	62	55
Gm07.VB.18	Gm07	2430000	2440000	10000	1	W	B	S	B	S	H	I	0	42	29	42	26	27	11
Gm07.VB.19	Gm07	2440000	2590000	150000	22	W	B	W	B	W	H	I	27	694	29	681	29	657	328
Gm07.VB.20	Gm07	2590000	2600000	10000	1	W	B	B	B	B	H	I	0	62	61	63	61	13	14
Gm07.VB.21	Gm07	2600000	3230000	630000	55	W	B	B	B	B	W	I	20	2993	3006	2939	2802	39	1659
Gm07.VB.22	Gm07	3230000	3310000	80000	6	W	B	W	B	W	W	I	2	399	2	391	2	7	189
Gm07.VB.23	Gm07	3310000	3390000	80000	8	W	B	W	B	W	H	I	0	232	0	231	0	211	136
Gm07.VB.24	Gm07	3390000	3490000	100000	10	W	B	W	B	W	W	I	24	75	24	73	23	30	207
Gm07.VB.25	Gm07	3490000	3810000	320000	36	W	W	W	W	W	W	I	14	21	13	15	7	17	668
Gm07.VB.26	Gm07	3810000	3980000	170000	20	W	B	W	W	W	W	I	3	616	5	4	3	4	298
Gm07.VB.27	Gm07	3980000	4130000	150000	12	W	W	W	W	W	W	I	0	6	0	0	0	7	291
Gm07.VB.28	Gm07	4130000	4180000	50000	6	W	B	W	W	W	B	I	2	170	2	2	1	157	82
Gm07.VB.29	Gm07	4180000	4340000	160000	10	W	W	W	W	W	W	I	32	36	32	32	21	33	327
Gm07.VB.30	Gm07	4340000	4380000	40000	4	W	W	W	W	W	H	I	0	3	0	0	0	98	83
Gm07.VB.31	Gm07	4380000	4470000	90000	5	W	B	W	W	W	B	I	1	296	1	1	1	314	165
Gm07.VB.32	Gm07	4470000	4580000	110000	6	W	W	W	W	W	H	I	20	21	20	15	12	252	232
Gm07.VB.33	Gm07	4580000	5080000	500000	44	W	W	W	W	W	W	I	61	78	66	59	45	60	1317
Gm07.VB.34	Gm07	5080000	5360000	280000	26	W	B	W	W	W	B	I	42	1190	37	33	27	1054	950
Gm07.VB.35	Gm07	5360000	5470000	110000	7	W	W	W	W	W	W	I	2	7	2	1	2	7	459
Gm07.VB.36	Gm07	5470000	5480000	10000	1	W	B	W	W	W	W	I	0	5	0	0	0	4	11

Gm07.VB.37	Gm07	5480000	6050000	570000	37	W	B	W	W	W	H	I	40	2492	34	36	20	2187	1194
Gm07.VB.38	Gm07	6050000	6510000	460000	42	W	W	W	W	W	W	I	43	65	44	40	27	43	1357
Gm07.VB.39	Gm07	6510000	7260000	750000	63	W	B	W	W	W	B	I	48	3726	57	51	45	3416	2560
Gm07.VB.40	Gm07	7260000	7430000	170000	10	W	W	W	W	W	H	I	1	13	3	3	1	476	385
Gm07.VB.41	Gm07	7430000	7540000	110000	13	W	W	W	W	W	W	I	22	34	24	20	12	30	94
Gm07.VB.42	Gm07	7540000	7680000	140000	10	W	B	W	W	W	W	I	41	744	41	41	15	48	394
Gm07.VB.43	Gm07	7680000	9050000	1370000	113	W	B	W	W	W	H	I	112	7801	103	101	76	7387	5048
Gm07.VB.44	Gm07	9050000	9250000	200000	12	W	W	W	W	W	H	I	7	28	8	12	1	656	866
Gm07.VB.45	Gm07	9250000	9310000	60000	0	W	B	W	W	W	H	I	5	569	8	15	4	634	178
Gm07.VB.46	Gm07	9310000	9420000	110000	5	W	W	W	W	W	H	I	0	12	0	0	0	920	457
Gm07.VB.47	Gm07	9420000	9560000	140000	12	W	B	W	W	W	H	I	18	774	18	9	18	915	638
Gm07.VB.48	Gm07	9560000	9730000	170000	12	W	W	W	W	W	H	I	4	12	4	5	4	1371	459
Gm07.VB.49	Gm07	9730000	9750000	20000	1	W	B	W	W	W	H	I	0	68	0	0	0	96	60
Gm07.VB.50	Gm07	9750000	9790000	40000	2	W	B	W	W	W	W	I	4	391	4	4	4	8	334
Gm07.VB.51	Gm07	9790000	9980000	190000	16	W	W	W	W	W	W	I	11	22	10	9	8	17	268
Gm07.VB.52	Gm07	9980000	10150000	170000	7	W	W	W	W	W	H	I	8	15	10	9	5	846	571
Gm07.VB.53	Gm07	10150000	10180000	30000	2	W	W	W	W	W	W	I	1	5	1	1	1	9	135
Gm07.VB.54	Gm07	10180000	10450000	270000	18	W	B	W	W	W	W	I	12	694	13	12	10	18	730
Gm07.VB.55	Gm07	10450000	10800000	350000	15	W	W	W	W	W	W	I	24	63	37	33	21	24	971
Gm07.VB.56	Gm07	10800000	10990000	190000	4	W	B	W	W	W	W	I	23	187	25	23	23	20	155
Gm07.VB.57	Gm07	10990000	14500000	3510000	99	W	W	W	W	W	W	I	155	294	172	157	149	272	6739
Gm07.VB.58	Gm07	14500000	14540000	40000	0	W	B	W	W	W	B	I	0	27	0	0	0	26	119
Gm07.VB.59	Gm07	14540000	14560000	20000	2	W	B	W	W	W	W	I	0	10	0	0	0	7	76
Gm07.VB.60	Gm07	14560000	14680000	120000	6	W	W	W	W	W	W	W	0	5	0	0	0	5	24
Gm07.VB.61	Gm07	14680000	14810000	130000	4	W	B	W	W	W	W	I	10	528	11	11	15	18	368
Gm07.VB.62	Gm07	14810000	15380000	570000	50	W	B	S	S	S	H	I	39	2726	3057	3010	2784	2092	1950
Gm07.VB.63	Gm07	15380000	15490000	110000	13	W	B	W	W	W	H	I	0	104	5	4	4	164	317
Gm07.VB.64	Gm07	15490000	15600000	110000	6	W	B	S	S	S	H	I	11	208	185	183	175	84	444
Gm07.VB.65	Gm07	15600000	15730000	130000	8	W	W	S	S	S	H	I	1	8	428	423	409	87	522
Gm07.VB.66	Gm07	15730000	15790000	60000	5	W	B	S	S	S	H	I	5	267	188	185	164	157	154
Gm07.VB.67	Gm07	15790000	15890000	100000	4	W	B	S	S	S	W	I	2	268	431	419	359	11	326
Gm07.VB.68	Gm07	15890000	16010000	120000	10	W	B	W	W	W	W	I	6	589	13	12	7	6	558
Gm07.VB.69	Gm07	16010000	16020000	10000	1	W	B	S	S	S	H	I	0	33	36	36	34	6	19
Gm07.VB.70	Gm07	16020000	16160000	140000	7	W	W	S	S	S	H	I	13	18	1267	1228	1170	191	633
Gm07.VB.71	Gm07	16160000	16210000	50000	4	W	B	S	S	S	H	I	19	462	98	98	92	53	372
Gm07.VB.72	Gm07	16210000	16320000	110000	2	W	W	W	W	W	W	I	42	56	49	47	34	37	71
Gm07.VB.73	Gm07	16320000	16440000	120000	8	W	B	W	W	W	W	I	1	913	3	3	2	3	736
Gm07.VB.74	Gm07	16440000	16450000	10000	1	W	B	S	S	S	W	I	10	83	5	5	5	1	53
Gm07.VB.75	Gm07	16450000	16510000	60000	9	W	W	S	S	S	H	I	69	70	262	261	238	53	166
Gm07.VB.76	Gm07	16510000	16780000	270000	17	W	W	S	S	S	W	I	102	112	974	903	986	100	705
Gm07.VB.77	Gm07	16780000	17010000	230000	13	W	W	S	S	S	H	I	180	190	1037	978	934	185	686
Gm07.VB.78	Gm07	17010000	17120000	110000	7	W	W	S	S	D	W	I	16	22	1146	1082	1002	16	397
Gm07.VB.79	Gm07	17120000	17210000	90000	6	W	B	B	B	B	B	I	9	254	280	279	266	240	149
Gm07.VB.80	Gm07	17210000	17340000	130000	1	W	W	W	W	W	W	W	20	30	37	36	32	29	36
Gm07.VB.81	Gm07	17340000	17630000	290000	10	W	W	S	S	S	W	I	40	53	1955	1893	1875	45	608
Gm07.VB.82	Gm07	17630000	17760000	130000	9	W	B	S	S	S	B	I	19	371	232	230	227	337	382
Gm07.VB.83	Gm07	17760000	17870000	110000	9	W	B	B	B	B	W	I	1	290	301	301	302	6	305
Gm07.VB.84	Gm07	17870000	17970000	100000	7	W	B	B	B	B	H	I	4	190	196	183	177	151	139
Gm07.VB.85	Gm07	17970000	17990000	20000	2	W	B	B	B	B	W	I	9	21	22	21	20	9	19
Gm07.VB.86	Gm07	17990000	18000000	10000	0	W	W	W	G	W	W	W	1	4	4	5	2	0	0
Gm07.VB.87	Gm07	18000000	18170000	170000	4	W	W	W	W	W	W	I	6	19	17	17	16	7	168
Gm07.VB.88	Gm07	18170000	18330000	160000	7	W	B	B	B	B	W	I	18	730	748	729	722	15	534
Gm07.VB.89	Gm07	18330000	18640000	310000	14	W	B	B	B	B	B	I	25	428	425	409	429	418	1071
Gm07.VB.90	Gm07	18640000	18820000	180000	5	W	W	W	W	W	W	I	1	20	21	20	21	38	1015
Gm07.VB.91	Gm07	18820000	18830000	10000	0	W	W	W	W	W	H	I	0	2	2	2	2	8	24
Gm07.VB.92	Gm07	18830000	19600000	770000	37	W	B	B	B	B	H	I	51	1677	1716	1677	1572	1691	2491
Gm07.VB.93	Gm07	19600000	20120000	520000	8	W	B	B	B	B	W	I	14	2067	2092	2026	2044	91	1646
Gm07.VB.94	Gm07	20120000	20890000	770000	9	W	B	B	B	B	H	I	43	881	900	890	880	388	700
Gm07.VB.95	Gm07	20890000	23810000	2920000	23	W	B	B	B	B	W	I	359	2351	2330	2345	2384	1188	1808
Gm07.VB.96	Gm07	23810000	23910000	100000	0	W	B	B	B	B	H	I	5	109	104	104	99	49	87
Gm07.VB.97	Gm07	23910000	24580000	670000	1	W	B	B	B	B	W	I	27	533	533	496	555	228	350
Gm07.VB.98	Gm07	24580000	24620000	40000	0	W	B	S	G	D	W	W	3	37	42	40	28	17	18
Gm07.VB.99	Gm07	24620000	25390000	770000	7	W	B	B	B	B	W	I	27	534	555	534	539	225	407
Gm07.VB.100	Gm07	25390000	25450000	60000	0	W	B	B	B	B	H	I	0	71	73	70	76	28	51
Gm07.VB.101	Gm07	25450000	26000000	550000	6	W	B	B	B	B	W	I	33	414	420	432	405	162	301
Gm07.VB.102	Gm07	26000000	26140000	140000	1	W	B	B	B	B	H	I	1	143	140	143	150	60	109
Gm07.VB.103	Gm07	26140000	26630000	490000	1	W	B	S	G	D	W	I	63	467	456	425	464	194	259
Gm07.VB.104	Gm07	26630000	26650000	20000	0	W	W	W	G	W	W	W	5	8	10	11	13	5	9
Gm07.VB.105	Gm07	26650000	26790000	140000	0	W	W	W	W	W	W	W	2	13	12	11	19	20	2
Gm07.VB.106	Gm07	26790000	26830000	40000	0	W	W	W	W	W	W	W	3	17	15	11	11	14	1
Gm07.VB.107	Gm07	26830000	28290000	1460000	16	W	B	B	B	B	W	I	71	1051	1053	1011	1061	467	781
Gm07.VB.108	Gm07	28290000	28350000	60000	0	W	B	S	G	S	H	W	0	61	54	46	54	30	23
Gm07.VB.109	Gm07	28350000	30270000	1920000	20	W	B	B	B	B	W	I	33	1347	1320	1332	1337	601	984
Gm07.VB.110	Gm07	30270000	34220000	3950000	44	W	W	W	W	W	W	I	156	383	397	360	411	623	3585
Gm07.VB.111	Gm07	34220000	34740000	520000	13	W	W	W	W	W	H	I	58	89	90	76	81	299	1133
Gm07.VB.112	Gm07	34740000	35040000	300000	4	W	W	W	W	W	W	I	7	34	40	35	37	105	388
Gm07.VB.113	Gm07	35040000	35230000	190000	19	W	B	B	B	B	W	I	2	212	214	212	201	4	328
Gm07.VB.114	Gm07	35230000	35340000	110000	11	W	W	W	W	W	W	I	0	7	6	7	5	4	250

Gm07.VB.115	Gm07	35340000	35490000	150000	15	W	W	W	W	W	H	I	7	15	14	12	5	70	426
Gm07.VB.116	Gm07	35490000	35640000	150000	14	W	B	B	B	B	H	I	7	765	767	763	690	364	399
Gm07.VB.117	Gm07	35640000	35930000	290000	23	W	W	W	W	W	W	I	17	24	23	24	25	87	705
Gm07.VB.118	Gm07	35930000	35980000	50000	5	W	B	B	B	B	B	I	8	201	203	204	197	185	162
Gm07.VB.119	Gm07	35980000	36080000	100000	10	W	W	W	W	W	W	I	17	24	21	21	17	16	300
Gm07.VB.120	Gm07	36080000	36150000	70000	4	W	B	W	W	W	W	I	12	386	16	16	10	11	225
Gm07.VB.121	Gm07	36150000	36190000	40000	2	W	W	W	W	W	W	I	0	3	3	4	4	1	82
Gm07.VB.122	Gm07	36190000	36230000	40000	4	W	W	S	S	S	W	I	8	12	101	99	99	8	27
Gm07.VB.123	Gm07	36230000	36330000	100000	5	W	W	S	S	D	H	I	5	8	275	287	251	205	109
Gm07.VB.124	Gm07	36330000	39020000	2690000	196	W	B	S	B	S	H	I	1982	10190	11355	10093	10436	9899	9156
Gm07.VB.125	Gm07	39020000	39100000	80000	9	W	B	S	B	S	W	I	0	100	227	98	209	2	164
Gm07.VB.126	Gm07	39100000	39240000	140000	8	W	W	S	W	S	W	I	2	15	526	14	489	5	217
Gm07.VB.127	Gm07	39240000	40940000	1700000	93	W	W	W	W	W	W	I	78	180	134	175	116	102	5415
Gm07.VB.128	Gm07	40940000	40970000	30000	1	W	B	W	B	W	W	I	10	228	7	217	4	5	114
Gm07.VB.129	Gm07	40970000	41850000	880000	49	W	W	W	W	W	W	I	22	102	24	89	21	42	2884
Gm07.VB.130	Gm07	41850000	41940000	90000	12	W	B	W	B	W	B	I	6	222	6	221	1	206	184
Gm07.VB.131	Gm07	41940000	42010000	70000	4	W	W	W	W	W	W	I	15	22	19	12	17	23	103
Gm07.VB.132	Gm07	42010000	42060000	50000	6	W	W	W	W	W	W	I	0	10	0	10	10	2	122
Gm07.VB.133	Gm07	42060000	42120000	60000	8	W	B	W	B	D	D	I	7	94	8	96	71	82	112
Gm07.VB.134	Gm07	42120000	42340000	220000	29	W	W	W	W	W	W	I	14	23	14	23	22	21	406
Gm07.VB.135	Gm07	42340000	42360000	20000	3	W	B	W	B	D	W	I	0	48	0	48	37	0	27
Gm07.VB.136	Gm07	42360000	42400000	40000	3	W	B	W	B	B	H	I	8	104	8	103	93	136	84
Gm07.VB.137	Gm07	42400000	42610000	210000	24	W	W	W	W	W	H	I	25	30	25	29	26	488	425
Gm07.VB.138	Gm07	42610000	42660000	50000	7	W	B	W	B	B	H	I	0	118	0	119	118	62	75
Gm07.VB.139	Gm07	42660000	42760000	100000	13	W	W	W	W	W	W	I	1	6	0	6	6	9	172
Gm07.VB.140	Gm07	42760000	42770000	10000	1	W	B	W	W	W	W	I	0	5	0	4	3	3	19
Gm07.VB.141	Gm07	42770000	42940000	170000	16	W	B	W	B	B	B	I	8	433	9	412	360	346	190
Gm07.VB.142	Gm07	42940000	43000000	60000	8	W	W	W	W	W	H	I	5	8	5	7	7	138	108
Gm07.VB.143	Gm07	43000000	44680000	1680000	202	W	W	W	W	W	W	I	55	66	54	53	35	55	3071
Gm07.VB.144	Gm07	44680000	44683157	3157	0	W	W	W	W	W	H	I	1	1	1	1	1	2	11
Gm08.VB.1	Gm08	0	350000	350000	39	W	W	W	W	W	W	I	10	10	19	9	10	20	736
Gm08.VB.2	Gm08	350000	490000	140000	16	W	W	S	W	W	W	I	4	6	185	6	10	10	345
Gm08.VB.3	Gm08	490000	530000	40000	5	W	W	W	W	W	W	I	1	1	9	1	2	1	74
Gm08.VB.4	Gm08	530000	620000	90000	10	W	W	W	W	W	H	I	11	11	13	11	11	89	134
Gm08.VB.5	Gm08	620000	640000	20000	0	W	W	S	W	W	W	I	0	0	31	0	0	2	20
Gm08.VB.6	Gm08	640000	950000	310000	36	W	W	W	W	W	H	I	22	22	56	23	20	540	653
Gm08.VB.7	Gm08	950000	1120000	170000	23	W	W	W	W	W	W	I	16	22	24	15	20	24	335
Gm08.VB.8	Gm08	1120000	1260000	140000	16	W	B	B	B	B	B	I	3	212	221	213	192	197	356
Gm08.VB.9	Gm08	1260000	1460000	200000	19	W	B	S	B	B	W	I	15	163	257	158	160	27	466
Gm08.VB.10	Gm08	1460000	1530000	70000	6	W	B	B	B	B	B	I	0	200	200	198	183	194	132
Gm08.VB.11	Gm08	1530000	1700000	170000	17	W	B	W	B	W	B	I	8	626	16	613	11	556	445
Gm08.VB.12	Gm08	1700000	1880000	180000	25	W	B	W	B	W	W	I	2	129	5	127	1	15	344
Gm08.VB.13	Gm08	1880000	2070000	190000	23	W	B	W	B	W	H	I	9	641	10	617	7	352	338
Gm08.VB.14	Gm08	2070000	2270000	200000	20	W	W	W	W	W	W	I	3	16	7	16	3	25	195
Gm08.VB.15	Gm08	2270000	3060000	790000	97	W	B	W	B	W	H	I	46	3248	48	3208	32	2606	2070
Gm08.VB.16	Gm08	3060000	3190000	130000	15	W	B	W	B	W	W	I	5	205	6	198	9	12	261
Gm08.VB.17	Gm08	3190000	3340000	150000	16	W	W	W	W	W	W	I	2	14	2	13	1	8	268
Gm08.VB.18	Gm08	3340000	3380000	40000	5	W	W	W	W	W	H	I	8	11	11	11	8	131	78
Gm08.VB.19	Gm08	3380000	3510000	130000	17	W	W	W	W	W	W	I	7	6	7	5	2	3	250
Gm08.VB.20	Gm08	3510000	3570000	60000	6	W	W	W	W	W	H	I	0	3	0	0	0	49	114
Gm08.VB.21	Gm08	3570000	4580000	1010000	124	W	W	W	W	W	W	I	32	64	68	58	46	44	2302
Gm08.VB.22	Gm08	4580000	4610000	30000	2	W	W	S	W	S	S	I	0	0	117	0	103	104	58
Gm08.VB.23	Gm08	4610000	4740000	130000	13	W	W	S	W	S	W	I	9	9	374	9	317	8	322
Gm08.VB.24	Gm08	4740000	5130000	390000	44	W	B	B	B	B	W	I	14	875	895	849	820	14	765
Gm08.VB.25	Gm08	5130000	5650000	520000	66	W	W	W	W	W	W	I	21	43	39	42	46	24	1101
Gm08.VB.26	Gm08	5650000	5740000	90000	12	W	B	B	B	B	W	I	7	229	229	227	208	7	115
Gm08.VB.27	Gm08	5740000	5980000	240000	27	W	W	W	W	W	W	I	10	21	19	17	17	13	425
Gm08.VB.28	Gm08	5980000	6420000	440000	59	W	B	B	B	B	W	I	39	1992	2046	1945	1778	31	1011
Gm08.VB.29	Gm08	6420000	6540000	120000	8	W	W	W	W	W	W	I	22	28	30	25	33	27	160
Gm08.VB.30	Gm08	6540000	6630000	90000	10	W	B	B	B	B	W	I	7	257	253	255	233	5	200
Gm08.VB.31	Gm08	6630000	7360000	730000	81	W	W	W	W	W	W	I	51	98	79	70	54	44	1152
Gm08.VB.32	Gm08	7360000	7450000	90000	11	W	W	W	W	W	H	I	3	10	3	3	4	283	155
Gm08.VB.33	Gm08	7450000	7500000	50000	5	W	B	W	W	W	H	I	9	259	8	8	8	48	130
Gm08.VB.34	Gm08	7500000	7530000	30000	4	W	B	W	W	W	W	I	0	190	0	0	0	0	120
Gm08.VB.35	Gm08	7530000	7810000	280000	24	W	W	W	W	W	W	I	11	23	11	11	8	17	545
Gm08.VB.36	Gm08	7810000	8270000	460000	55	W	B	W	W	W	W	I	12	1079	14	12	11	23	1007
Gm08.VB.37	Gm08	8270000	8280000	10000	1	W	W	W	W	W	W	I	1	2	1	0	1	1	15
Gm08.VB.38	Gm08	8280000	8580000	300000	35	W	W	W	W	W	H	I	4	12	2	4	2	611	415
Gm08.VB.39	Gm08	8580000	8790000	210000	27	W	W	W	W	W	W	I	17	18	17	19	15	25	418
Gm08.VB.40	Gm08	8790000	8920000	130000	12	W	W	W	W	W	H	I	3	8	3	3	0	87	241
Gm08.VB.41	Gm08	8920000	9100000	180000	18	W	W	W	W	W	W	I	38	36	34	26	10	21	393
Gm08.VB.42	Gm08	9100000	9170000	70000	7	W	B	W	W	W	W	I	6	246	6	5	6	6	215
Gm08.VB.43	Gm08	9170000	9180000	10000	1	W	W	W	W	W	W	I	0	0	0	0	0	1	37
Gm08.VB.44	Gm08	9180000	9400000	220000	33	W	W	W	W	W	H	I	16	22	19	14	7	372	518
Gm08.VB.45	Gm08	9400000	10040000	640000	74	W	B	W	W	W	H	I	35	2068	35	35	32	1822	1252
Gm08.VB.46	Gm08	10040000	10270000	230000	34	W	W	W	W	W	W	I	3	21	3	3	0	15	449
Gm08.VB.47	Gm08	10270000	10340000	70000	4	W	B	W	W	W	W	I	22	213	22	12	16	20	155
Gm08.VB.48	Gm08	10340000	11070000	730000	94	W	B	W	W	W	H	I	12	1684	13	10	10	1164	1247

Gm08.VB.49	Gm08	11070000	11230000	160000	18	W	B	W	W	W	W	I	3	637	3	3	1	11	277
Gm08.VB.50	Gm08	11230000	11240000	10000	1	W	B	W	W	W	H	I	0	37	0	0	0	31	14
Gm08.VB.51	Gm08	11240000	11440000	200000	18	W	B	W	W	W	W	I	2	588	2	2	0	15	420
Gm08.VB.52	Gm08	11440000	11600000	160000	14	W	B	W	W	W	H	I	6	415	7	7	3	308	425
Gm08.VB.53	Gm08	11600000	11900000	300000	26	W	W	W	W	W	W	I	15	20	14	13	15	17	397
Gm08.VB.54	Gm08	11900000	11980000	80000	5	W	B	W	W	W	H	I	0	388	0	0	0	200	118
Gm08.VB.55	Gm08	11980000	11990000	10000	2	W	B	W	W	W	W	W	0	12	0	0	0	4	1
Gm08.VB.56	Gm08	11990000	12330000	340000	32	W	W	W	W	W	W	I	14	33	16	16	16	22	380
Gm08.VB.57	Gm08	12330000	12430000	100000	11	W	B	W	W	W	W	I	8	106	8	7	7	29	159
Gm08.VB.58	Gm08	12430000	12560000	130000	15	W	B	W	W	W	B	I	2	137	2	2	0	118	322
Gm08.VB.59	Gm08	12560000	13910000	1350000	108	W	W	W	W	W	W	I	168	209	176	151	131	136	2196
Gm08.VB.60	Gm08	13910000	13940000	30000	1	W	B	W	W	W	W	I	0	36	0	0	0	3	32
Gm08.VB.61	Gm08	13940000	14040000	100000	11	W	B	W	W	W	H	I	2	371	2	3	2	111	215
Gm08.VB.62	Gm08	14040000	14210000	170000	13	W	W	W	W	W	W	I	19	30	20	20	19	25	402
Gm08.VB.63	Gm08	14210000	14360000	150000	16	W	B	W	W	W	H	I	26	468	29	28	18	160	414
Gm08.VB.64	Gm08	14360000	14400000	40000	6	W	B	W	W	W	W	I	0	37	0	0	0	4	58
Gm08.VB.65	Gm08	14400000	14610000	210000	20	W	W	W	W	W	W	I	34	50	31	33	30	45	588
Gm08.VB.66	Gm08	14610000	14860000	250000	26	W	B	W	W	W	B	I	13	582	13	11	11	522	480
Gm08.VB.67	Gm08	14860000	15150000	290000	32	W	W	W	W	W	W	I	10	22	12	8	11	17	478
Gm08.VB.68	Gm08	15150000	15300000	150000	15	W	B	W	W	W	H	I	5	615	5	5	7	285	304
Gm08.VB.69	Gm08	15300000	15320000	20000	2	W	B	W	W	W	W	I	0	21	0	0	0	3	50
Gm08.VB.70	Gm08	15320000	15570000	250000	27	W	W	W	W	W	W	I	6	23	6	8	6	13	687
Gm08.VB.71	Gm08	15570000	15580000	10000	0	W	B	W	W	W	W	I	0	33	0	0	0	4	22
Gm08.VB.72	Gm08	15580000	16260000	680000	62	W	B	W	W	W	H	I	73	2533	78	74	60	400	1477
Gm08.VB.73	Gm08	16260000	16280000	20000	1	W	B	W	W	W	W	I	3	44	4	3	0	9	23
Gm08.VB.74	Gm08	16280000	16460000	180000	17	W	W	W	W	W	W	I	9	20	10	10	11	21	365
Gm08.VB.75	Gm08	16460000	16780000	320000	41	W	W	W	W	W	H	I	19	18	23	20	9	393	542
Gm08.VB.76	Gm08	16780000	17220000	440000	53	W	W	W	W	W	W	I	25	20	23	20	19	25	1266
Gm08.VB.77	Gm08	17220000	17360000	140000	14	W	W	W	W	W	H	I	12	13	14	13	7	209	242
Gm08.VB.78	Gm08	17360000	17550000	190000	14	W	W	W	W	W	W	I	4	2	4	5	1	7	614
Gm08.VB.79	Gm08	17550000	17630000	80000	7	W	W	W	W	W	H	I	13	11	13	11	10	90	170
Gm08.VB.80	Gm08	17630000	18170000	540000	61	W	W	W	W	W	W	I	22	21	23	19	20	30	1323
Gm08.VB.81	Gm08	18170000	18450000	280000	28	W	W	W	W	W	H	I	19	21	21	21	15	908	1168
Gm08.VB.82	Gm08	18450000	18610000	160000	10	W	W	W	W	W	W	W	0	0	0	0	0	5	56
Gm08.VB.83	Gm08	18610000	18950000	340000	24	W	W	W	W	W	H	I	52	53	52	46	38	1226	1227
Gm08.VB.84	Gm08	18950000	19060000	110000	10	W	W	W	W	W	W	I	0	0	1	0	0	4	299
Gm08.VB.85	Gm08	19060000	19100000	40000	1	W	W	W	W	W	H	I	3	3	6	1	11	27	92
Gm08.VB.86	Gm08	19100000	19360000	260000	11	W	B	W	W	W	H	I	34	1351	33	22	25	759	794
Gm08.VB.87	Gm08	19360000	20340000	980000	40	W	B	W	W	W	W	I	48	2452	48	47	32	53	1932
Gm08.VB.88	Gm08	20340000	20480000	140000	12	W	W	W	W	W	W	I	0	6	0	0	0	1	361
Gm08.VB.89	Gm08	20480000	20510000	30000	4	W	W	W	W	W	H	I	0	0	0	0	0	69	52
Gm08.VB.90	Gm08	20510000	20830000	320000	22	W	W	W	W	W	W	I	5	12	5	5	4	7	1267
Gm08.VB.91	Gm08	20830000	21160000	330000	13	W	B	W	W	W	W	I	24	358	23	18	20	48	277
Gm08.VB.92	Gm08	21160000	22090000	930000	52	W	B	W	W	W	H	I	44	3238	49	42	38	2761	3842
Gm08.VB.93	Gm08	22090000	22490000	400000	24	W	W	W	W	W	W	I	10	34	11	10	10	28	1625
Gm08.VB.94	Gm08	22490000	22650000	160000	8	W	B	W	W	W	B	I	53	1217	49	38	34	1187	1043
Gm08.VB.95	Gm08	22650000	22710000	60000	4	W	W	W	W	W	W	I	2	13	2	3	2	11	155
Gm08.VB.96	Gm08	22710000	22890000	180000	9	W	W	W	W	W	W	I	6	26	6	6	1	18	123
Gm08.VB.97	Gm08	22890000	22900000	10000	0	W	B	W	W	W	W	I	0	5	0	1	0	4	34
Gm08.VB.98	Gm08	22900000	23300000	400000	20	W	B	W	W	W	B	I	55	2433	56	52	44	2380	2234
Gm08.VB.99	Gm08	23300000	35340000	12040000	97	W	B	W	W	W	W	I	607	23340	619	532	600	953	5530
Gm08.VB.100	Gm08	35340000	35460000	120000	2	W	W	W	W	W	W	I	3	14	2	3	2	3	188
Gm08.VB.101	Gm08	35460000	36420000	960000	19	W	B	W	W	W	W	I	38	3844	35	31	20	52	713
Gm08.VB.102	Gm08	36420000	39960000	3540000	127	W	W	W	W	W	W	I	180	298	180	168	144	240	9253
Gm08.VB.103	Gm08	39960000	39980000	20000	2	W	B	W	W	W	B	I	0	52	0	0	0	52	38
Gm08.VB.104	Gm08	39980000	40290000	310000	16	W	W	W	W	W	W	I	22	24	20	22	15	19	1125
Gm08.VB.105	Gm08	40290000	40510000	220000	15	W	W	W	W	W	H	I	5	6	5	6	5	179	582
Gm08.VB.106	Gm08	40510000	40670000	160000	8	W	W	W	W	W	W	I	9	10	10	9	7	24	334
Gm08.VB.107	Gm08	40670000	40810000	140000	6	W	W	W	W	W	H	I	2	3	2	4	2	131	520
Gm08.VB.108	Gm08	40810000	41110000	300000	11	W	W	W	W	W	W	I	55	53	54	50	27	60	1044
Gm08.VB.109	Gm08	41110000	41530000	420000	30	W	W	W	W	W	H	I	8	8	8	8	5	1240	1470
Gm08.VB.110	Gm08	41530000	41770000	240000	17	W	W	W	W	W	W	I	14	16	13	17	10	18	1009
Gm08.VB.111	Gm08	41770000	41870000	100000	10	W	W	W	W	W	H	I	15	14	13	10	12	472	292
Gm08.VB.112	Gm08	41870000	41980000	110000	8	W	W	W	W	W	W	I	16	16	18	17	12	12	292
Gm08.VB.113	Gm08	41980000	42070000	90000	5	W	W	W	W	W	H	I	19	19	24	19	22	407	208
Gm08.VB.114	Gm08	42070000	42510000	440000	29	W	W	W	W	W	W	I	84	85	79	78	77	90	1403
Gm08.VB.115	Gm08	42510000	43580000	1070000	97	W	W	W	W	W	H	I	102	99	108	98	82	4767	3140
Gm08.VB.116	Gm08	43580000	43770000	190000	15	W	W	W	W	W	W	I	16	15	12	17	4	13	840
Gm08.VB.117	Gm08	43770000	44010000	240000	23	W	W	W	W	W	H	I	4	5	5	5	6	579	536
Gm08.VB.118	Gm08	44010000	44210000	200000	26	W	W	W	W	W	W	I	15	16	15	14	14	16	424
Gm08.VB.119	Gm08	44210000	44740000	530000	52	W	W	W	W	W	H	I	11	11	11	11	9	1818	1226
Gm08.VB.120	Gm08	44740000	45210000	470000	51	W	W	W	W	W	W	I	22	25	24	23	12	44	1262
Gm08.VB.121	Gm08	45210000	45490000	280000	32	W	W	W	W	W	H	I	14	14	15	14	13	552	534
Gm08.VB.122	Gm08	45490000	45620000	130000	14	W	W	W	W	W	W	I	5	5	5	5	5	9	127
Gm08.VB.123	Gm08	45620000	45980000	360000	38	W	W	W	W	W	H	I	9	13	14	13	9	274	896
Gm08.VB.124	Gm08	45980000	46550000	570000	60	W	W	W	W	W	W	I	62	60	63	56	48	83	1335
Gm08.VB.125	Gm08	46550000	46990000	440000	43	W	W	W	W	W	H	I	38	39	38	37	35	270	896
Gm08.VB.126	Gm08	46990000	46995532	5532	0	W	W	W	W	W	W	I	1	2	1	2	2	1	7

Gm09.VB.1	Gm09	0	210000	210000	19	W	W	W	W	W	W	W	21	25	62	25	23	20	62
Gm09.VB.2	Gm09	210000	230000	20000	3	W	W	S	W	W	W	I	0	0	38	0	0	0	55
Gm09.VB.3	Gm09	230000	320000	90000	9	W	W	W	W	W	W	I	0	3	0	3	2	0	186
Gm09.VB.4	Gm09	320000	450000	130000	18	W	B	W	B	D	H	I	7	361	7	349	278	398	331
Gm09.VB.5	Gm09	450000	620000	170000	19	W	B	W	B	B	W	I	17	396	18	384	369	18	408
Gm09.VB.6	Gm09	620000	720000	100000	10	W	W	W	W	W	H	I	3	13	4	14	13	175	168
Gm09.VB.7	Gm09	720000	770000	50000	5	W	B	W	B	B	H	I	2	84	2	83	77	150	127
Gm09.VB.8	Gm09	770000	880000	110000	15	W	W	W	W	W	H	I	1	4	2	4	4	586	179
Gm09.VB.9	Gm09	880000	930000	50000	5	W	B	W	B	B	H	I	7	164	7	164	161	208	156
Gm09.VB.10	Gm09	930000	940000	10000	1	W	B	W	B	W	H	W	0	5	0	5	4	37	2
Gm09.VB.11	Gm09	940000	1040000	100000	15	W	W	W	W	W	H	I	3	16	6	20	31	228	139
Gm09.VB.12	Gm09	1040000	1210000	170000	19	W	B	W	B	B	H	I	13	555	14	540	522	339	455
Gm09.VB.13	Gm09	1210000	1540000	330000	40	W	B	W	B	B	W	I	4	1043	3	1033	975	19	443
Gm09.VB.14	Gm09	1540000	1890000	350000	30	W	B	W	B	B	B	I	18	1729	19	1680	1509	1573	733
Gm09.VB.15	Gm09	1890000	2630000	740000	76	W	W	W	W	W	W	I	19	32	22	29	21	22	1741
Gm09.VB.16	Gm09	2630000	2950000	320000	29	W	B	W	B	B	B	I	16	956	16	933	855	889	967
Gm09.VB.17	Gm09	2950000	3090000	140000	13	W	W	W	W	W	W	I	0	6	1	6	6	6	217
Gm09.VB.18	Gm09	3090000	3140000	50000	3	W	B	W	B	B	W	I	1	58	1	55	49	4	37
Gm09.VB.19	Gm09	3140000	3180000	40000	6	W	B	W	B	B	H	W	0	21	0	21	21	104	16
Gm09.VB.20	Gm09	3180000	3200000	20000	1	W	B	W	B	B	W	I	0	84	0	79	71	0	49
Gm09.VB.21	Gm09	3200000	3580000	380000	34	W	W	W	W	W	W	I	28	29	32	25	21	30	916
Gm09.VB.22	Gm09	3580000	3710000	130000	15	W	W	W	W	W	H	I	3	3	1	3	3	79	380
Gm09.VB.23	Gm09	3710000	3880000	170000	18	W	B	W	B	B	H	I	17	836	17	820	741	385	334
Gm09.VB.24	Gm09	3880000	3960000	80000	6	W	B	W	B	B	W	I	2	570	2	567	518	4	235
Gm09.VB.25	Gm09	3960000	4090000	130000	9	W	W	W	W	W	W	I	3	13	3	14	12	2	250
Gm09.VB.26	Gm09	4090000	4250000	160000	13	W	B	W	B	B	W	I	6	1454	8	1449	1379	8	689
Gm09.VB.27	Gm09	4250000	4670000	420000	32	W	B	W	B	B	H	I	58	2066	59	2047	1881	657	1420
Gm09.VB.28	Gm09	4670000	5000000	330000	21	W	B	W	B	B	W	I	22	2242	26	2220	2041	36	1224
Gm09.VB.29	Gm09	5000000	5330000	330000	22	W	W	W	W	W	H	I	11	27	12	27	27	1875	1352
Gm09.VB.30	Gm09	5330000	5350000	20000	3	W	B	W	B	B	H	I	0	31	0	31	31	42	71
Gm09.VB.31	Gm09	5350000	5590000	240000	15	W	W	W	W	W	H	I	5	11	6	13	18	829	642
Gm09.VB.32	Gm09	5590000	5610000	20000	0	W	W	W	W	W	W	W	0	3	0	3	3	2	8
Gm09.VB.33	Gm09	5610000	5740000	130000	7	W	B	W	B	B	W	I	7	632	7	627	588	13	604
Gm09.VB.34	Gm09	5740000	5830000	90000	6	W	B	W	B	B	H	I	3	403	3	390	400	391	246
Gm09.VB.35	Gm09	5830000	5940000	110000	9	W	W	W	W	W	H	I	0	1	0	0	0	357	253
Gm09.VB.36	Gm09	5940000	6060000	120000	7	W	B	W	B	B	H	I	7	269	7	251	232	250	304
Gm09.VB.37	Gm09	6060000	6080000	20000	0	W	B	W	B	B	W	I	0	211	0	205	192	1	99
Gm09.VB.38	Gm09	6080000	6180000	100000	9	W	W	W	W	W	W	I	15	14	14	11	12	9	267
Gm09.VB.39	Gm09	6180000	6190000	10000	1	W	B	W	B	B	W	I	0	22	0	22	20	0	17
Gm09.VB.40	Gm09	6190000	6310000	120000	5	W	B	W	B	B	H	I	6	722	6	702	701	121	422
Gm09.VB.41	Gm09	6310000	6910000	600000	32	W	B	W	B	B	W	I	17	1787	19	1761	1758	24	1542
Gm09.VB.42	Gm09	6910000	7060000	150000	6	W	B	W	W	B	W	I	4	343	5	4	332	6	407
Gm09.VB.43	Gm09	7060000	7170000	110000	9	W	B	W	W	B	H	I	1	279	1	1	276	78	639
Gm09.VB.44	Gm09	7170000	7200000	30000	2	W	B	W	W	D	W	I	4	69	7	8	43	2	106
Gm09.VB.45	Gm09	7200000	7320000	120000	9	W	W	W	W	W	W	I	5	17	3	3	21	2	359
Gm09.VB.46	Gm09	7320000	7390000	70000	4	W	B	W	W	B	W	I	1	635	1	1	615	2	379
Gm09.VB.47	Gm09	7390000	8020000	630000	22	W	B	W	W	B	H	I	47	2619	49	50	2512	1297	2015
Gm09.VB.48	Gm09	8020000	8150000	130000	3	W	B	W	W	B	W	I	2	801	2	2	778	20	689
Gm09.VB.49	Gm09	8150000	8680000	530000	17	W	B	W	W	B	H	I	33	2503	34	23	2448	348	2796
Gm09.VB.50	Gm09	8680000	8790000	110000	3	W	B	W	W	B	W	I	1	71	1	0	76	10	308
Gm09.VB.51	Gm09	8790000	9010000	220000	3	W	B	W	W	B	H	I	18	1196	16	14	1195	216	894
Gm09.VB.52	Gm09	9010000	9590000	580000	13	W	B	W	W	B	W	I	20	2037	21	21	2043	76	1251
Gm09.VB.53	Gm09	9590000	9620000	30000	1	W	B	W	W	B	H	I	4	349	4	4	343	250	34
Gm09.VB.54	Gm09	9620000	10270000	650000	18	W	B	W	W	B	W	I	38	2505	43	41	2468	74	1076
Gm09.VB.55	Gm09	10270000	10640000	370000	13	W	B	W	W	B	H	I	21	2853	21	22	2692	586	649
Gm09.VB.56	Gm09	10640000	11390000	750000	9	W	B	W	W	B	W	I	36	1911	42	37	1897	110	554
Gm09.VB.57	Gm09	11390000	11710000	320000	8	W	B	W	W	B	H	I	21	327	22	20	308	298	935
Gm09.VB.58	Gm09	11710000	12300000	590000	19	W	B	W	W	B	W	I	37	2324	34	36	2288	52	1173
Gm09.VB.59	Gm09	12300000	13060000	760000	18	W	B	W	W	B	H	I	41	4254	39	42	4191	1928	1453
Gm09.VB.60	Gm09	13060000	13820000	760000	9	W	B	W	W	B	W	I	58	2604	57	67	2611	129	2146
Gm09.VB.61	Gm09	13820000	14420000	600000	5	W	B	W	W	B	H	I	4	1551	7	7	1563	279	1257
Gm09.VB.62	Gm09	14420000	17440000	3020000	31	W	B	W	W	B	W	I	94	6403	99	87	6522	960	6895
Gm09.VB.63	Gm09	17440000	17580000	140000	1	W	B	W	W	B	H	I	1	530	3	1	525	212	308
Gm09.VB.64	Gm09	17580000	17680000	100000	3	W	B	W	W	B	W	I	15	133	14	9	125	29	113
Gm09.VB.65	Gm09	17680000	17780000	100000	0	W	W	W	W	W	W	I	8	28	8	7	26	13	61
Gm09.VB.66	Gm09	17780000	18450000	670000	12	W	B	W	W	B	W	I	32	2858	34	22	2867	109	1946
Gm09.VB.67	Gm09	18450000	18670000	220000	8	W	B	W	W	B	H	I	12	453	12	14	433	437	501
Gm09.VB.68	Gm09	18670000	20130000	1460000	22	W	B	W	W	B	W	I	72	4025	68	68	4052	241	1099
Gm09.VB.69	Gm09	20130000	20910000	780000	15	W	B	W	W	B	H	I	43	3002	39	34	2917	1245	1661
Gm09.VB.70	Gm09	20910000	21090000	180000	6	W	B	W	W	B	W	I	5	874	5	7	862	15	330
Gm09.VB.71	Gm09	21090000	25910000	4820000	43	W	W	W	W	W	W	I	390	677	399	387	657	614	7514
Gm09.VB.72	Gm09	25910000	26320000	410000	15	W	B	W	W	B	W	I	35	1741	33	32	1674	45	838
Gm09.VB.73	Gm09	26320000	26450000	130000	1	W	W	W	W	W	W	I	2	17	3	1	9	3	1058
Gm09.VB.74	Gm09	26450000	29630000	3180000	48	W	B	W	W	B	W	I	110	8656	87	85	8627	253	2002
Gm09.VB.75	Gm09	29630000	30130000	500000	16	W	B	W	W	B	H	I	36	2869	39	35	2713	471	1412
Gm09.VB.76	Gm09	30130000	30280000	150000	7	W	W	W	W	W	W	I	8	14	7	5	28	63	688
Gm09.VB.77	Gm09	30280000	30870000	590000	19	W	W	W	W	W	W	I	44	76	64	40	83	96	2034
Gm09.VB.78	Gm09	30870000	30900000	30000	1	W	B	W	W	B	W	I	0	78	1	1	70	4	99

Gm09.VB.79	Gm09	30900000	30960000	60000	2	W	W	W	W	W	H	I	10	13	10	8	7	389	302
Gm09.VB.80	Gm09	30960000	33810000	2850000	88	W	W	W	W	W	W	I	148	221	165	152	213	210	8358
Gm09.VB.81	Gm09	33810000	34260000	450000	20	W	W	W	W	W	H	I	62	55	51	43	52	480	1108
Gm09.VB.82	Gm09	34260000	34310000	50000	0	W	W	W	W	W	W	I	1	13	1	2	13	14	257
Gm09.VB.83	Gm09	34310000	35050000	740000	34	W	B	W	W	B	W	I	27	4449	27	28	4345	56	1463
Gm09.VB.84	Gm09	35050000	35160000	110000	6	W	W	W	W	W	W	I	2	8	2	2	7	3	431
Gm09.VB.85	Gm09	35160000	35190000	30000	3	W	B	W	W	D	H	I	8	174	8	8	114	112	58
Gm09.VB.86	Gm09	35190000	35200000	10000	1	W	W	W	W	W	H	I	0	0	0	0	0	5	29
Gm09.VB.87	Gm09	35200000	35290000	90000	7	W	W	W	W	W	W	I	1	2	1	1	3	2	316
Gm09.VB.88	Gm09	35290000	35770000	480000	33	W	B	W	W	B	W	I	22	2859	20	19	2671	25	2142
Gm09.VB.89	Gm09	35770000	36480000	710000	61	W	W	W	W	W	W	I	22	42	27	25	35	28	2227
Gm09.VB.90	Gm09	36480000	36530000	50000	3	W	W	W	W	W	H	I	13	14	12	12	8	63	89
Gm09.VB.91	Gm09	36530000	36590000	60000	2	W	B	W	W	B	W	I	17	119	16	16	112	36	63
Gm09.VB.92	Gm09	36590000	36720000	130000	6	W	B	W	W	B	W	I	36	519	33	33	461	28	198
Gm09.VB.93	Gm09	36720000	37110000	390000	30	W	B	W	W	B	H	I	40	1373	39	38	1238	732	1063
Gm09.VB.94	Gm09	37110000	37220000	110000	10	W	B	S	S	B	B	I	10	276	576	563	243	256	183
Gm09.VB.95	Gm09	37220000	37650000	430000	31	W	B	W	W	B	B	I	37	1892	48	49	1692	1634	1143
Gm09.VB.96	Gm09	37650000	37950000	300000	22	W	B	B	B	B	B	I	8	1095	1151	1097	1030	966	767
Gm09.VB.97	Gm09	37950000	38330000	380000	30	W	W	W	W	W	H	I	6	19	14	17	13	176	1139
Gm09.VB.98	Gm09	38330000	38350000	20000	1	W	B	W	W	B	B	I	6	62	8	8	47	41	11
Gm09.VB.99	Gm09	38350000	38360000	10000	0	W	W	S	S	W	W	I	0	2	26	26	2	2	23
Gm09.VB.100	Gm09	38360000	38630000	270000	31	W	B	W	W	B	H	I	2	356	21	19	330	230	573
Gm09.VB.101	Gm09	38630000	38720000	90000	6	W	B	B	B	B	B	I	12	430	427	421	379	346	223
Gm09.VB.102	Gm09	38720000	38840000	120000	12	W	W	S	S	W	H	I	4	12	526	523	13	492	347
Gm09.VB.103	Gm09	38840000	38990000	150000	13	W	B	S	S	B	B	I	6	658	772	739	593	618	421
Gm09.VB.104	Gm09	38990000	39090000	100000	11	W	B	S	S	B	W	I	0	311	395	388	286	4	295
Gm09.VB.105	Gm09	39090000	39210000	120000	12	W	W	S	S	W	W	I	6	14	122	117	6	1	98
Gm09.VB.106	Gm09	39210000	39220000	10000	1	W	B	S	S	B	W	I	0	27	5	5	27	0	17
Gm09.VB.107	Gm09	39220000	39410000	190000	14	W	B	W	W	B	W	I	14	905	22	20	850	9	554
Gm09.VB.108	Gm09	39410000	39540000	130000	10	W	B	S	S	B	W	I	10	1010	626	613	943	2	293
Gm09.VB.109	Gm09	39540000	39780000	240000	23	W	B	W	W	B	W	I	3	1039	12	12	983	3	564
Gm09.VB.110	Gm09	39780000	39950000	170000	16	W	B	W	W	B	H	I	9	645	16	14	590	728	380
Gm09.VB.111	Gm09	39950000	40050000	100000	12	W	W	W	W	W	H	I	0	5	2	2	5	157	204
Gm09.VB.112	Gm09	40050000	40110000	60000	2	W	W	W	W	W	W	I	24	29	21	17	22	12	93
Gm09.VB.113	Gm09	40110000	40150000	40000	5	W	B	W	W	B	W	W	0	18	1	1	17	1	7
Gm09.VB.114	Gm09	40150000	42280000	2130000	239	W	B	B	B	B	H	I	112	9449	8942	8915	8661	8900	5548
Gm09.VB.115	Gm09	42280000	42630000	350000	39	W	B	W	B	B	H	I	8	1228	16	1220	1071	934	472
Gm09.VB.116	Gm09	42630000	43220000	590000	55	W	B	S	B	B	H	I	50	2322	2278	2284	2124	1588	1055
Gm09.VB.117	Gm09	43220000	43500000	280000	23	W	B	W	B	B	H	I	18	1044	22	1034	988	735	580
Gm09.VB.118	Gm09	43500000	43600000	100000	14	W	W	W	W	W	H	I	0	10	0	10	10	56	114
Gm09.VB.119	Gm09	43600000	43640000	40000	5	W	B	W	B	B	H	I	2	166	1	157	151	70	88
Gm09.VB.120	Gm09	43640000	45090000	1450000	161	W	B	W	B	B	W	I	99	3128	109	3080	2835	98	2412
Gm09.VB.121	Gm09	45090000	45220000	130000	8	W	W	W	W	W	W	I	3	13	2	12	12	5	403
Gm09.VB.122	Gm09	45220000	46660000	1440000	144	W	B	W	B	B	W	I	166	3121	163	3083	2806	149	2992
Gm09.VB.123	Gm09	46660000	46840000	180000	20	W	B	W	B	B	B	I	6	245	6	244	231	235	437
Gm09.VB.124	Gm09	46840000	46843750	3750	0	W	B	W	G	G	G	I	0	3	0	2	2	2	6
Gm10.VB.1	Gm10	0	470000	470000	41	W	B	W	W	W	H	I	12	813	14	14	10	628	840
Gm10.VB.2	Gm10	470000	1030000	560000	56	W	W	W	W	W	H	I	39	58	38	39	32	413	751
Gm10.VB.3	Gm10	1030000	1080000	50000	5	W	B	W	W	W	W	I	0	79	0	0	0	2	138
Gm10.VB.4	Gm10	1080000	1180000	100000	13	W	W	W	W	W	W	I	1	4	1	1	2	1	360
Gm10.VB.5	Gm10	1180000	1720000	540000	63	W	B	W	W	W	W	I	30	1165	32	27	23	25	1061
Gm10.VB.6	Gm10	1720000	1870000	150000	14	W	W	W	W	W	W	I	8	18	7	7	6	6	153
Gm10.VB.7	Gm10	1870000	2000000	130000	13	W	B	W	W	W	W	I	0	240	1	1	0	3	226
Gm10.VB.8	Gm10	2000000	2020000	20000	3	W	B	W	W	W	H	I	1	36	1	1	1	21	25
Gm10.VB.9	Gm10	2020000	2180000	160000	18	W	W	W	W	W	W	I	3	13	3	3	2	59	228
Gm10.VB.10	Gm10	2180000	2240000	60000	7	W	W	W	W	W	W	I	0	4	0	0	0	1	142
Gm10.VB.11	Gm10	2240000	2330000	90000	8	W	B	W	W	W	W	I	4	270	4	3	3	9	205
Gm10.VB.12	Gm10	2330000	2530000	200000	20	W	B	W	W	W	H	I	6	633	6	6	5	527	447
Gm10.VB.13	Gm10	2530000	2720000	190000	18	W	W	W	W	W	W	I	3	22	3	3	3	14	544
Gm10.VB.14	Gm10	2720000	2930000	210000	21	W	B	W	W	W	H	I	0	586	0	0	0	311	344
Gm10.VB.15	Gm10	2930000	3360000	430000	31	W	B	W	W	W	W	I	72	1384	74	67	46	61	940
Gm10.VB.16	Gm10	3360000	3470000	110000	12	W	B	W	W	W	H	I	9	294	9	9	9	194	283
Gm10.VB.17	Gm10	3470000	3500000	30000	4	W	B	W	W	W	W	I	0	72	0	0	0	0	106
Gm10.VB.18	Gm10	3500000	3760000	260000	27	W	W	W	W	W	W	I	34	42	33	32	30	36	443
Gm10.VB.19	Gm10	3760000	3850000	90000	9	W	W	W	W	W	W	I	5	7	5	5	3	20	247
Gm10.VB.20	Gm10	3850000	4220000	370000	39	W	B	W	W	W	H	I	25	1863	21	20	21	1587	1274
Gm10.VB.21	Gm10	4220000	4260000	40000	3	W	B	W	B	W	H	I	1	173	2	168	1	107	107
Gm10.VB.22	Gm10	4260000	4380000	120000	10	W	W	W	W	W	W	I	6	11	6	10	7	11	369
Gm10.VB.23	Gm10	4380000	4450000	70000	7	W	B	W	B	W	B	I	6	265	8	260	4	234	238
Gm10.VB.24	Gm10	4450000	4780000	330000	27	W	B	W	W	W	B	I	11	1284	11	11	10	1213	1090
Gm10.VB.25	Gm10	4780000	4960000	180000	14	W	W	W	W	W	W	I	7	10	7	6	6	15	541
Gm10.VB.26	Gm10	4960000	5140000	180000	11	W	W	W	W	W	H	I	10	16	6	7	1	170	485
Gm10.VB.27	Gm10	5140000	5230000	90000	10	W	W	W	W	W	W	I	22	26	20	17	13	22	163
Gm10.VB.28	Gm10	5230000	5240000	10000	1	W	B	W	W	W	W	I	0	31	0	0	0	1	23
Gm10.VB.29	Gm10	5240000	5480000	240000	18	W	B	W	W	W	H	I	6	1082	6	7	6	385	534
Gm10.VB.30	Gm10	5480000	5570000	90000	6	W	B	W	W	B	H	I	10	382	8	7	370	266	262
Gm10.VB.31	Gm10	5570000	5630000	60000	3	W	B	W	W	B	W	I	1	121	1	0	106	7	158
Gm10.VB.32	Gm10	5630000	5900000	270000	12	W	W	W	W	W	W	I	11	38	11	12	37	34	536

Gm10.VB.33	Gm10	5900000	6010000	110000	12	W	B	W	W	B	W	I	4	144	8	8	144	11	502
Gm10.VB.34	Gm10	6010000	6020000	10000	0	W	B	W	B	D	W	I	0	14	0	15	10	5	7
Gm10.VB.35	Gm10	6020000	6030000	10000	2	W	B	W	B	B	H	I	3	31	2	30	28	8	26
Gm10.VB.36	Gm10	6030000	6140000	110000	7	W	W	W	W	W	H	I	4	12	14	13	9	75	440
Gm10.VB.37	Gm10	6140000	6510000	370000	23	W	W	S	S	W	H	I	47	62	1205	1167	47	304	1071
Gm10.VB.38	Gm10	6510000	6610000	100000	5	W	B	S	S	B	H	I	8	308	413	426	292	237	300
Gm10.VB.39	Gm10	6610000	6670000	60000	3	W	B	W	W	B	W	I	30	319	27	26	272	14	39
Gm10.VB.40	Gm10	6670000	7090000	420000	23	W	W	W	W	W	W	I	14	45	15	15	43	26	1403
Gm10.VB.41	Gm10	7090000	7130000	40000	3	W	B	W	W	B	H	I	2	262	2	2	263	20	193
Gm10.VB.42	Gm10	7130000	7310000	180000	15	W	W	W	W	W	W	I	7	18	7	9	10	7	424
Gm10.VB.43	Gm10	7310000	7340000	30000	1	W	B	W	B	B	W	I	1	174	3	159	165	9	106
Gm10.VB.44	Gm10	7340000	7470000	130000	6	W	W	W	W	W	W	I	16	20	13	16	20	24	213
Gm10.VB.45	Gm10	7470000	7560000	90000	5	W	B	W	W	B	B	I	0	306	0	0	310	307	65
Gm10.VB.46	Gm10	7560000	7690000	130000	10	W	W	W	W	W	H	I	1	5	1	1	5	64	314
Gm10.VB.47	Gm10	7690000	8670000	980000	23	W	W	W	W	W	W	I	44	73	47	37	74	69	634
Gm10.VB.48	Gm10	8670000	9470000	800000	20	W	B	W	W	B	B	I	27	3717	33	32	3663	3667	3697
Gm10.VB.49	Gm10	9470000	9580000	110000	2	W	W	W	W	W	W	I	0	0	0	0	0	38	120
Gm10.VB.50	Gm10	9580000	9840000	260000	7	W	W	W	W	W	W	I	10	9	10	10	7	24	914
Gm10.VB.51	Gm10	9840000	11130000	1290000	36	W	W	W	W	W	H	I	82	78	80	85	67	828	5102
Gm10.VB.52	Gm10	11130000	11770000	640000	14	W	B	B	B	B	W	I	32	3900	3952	3897	3831	57	1160
Gm10.VB.53	Gm10	11770000	13890000	2120000	45	W	B	B	B	B	H	I	132	9743	9785	9608	9520	6121	6822
Gm10.VB.54	Gm10	13890000	14220000	330000	2	W	B	B	G	D	W	W	1	141	142	140	147	107	62
Gm10.VB.55	Gm10	14220000	16810000	2590000	30	W	B	W	W	B	W	I	131	9809	213	188	9967	344	1406
Gm10.VB.56	Gm10	16810000	17430000	620000	9	W	B	B	B	B	W	I	35	3371	3305	3311	3409	96	265
Gm10.VB.57	Gm10	17430000	19310000	1880000	31	W	B	B	B	B	H	I	75	4908	5034	4928	4981	2200	1394
Gm10.VB.58	Gm10	19310000	21520000	2210000	24	W	B	B	B	B	W	I	110	3367	3380	3335	3296	545	1117
Gm10.VB.59	Gm10	21520000	23210000	1690000	8	W	B	B	B	B	H	I	84	1370	1382	1353	1367	804	1055
Gm10.VB.60	Gm10	23210000	23320000	110000	0	W	W	W	W	W	W	W	4	34	38	43	33	20	25
Gm10.VB.61	Gm10	23320000	24100000	780000	4	W	B	B	B	B	H	I	43	669	667	653	669	406	528
Gm10.VB.62	Gm10	24100000	24240000	140000	0	W	B	B	B	B	W	W	11	70	69	69	73	38	61
Gm10.VB.63	Gm10	24240000	24970000	730000	5	W	B	B	B	B	H	I	31	604	600	579	633	375	451
Gm10.VB.64	Gm10	24970000	25230000	260000	1	W	W	W	W	W	W	W	19	36	37	36	44	46	28
Gm10.VB.65	Gm10	25230000	25270000	40000	0	W	W	S	S	S	W	W	1	17	21	23	22	14	16
Gm10.VB.66	Gm10	25270000	25430000	160000	0	W	W	W	W	W	W	W	11	47	40	45	44	35	31
Gm10.VB.67	Gm10	25430000	25590000	160000	0	W	W	W	W	W	W	W	4	28	43	39	42	42	26
Gm10.VB.68	Gm10	25590000	25600000	10000	0	W	B	W	W	D	H	I	2	6	3	5	6	7	5
Gm10.VB.69	Gm10	25600000	27980000	2380000	22	W	B	B	B	B	H	I	120	1698	1701	1650	1696	2014	1200
Gm10.VB.70	Gm10	27980000	28740000	760000	18	W	B	W	W	B	W	I	33	3420	56	66	3436	98	694
Gm10.VB.71	Gm10	28740000	35400000	6660000	109	W	W	W	W	W	W	I	275	375	390	361	376	361	20111
Gm10.VB.72	Gm10	35400000	37180000	1780000	64	W	B	B	B	B	B	I	56	3857	3864	3796	3675	3691	2727
Gm10.VB.73	Gm10	37180000	37230000	50000	3	W	W	W	W	W	W	I	20	21	27	24	22	22	153
Gm10.VB.74	Gm10	37230000	37530000	300000	24	W	W	W	W	W	W	I	21	39	44	37	31	31	1198
Gm10.VB.75	Gm10	37530000	37620000	90000	6	W	W	W	W	W	H	I	26	25	27	26	27	407	349
Gm10.VB.76	Gm10	37620000	37720000	100000	7	W	W	W	W	W	W	I	5	7	7	7	6	12	341
Gm10.VB.77	Gm10	37720000	38530000	810000	81	W	W	W	W	W	H	I	29	74	57	51	65	2851	2716
Gm10.VB.78	Gm10	38530000	38550000	20000	0	W	B	B	W	B	H	I	1	66	65	1	63	119	62
Gm10.VB.79	Gm10	38550000	38650000	100000	10	W	B	B	W	B	W	I	0	335	329	0	324	6	272
Gm10.VB.80	Gm10	38650000	38970000	320000	32	W	B	B	W	B	B	I	38	1500	1506	35	1384	1420	1128
Gm10.VB.81	Gm10	38970000	39260000	290000	28	W	W	W	W	W	W	I	45	43	45	39	40	43	769
Gm10.VB.82	Gm10	39260000	39270000	10000	1	W	B	B	B	B	B	I	0	21	22	21	22	21	17
Gm10.VB.83	Gm10	39270000	39400000	130000	9	W	W	S	W	S	W	I	3	6	184	6	180	30	274
Gm10.VB.84	Gm10	39400000	39790000	390000	34	W	B	S	B	S	S	I	12	1404	1142	1378	1080	1061	1253
Gm10.VB.85	Gm10	39790000	40070000	280000	27	W	B	S	B	S	W	I	35	747	582	744	536	42	554
Gm10.VB.86	Gm10	40070000	40080000	10000	1	W	B	S	B	S	H	I	6	8	21	8	19	17	12
Gm10.VB.87	Gm10	40080000	40100000	20000	1	W	W	S	W	S	H	I	0	0	100	0	98	66	47
Gm10.VB.88	Gm10	40100000	40230000	130000	13	W	W	S	W	D	W	I	5	15	187	15	202	9	229
Gm10.VB.89	Gm10	40230000	40290000	60000	6	W	B	S	B	S	W	I	0	98	462	98	444	1	96
Gm10.VB.90	Gm10	40290000	40400000	110000	3	W	B	W	B	W	W	I	34	129	46	123	31	24	90
Gm10.VB.91	Gm10	40400000	40410000	10000	0	W	W	S	S	W	W	I	3	7	50	51	3	2	24
Gm10.VB.92	Gm10	40410000	40480000	70000	8	W	B	W	B	W	W	I	0	40	0	40	0	1	44
Gm10.VB.93	Gm10	40480000	40640000	160000	17	W	W	W	W	W	W	I	12	21	20	24	16	27	570
Gm10.VB.94	Gm10	40640000	40660000	20000	2	W	B	W	B	W	B	I	0	85	0	84	0	85	70
Gm10.VB.95	Gm10	40660000	40670000	10000	2	W	W	W	G	D	H	I	2	4	2	8	9	11	42
Gm10.VB.96	Gm10	40670000	41000000	330000	28	W	W	W	W	W	W	I	37	52	39	50	26	51	730
Gm10.VB.97	Gm10	41000000	41030000	30000	3	W	W	W	W	W	H	I	0	2	0	2	0	19	64
Gm10.VB.98	Gm10	41030000	41120000	90000	10	W	B	W	B	W	B	I	1	120	2	117	1	120	122
Gm10.VB.99	Gm10	41120000	41620000	500000	43	W	B	W	B	W	W	I	40	1554	54	1520	37	52	1143
Gm10.VB.100	Gm10	41620000	41870000	250000	25	W	W	W	W	W	W	I	15	25	21	25	12	20	659
Gm10.VB.101	Gm10	41870000	42240000	370000	44	W	B	W	B	W	W	I	5	943	30	918	27	29	666
Gm10.VB.102	Gm10	42240000	42470000	230000	21	W	B	B	B	B	B	I	50	627	724	627	622	620	462
Gm10.VB.103	Gm10	42470000	42660000	190000	15	W	W	W	W	W	W	I	67	78	83	74	73	74	305
Gm10.VB.104	Gm10	42660000	42760000	100000	9	W	B	B	B	B	B	I	4	272	281	271	230	241	109
Gm10.VB.105	Gm10	42760000	42860000	100000	6	W	W	W	W	W	W	I	14	16	17	19	10	13	160
Gm10.VB.106	Gm10	42860000	42870000	10000	1	W	W	S	S	W	S	W	0	3	5	5	4	5	3
Gm10.VB.107	Gm10	42870000	43060000	190000	17	W	B	B	B	B	B	I	34	404	418	403	361	370	408
Gm10.VB.108	Gm10	43060000	43690000	630000	44	W	W	W	W	W	W	I	50	88	91	89	76	76	1054
Gm10.VB.109	Gm10	43690000	44180000	490000	40	W	B	B	B	B	B	I	57	1278	1290	1254	1176	1210	1037
Gm10.VB.110	Gm10	44180000	44770000	590000	61	W	B	W	B	W	B	I	20	1232	20	1233	18	1180	1345

Gm10.VB.111	Gm10	44770000	44940000	170000	23	W	W	W	W	W	W	I	3	8	2	7	3	7	349
Gm10.VB.112	Gm10	44940000	44970000	30000	1	W	B	W	B	W	B	I	7	74	7	76	6	68	56
Gm10.VB.113	Gm10	44970000	45230000	260000	20	W	W	W	W	W	W	I	12	26	11	23	11	20	419
Gm10.VB.114	Gm10	45230000	45250000	20000	2	W	B	W	B	W	B	I	1	83	1	84	1	67	81
Gm10.VB.115	Gm10	45250000	45360000	110000	12	W	W	W	W	W	W	I	3	17	3	15	0	15	305
Gm10.VB.116	Gm10	45360000	45410000	50000	3	W	B	W	B	W	B	I	2	161	2	135	2	130	149
Gm10.VB.117	Gm10	45410000	45620000	210000	26	W	W	W	W	W	W	I	23	25	21	26	11	15	550
Gm10.VB.118	Gm10	45620000	45760000	140000	16	W	B	W	B	W	B	I	0	216	0	214	0	196	270
Gm10.VB.119	Gm10	45760000	45860000	100000	6	W	W	W	W	W	W	I	11	15	10	15	10	15	187
Gm10.VB.120	Gm10	45860000	46390000	530000	56	W	B	W	B	W	B	I	29	1397	31	1345	16	1319	1039
Gm10.VB.121	Gm10	46390000	46910000	520000	51	W	W	W	W	W	W	I	56	74	59	76	37	45	980
Gm10.VB.122	Gm10	46910000	46920000	10000	1	W	W	S	G	W	W	I	3	5	10	41	1	1	24
Gm10.VB.123	Gm10	46920000	47070000	150000	15	W	W	W	W	W	W	I	19	22	20	22	18	17	241
Gm10.VB.124	Gm10	47070000	47110000	40000	4	W	W	W	G	W	W	I	1	1	1	86	1	1	91
Gm10.VB.125	Gm10	47110000	47760000	650000	72	W	B	W	G	W	W	I	51	1039	55	2378	36	40	1415
Gm10.VB.126	Gm10	47760000	48480000	720000	95	W	B	S	S	W	W	I	52	1887	3015	2947	38	42	1574
Gm10.VB.127	Gm10	48480000	48720000	240000	22	W	W	S	S	W	W	I	54	58	1090	1067	35	47	539
Gm10.VB.128	Gm10	48720000	49100000	380000	27	W	W	W	W	W	W	I	39	42	41	39	23	19	1002
Gm10.VB.129	Gm10	49100000	49240000	140000	13	W	B	W	W	W	W	I	1	333	1	1	1	1	240
Gm10.VB.130	Gm10	49240000	49560000	320000	40	W	W	W	W	W	W	I	23	36	23	28	15	15	580
Gm10.VB.131	Gm10	49560000	49750000	190000	20	W	B	W	W	W	W	I	9	498	9	9	7	9	466
Gm10.VB.132	Gm10	49750000	49990000	240000	29	W	W	W	W	W	W	I	18	24	19	16	14	15	517
Gm10.VB.133	Gm10	49990000	50540000	550000	70	W	B	W	W	W	W	I	7	1825	6	5	7	9	1388
Gm10.VB.134	Gm10	50540000	50650000	110000	9	W	W	W	W	W	W	I	4	8	4	5	4	7	71
Gm10.VB.135	Gm10	50650000	50960000	310000	31	W	B	W	W	W	W	I	30	1078	31	26	27	36	500
Gm10.VB.136	Gm10	50960000	50969635	9635	0	W	B	W	W	W	W	I	0	31	0	0	0	0	26
Gm11.VB.1	Gm11	0	220000	220000	30	W	W	W	W	W	H	I	19	13	18	14	14	310	617
Gm11.VB.2	Gm11	220000	430000	210000	25	W	W	W	W	W	W	I	10	4	4	4	2	11	372
Gm11.VB.3	Gm11	430000	720000	290000	37	W	W	W	W	W	H	I	12	13	12	12	12	796	501
Gm11.VB.4	Gm11	720000	940000	220000	33	W	W	W	W	W	W	I	4	4	4	4	3	12	309
Gm11.VB.5	Gm11	940000	1070000	130000	17	W	W	W	W	W	H	I	0	0	0	0	1	60	243
Gm11.VB.6	Gm11	1070000	1200000	130000	14	W	W	W	W	W	W	I	2	2	2	2	2	10	187
Gm11.VB.7	Gm11	1200000	1260000	60000	8	W	W	W	W	W	H	I	0	5	0	0	6	46	100
Gm11.VB.8	Gm11	1260000	1440000	180000	23	W	B	W	W	B	H	I	4	315	1	4	272	200	171
Gm11.VB.9	Gm11	1440000	1600000	160000	18	W	W	W	W	W	W	I	12	24	12	12	25	22	298
Gm11.VB.10	Gm11	1600000	1740000	140000	19	W	B	W	W	B	B	I	4	253	9	3	245	243	133
Gm11.VB.11	Gm11	1740000	1940000	200000	20	W	W	W	W	W	W	I	4	6	8	1	7	78	327
Gm11.VB.12	Gm11	1940000	2100000	160000	20	W	W	W	W	W	W	I	7	5	6	6	4	9	420
Gm11.VB.13	Gm11	2100000	2180000	80000	10	W	W	W	W	W	H	I	20	13	18	11	16	56	188
Gm11.VB.14	Gm11	2180000	2530000	350000	44	W	W	W	W	W	W	I	13	13	12	13	14	15	590
Gm11.VB.15	Gm11	2530000	2650000	120000	16	W	W	W	W	W	H	I	2	2	2	3	1	54	233
Gm11.VB.16	Gm11	2650000	3040000	390000	43	W	W	W	W	W	W	I	18	18	18	16	13	37	754
Gm11.VB.17	Gm11	3040000	3120000	80000	10	W	W	W	W	W	H	I	1	2	1	1	1	48	132
Gm11.VB.18	Gm11	3120000	3330000	210000	21	W	W	W	W	W	W	I	5	10	5	5	9	11	352
Gm11.VB.19	Gm11	3330000	3440000	110000	7	W	B	W	W	B	B	I	7	249	8	7	223	218	108
Gm11.VB.20	Gm11	3440000	3590000	150000	22	W	W	W	W	W	W	I	3	15	2	3	10	7	203
Gm11.VB.21	Gm11	3590000	3780000	190000	19	W	B	W	W	B	B	I	28	394	29	30	372	370	181
Gm11.VB.22	Gm11	3780000	3790000	10000	1	W	B	W	W	W	W	W	0	5	0	0	4	4	0
Gm11.VB.23	Gm11	3790000	3900000	110000	12	W	W	W	W	W	W	W	0	14	0	1	11	10	31
Gm11.VB.24	Gm11	3900000	4150000	250000	30	W	B	W	W	B	W	I	12	202	10	11	173	11	366
Gm11.VB.25	Gm11	4150000	4550000	400000	48	W	B	W	W	B	H	I	15	675	15	17	628	394	408
Gm11.VB.26	Gm11	4550000	4680000	130000	16	W	B	W	B	B	H	I	3	296	3	288	279	210	225
Gm11.VB.27	Gm11	4680000	4800000	120000	14	W	B	W	B	B	W	I	21	435	17	418	396	30	107
Gm11.VB.28	Gm11	4800000	5290000	490000	65	W	B	W	B	B	H	I	20	1327	19	1310	1212	1052	1271
Gm11.VB.29	Gm11	5290000	5310000	20000	3	W	W	W	W	W	H	I	0	0	0	0	0	18	79
Gm11.VB.30	Gm11	5310000	5410000	100000	11	W	W	W	W	W	W	I	3	4	1	3	4	1	236
Gm11.VB.31	Gm11	5410000	5460000	50000	7	W	B	W	B	B	W	I	1	203	1	196	197	4	106
Gm11.VB.32	Gm11	5460000	5650000	190000	19	W	W	W	W	W	W	I	25	23	27	27	20	19	345
Gm11.VB.33	Gm11	5650000	5810000	160000	16	W	W	W	W	W	H	I	3	3	3	3	3	77	330
Gm11.VB.34	Gm11	5810000	5840000	30000	3	W	B	W	B	W	B	I	0	130	0	127	0	145	98
Gm11.VB.35	Gm11	5840000	6030000	190000	25	W	B	W	B	W	W	I	1	508	1	504	0	7	482
Gm11.VB.36	Gm11	6030000	6400000	370000	43	W	B	W	B	W	H	I	30	1204	31	1185	24	946	753
Gm11.VB.37	Gm11	6400000	6540000	140000	19	W	W	W	W	W	H	I	5	18	5	18	5	65	314
Gm11.VB.38	Gm11	6540000	6620000	80000	7	W	B	W	B	W	H	I	5	78	4	77	3	96	164
Gm11.VB.39	Gm11	6620000	6980000	360000	38	W	B	W	B	W	W	I	32	891	27	888	29	31	855
Gm11.VB.40	Gm11	6980000	7150000	170000	20	W	W	W	W	W	W	I	0	7	12	6	11	15	304
Gm11.VB.41	Gm11	7150000	7240000	90000	12	W	W	W	W	W	W	I	0	1	3	1	3	31	172
Gm11.VB.42	Gm11	7240000	7460000	220000	25	W	B	B	B	B	B	I	6	790	797	794	729	741	480
Gm11.VB.43	Gm11	7460000	7580000	120000	16	W	B	W	B	W	W	I	13	543	13	539	7	5	350
Gm11.VB.44	Gm11	7580000	7600000	20000	3	W	B	W	B	W	H	I	0	68	0	57	0	19	32
Gm11.VB.45	Gm11	7600000	7850000	250000	28	W	W	W	W	W	H	I	7	12	7	8	6	157	415
Gm11.VB.46	Gm11	7850000	8310000	460000	50	W	B	W	B	W	H	I	32	1696	32	1682	23	1329	739
Gm11.VB.47	Gm11	8310000	8800000	490000	58	W	B	W	B	W	W	I	19	1657	20	1647	16	19	1039
Gm11.VB.48	Gm11	8800000	10510000	1710000	196	W	W	W	W	W	W	I	111	166	107	149	80	110	3098
Gm11.VB.49	Gm11	10510000	10600000	90000	10	W	W	S	W	S	W	I	7	6	104	8	96	5	209
Gm11.VB.50	Gm11	10600000	10780000	180000	18	W	W	W	W	W	W	I	7	8	20	8	19	7	290
Gm11.VB.51	Gm11	10780000	11010000	230000	23	W	W	S	W	S	W	I	6	11	828	8	758	10	737
Gm11.VB.52	Gm11	11010000	11030000	20000	2	W	W	W	W	W	W	I	1	4	10	3	6	2	79

Gm11.VB.53	Gm11	11030000	14970000	3940000	114	W	W	W	W	W	W	I	222	256	259	227	200	206	5272
Gm11.VB.54	Gm11	14970000	15790000	820000	54	W	W	W	W	W	H	I	40	47	47	48	37	578	2605
Gm11.VB.55	Gm11	15790000	15890000	100000	4	W	W	W	W	W	W	I	27	33	29	34	14	42	258
Gm11.VB.56	Gm11	15890000	16060000	170000	13	W	W	W	W	W	W	I	13	13	12	16	11	69	276
Gm11.VB.57	Gm11	16060000	16160000	100000	7	W	B	W	B	W	H	I	9	274	12	272	35	174	172
Gm11.VB.58	Gm11	16160000	16410000	250000	13	W	W	W	W	W	H	I	34	35	34	34	29	180	416
Gm11.VB.59	Gm11	16410000	17050000	640000	42	W	W	W	W	W	W	I	57	79	83	82	49	59	1905
Gm11.VB.60	Gm11	17050000	17110000	60000	2	W	W	S	S	W	W	I	0	1	45	45	1	0	30
Gm11.VB.61	Gm11	17110000	17230000	120000	7	W	W	W	W	W	W	I	14	23	21	20	18	18	219
Gm11.VB.62	Gm11	17230000	17390000	160000	10	W	B	S	S	B	H	I	3	236	208	205	207	74	314
Gm11.VB.63	Gm11	17390000	17460000	70000	8	W	W	S	S	W	S	I	3	4	137	139	6	131	140
Gm11.VB.64	Gm11	17460000	17480000	20000	2	W	W	W	W	W	H	I	0	1	1	1	1	98	21
Gm11.VB.65	Gm11	17480000	17670000	190000	15	W	W	W	W	W	W	I	5	13	12	11	11	11	703
Gm11.VB.66	Gm11	17670000	17990000	320000	14	W	B	B	B	B	B	I	24	468	470	467	473	478	867
Gm11.VB.67	Gm11	17990000	26660000	8670000	102	W	W	W	W	W	W	I	423	945	538	935	943	758	14069
Gm11.VB.68	Gm11	26660000	26760000	100000	4	W	B	W	B	B	W	I	22	77	19	71	73	23	169
Gm11.VB.69	Gm11	26760000	26820000	60000	3	W	W	W	W	W	W	I	0	5	0	5	5	6	287
Gm11.VB.70	Gm11	26820000	26850000	30000	0	W	W	W	W	W	H	I	4	6	4	6	5	31	35
Gm11.VB.71	Gm11	26850000	30020000	3170000	48	W	W	W	W	W	W	I	254	349	261	341	288	346	4206
Gm11.VB.72	Gm11	30020000	30720000	700000	27	W	W	W	W	W	H	I	53	76	82	79	67	1826	1755
Gm11.VB.73	Gm11	30720000	30860000	140000	5	W	W	W	W	W	W	I	10	15	14	15	15	18	493
Gm11.VB.74	Gm11	30860000	30930000	70000	3	W	W	W	W	W	H	I	0	4	6	4	4	211	204
Gm11.VB.75	Gm11	30930000	32920000	1990000	44	W	W	W	W	W	W	I	63	199	62	191	199	167	3587
Gm11.VB.76	Gm11	32920000	33030000	110000	7	W	W	W	W	W	H	I	6	13	6	13	8	367	282
Gm11.VB.77	Gm11	33030000	33090000	60000	3	W	W	W	W	W	W	I	0	3	0	3	3	2	216
Gm11.VB.78	Gm11	33090000	33250000	160000	15	W	B	W	B	B	W	I	5	189	5	191	177	12	146
Gm11.VB.79	Gm11	33250000	34820000	1570000	34	W	W	W	W	W	W	I	49	166	50	167	156	165	1395
Gm11.VB.80	Gm11	34820000	34870000	50000	2	W	W	W	W	W	W	W	0	17	1	17	16	3	12
Gm11.VB.81	Gm11	34870000	34940000	70000	5	W	B	W	B	D	D	I	1	142	1	140	111	109	58
Gm11.VB.82	Gm11	34940000	35090000	150000	10	W	W	W	W	W	W	I	8	16	7	13	13	15	307
Gm11.VB.83	Gm11	35090000	35140000	50000	5	W	W	W	W	W	W	I	11	19	11	14	10	13	179
Gm11.VB.84	Gm11	35140000	35150000	10000	0	W	B	W	B	D	H	I	0	73	0	67	43	54	9
Gm11.VB.85	Gm11	35150000	35320000	170000	14	W	W	W	W	W	W	I	13	20	12	16	18	16	859
Gm11.VB.86	Gm11	35320000	35410000	90000	6	W	B	W	B	B	W	I	23	691	21	673	627	26	418
Gm11.VB.87	Gm11	35410000	35430000	20000	1	W	B	W	B	D	D	I	4	48	4	48	36	37	34
Gm11.VB.88	Gm11	35430000	35620000	190000	10	W	W	W	W	W	W	I	19	26	18	28	28	24	529
Gm11.VB.89	Gm11	35620000	35730000	110000	9	W	W	W	W	W	H	I	9	17	11	16	14	431	210
Gm11.VB.90	Gm11	35730000	35970000	240000	14	W	W	W	W	W	W	I	5	21	9	19	18	25	563
Gm11.VB.91	Gm11	35970000	36100000	130000	10	W	B	W	B	B	W	I	6	250	6	245	233	29	354
Gm11.VB.92	Gm11	36100000	36130000	30000	2	W	B	W	B	D	H	I	5	57	5	58	35	41	33
Gm11.VB.93	Gm11	36130000	36240000	110000	9	W	W	W	W	W	H	I	0	9	0	9	8	180	307
Gm11.VB.94	Gm11	36240000	36630000	390000	34	W	B	W	B	B	B	I	10	1234	10	1222	1133	1150	892
Gm11.VB.95	Gm11	36630000	36730000	100000	9	W	W	W	W	W	W	I	20	18	15	19	12	13	197
Gm11.VB.96	Gm11	36730000	37330000	600000	54	W	B	W	B	B	B	I	56	2258	63	2197	1975	2086	1697
Gm11.VB.97	Gm11	37330000	37570000	240000	28	W	W	W	W	W	W	I	26	40	27	41	34	29	668
Gm11.VB.98	Gm11	37570000	37790000	220000	20	W	B	W	W	B	H	I	6	431	6	18	361	330	326
Gm11.VB.99	Gm11	37790000	38020000	230000	20	W	B	W	B	D	D	I	12	515	12	524	412	428	145
Gm11.VB.100	Gm11	38020000	38170000	150000	22	W	B	W	B	D	W	I	1	466	2	460	177	18	262
Gm11.VB.101	Gm11	38170000	38220000	50000	7	W	B	W	B	D	H	I	1	182	1	181	65	119	101
Gm11.VB.102	Gm11	38220000	38370000	150000	21	W	B	W	B	W	H	I	2	631	2	627	5	289	309
Gm11.VB.103	Gm11	38370000	38480000	110000	15	W	W	W	G	W	H	I	0	8	0	523	1	172	92
Gm11.VB.104	Gm11	38480000	38490000	10000	2	W	B	W	G	W	H	I	0	7	0	11	0	32	9
Gm11.VB.105	Gm11	38490000	38530000	40000	6	W	B	W	G	B	H	I	0	87	0	168	78	36	101
Gm11.VB.106	Gm11	38530000	38730000	200000	26	W	W	W	G	W	H	I	11	24	9	711	14	521	220
Gm11.VB.107	Gm11	38730000	38900000	170000	21	W	B	W	G	B	H	I	0	299	0	680	284	370	474
Gm11.VB.108	Gm11	38900000	39020000	120000	15	W	B	W	W	B	H	I	7	692	8	10	637	505	290
Gm11.VB.109	Gm11	39020000	39060000	40000	2	W	B	W	G	B	W	I	7	35	8	135	28	6	26
Gm11.VB.110	Gm11	39060000	39170000	110000	11	W	B	W	W	B	B	I	2	106	2	3	98	116	216
Gm11.VB.111	Gm11	39170000	39172790	2790	0	W	W	W	W	W	W	W	0	0	0	0	0	0	1
Gm12.VB.1	Gm12	0	700000	700000	84	W	B	S	S	S	H	I	39	1868	1934	1879	1754	1435	1522
Gm12.VB.2	Gm12	700000	910000	210000	25	W	B	S	S	S	W	I	17	882	950	927	846	21	726
Gm12.VB.3	Gm12	910000	920000	10000	3	W	B	S	S	S	H	I	0	63	7	7	7	24	51
Gm12.VB.4	Gm12	920000	1380000	460000	63	W	B	W	W	W	H	I	18	2069	29	30	28	1295	1051
Gm12.VB.5	Gm12	1380000	1660000	280000	28	W	B	S	S	S	H	I	23	1219	1232	1186	1095	910	637
Gm12.VB.6	Gm12	1660000	2330000	670000	77	W	W	S	S	S	W	I	3	26	2315	2292	2151	7	1107
Gm12.VB.7	Gm12	2330000	2380000	50000	4	W	W	S	S	S	W	I	3	9	211	205	184	4	104
Gm12.VB.8	Gm12	2380000	2410000	30000	2	W	B	S	S	D	B	I	0	72	99	97	73	70	39
Gm12.VB.9	Gm12	2410000	2530000	120000	14	W	W	S	S	S	W	I	1	2	438	433	396	2	221
Gm12.VB.10	Gm12	2530000	2720000	190000	35	W	W	S	S	S	H	I	1	11	588	586	541	250	462
Gm12.VB.11	Gm12	2720000	2950000	230000	27	W	B	W	W	W	B	I	18	1033	26	25	15	872	595
Gm12.VB.12	Gm12	2950000	3000000	50000	4	W	B	S	S	S	B	I	0	330	207	204	175	293	179
Gm12.VB.13	Gm12	3000000	3140000	140000	19	W	W	S	S	S	W	I	4	8	902	879	809	10	326
Gm12.VB.14	Gm12	3140000	3440000	300000	35	W	B	S	S	S	B	I	7	1236	875	988	784	1165	862
Gm12.VB.15	Gm12	3440000	3540000	100000	16	W	W	W	W	W	W	I	1	20	6	20	4	6	264
Gm12.VB.16	Gm12	3540000	3650000	110000	12	W	W	S	W	S	H	I	2	9	465	9	438	320	107
Gm12.VB.17	Gm12	3650000	3680000	30000	3	W	B	S	B	S	B	I	0	39	160	42	147	41	96
Gm12.VB.18	Gm12	3680000	3730000	50000	9	W	W	S	W	D	W	I	0	1	174	1	139	0	181
Gm12.VB.19	Gm12	3730000	3820000	90000	10	W	W	W	W	W	W	I	1	5	4	5	4	4	68

Gm12.VB.20	Gm12	3820000	3850000	30000	3	W	B	W	B	W	W	I	5	80	6	79	4	2	61
Gm12.VB.21	Gm12	3850000	3950000	100000	9	W	W	W	W	W	W	I	0	11	2	11	1	2	228
Gm12.VB.22	Gm12	3950000	4140000	190000	17	W	B	W	B	W	W	I	15	506	17	486	10	12	369
Gm12.VB.23	Gm12	4140000	4270000	130000	18	W	B	B	B	B	W	I	25	202	192	200	179	27	301
Gm12.VB.24	Gm12	4270000	4310000	40000	7	W	B	B	B	B	H	I	1	227	244	236	220	93	81
Gm12.VB.25	Gm12	4310000	4530000	220000	19	W	B	B	B	D	W	I	5	741	773	733	623	24	334
Gm12.VB.26	Gm12	4530000	4610000	80000	9	W	B	B	B	B	H	I	14	199	203	196	170	247	183
Gm12.VB.27	Gm12	4610000	4900000	290000	32	W	B	W	B	W	H	I	9	789	18	771	18	798	656
Gm12.VB.28	Gm12	4900000	5080000	180000	21	W	B	W	B	W	W	I	3	662	9	645	9	8	471
Gm12.VB.29	Gm12	5080000	5160000	80000	11	W	W	W	W	W	W	I	3	9	8	8	10	7	119
Gm12.VB.30	Gm12	5160000	5220000	60000	4	W	W	S	W	S	W	I	1	2	353	1	315	3	145
Gm12.VB.31	Gm12	5220000	5440000	220000	20	W	W	S	W	S	H	I	27	33	1407	29	1278	1056	863
Gm12.VB.32	Gm12	5440000	5460000	20000	0	W	W	S	W	D	W	I	14	16	87	16	67	8	68
Gm12.VB.33	Gm12	5460000	5490000	30000	1	W	B	S	B	S	W	I	4	90	193	90	174	4	104
Gm12.VB.34	Gm12	5490000	5740000	250000	15	W	W	S	W	S	W	I	5	11	987	11	854	12	560
Gm12.VB.35	Gm12	5740000	6020000	280000	20	W	W	W	W	W	W	I	11	15	17	16	13	11	537
Gm12.VB.36	Gm12	6020000	6040000	20000	1	W	B	B	B	B	B	I	4	57	53	57	49	50	54
Gm12.VB.37	Gm12	6040000	6180000	140000	11	W	B	W	B	W	B	I	5	807	6	788	2	763	374
Gm12.VB.38	Gm12	6180000	6500000	320000	28	W	B	S	B	S	B	I	52	1359	2053	1334	1814	1237	1066
Gm12.VB.39	Gm12	6500000	6520000	20000	1	W	W	S	W	S	W	I	0	1	47	1	42	1	15
Gm12.VB.40	Gm12	6520000	6740000	220000	14	W	W	W	W	W	W	I	30	42	44	41	32	33	678
Gm12.VB.41	Gm12	6740000	6830000	90000	4	W	W	W	W	W	W	I	14	20	23	11	18	16	180
Gm12.VB.42	Gm12	6830000	6870000	40000	4	W	W	S	W	S	W	I	0	5	127	5	105	5	103
Gm12.VB.43	Gm12	6870000	7060000	190000	15	W	B	S	B	S	B	I	34	606	1027	591	957	559	638
Gm12.VB.44	Gm12	7060000	7160000	100000	10	W	B	W	B	W	B	I	10	505	15	499	16	498	251
Gm12.VB.45	Gm12	7160000	7280000	120000	7	W	B	S	B	S	B	I	6	539	735	540	636	494	396
Gm12.VB.46	Gm12	7280000	7420000	140000	14	W	W	S	W	S	W	I	3	7	944	8	850	12	512
Gm12.VB.47	Gm12	7420000	7580000	160000	14	W	W	S	W	S	H	I	10	19	904	18	842	352	604
Gm12.VB.48	Gm12	7580000	7600000	20000	3	W	B	S	B	S	B	I	15	96	152	89	129	92	86
Gm12.VB.49	Gm12	7600000	7710000	110000	8	W	W	S	W	S	W	I	7	18	872	19	795	6	472
Gm12.VB.50	Gm12	7710000	7770000	60000	3	W	B	S	B	S	W	I	1	131	304	126	295	3	283
Gm12.VB.51	Gm12	7770000	7880000	110000	3	W	W	S	W	S	W	I	15	23	482	22	421	24	246
Gm12.VB.52	Gm12	7880000	8070000	190000	10	W	B	S	B	S	H	I	38	722	731	700	668	664	364
Gm12.VB.53	Gm12	8070000	8080000	10000	0	W	B	S	B	D	W	W	0	5	119	5	111	4	2
Gm12.VB.54	Gm12	8080000	8390000	310000	16	W	W	S	W	S	W	I	42	66	2019	64	1854	57	928
Gm12.VB.55	Gm12	8390000	8460000	70000	4	W	B	S	B	S	W	I	0	455	445	448	400	2	199
Gm12.VB.56	Gm12	8460000	8500000	40000	2	W	B	W	B	W	W	W	0	190	1	189	1	0	10
Gm12.VB.57	Gm12	8500000	8870000	370000	20	W	W	W	W	W	W	I	8	27	32	28	29	30	1043
Gm12.VB.58	Gm12	8870000	8950000	80000	6	W	B	B	B	B	W	I	41	447	465	444	440	37	501
Gm12.VB.59	Gm12	8950000	9150000	200000	14	W	B	B	B	B	B	I	9	1022	1034	987	953	977	731
Gm12.VB.60	Gm12	9150000	9320000	170000	10	W	W	W	W	W	W	I	6	17	13	23	11	18	657
Gm12.VB.61	Gm12	9320000	9770000	450000	17	W	B	W	B	W	W	I	47	2129	48	2089	36	58	2136
Gm12.VB.62	Gm12	9770000	11470000	1700000	66	W	B	W	B	W	B	I	98	6731	100	6641	74	6522	5626
Gm12.VB.63	Gm12	11470000	12480000	1010000	29	W	W	W	W	W	W	I	68	132	56	124	63	131	3750
Gm12.VB.64	Gm12	12480000	12770000	290000	13	W	B	W	B	W	B	I	24	1154	23	1140	21	1092	1346
Gm12.VB.65	Gm12	12770000	13250000	480000	15	W	W	W	W	W	W	I	37	73	37	78	47	79	1649
Gm12.VB.66	Gm12	13250000	13970000	720000	51	W	B	W	B	W	B	I	27	834	25	835	25	819	2018
Gm12.VB.67	Gm12	13970000	14100000	130000	3	W	W	W	W	W	W	I	2	25	2	24	2	26	376
Gm12.VB.68	Gm12	14100000	14130000	30000	0	W	W	W	W	W	W	I	6	14	7	12	4	10	74
Gm12.VB.69	Gm12	14130000	14850000	720000	33	W	W	W	W	W	W	I	2077	2016	2022	2000	1981	2015	1992
Gm12.VB.70	Gm12	14850000	14860000	10000	1	W	B	B	W	B	H	I	9	4	4	8	4	6	27
Gm12.VB.71	Gm12	14860000	15050000	190000	5	W	W	W	W	W	W	I	29	27	31	28	29	26	752
Gm12.VB.72	Gm12	15050000	17300000	2250000	53	W	W	W	W	W	W	I	6035	5944	5967	5898	5832	5938	6491
Gm12.VB.73	Gm12	17300000	17360000	60000	0	W	W	W	W	W	W	W	3	6	4	3	3	6	19
Gm12.VB.74	Gm12	17360000	17420000	60000	1	W	W	W	W	W	W	W	11	10	10	11	11	11	16
Gm12.VB.75	Gm12	17420000	17470000	50000	0	W	W	W	W	W	W	W	13	12	11	12	10	12	12
Gm12.VB.76	Gm12	17470000	17950000	480000	11	W	W	W	W	W	W	I	1949	1936	1914	1893	1861	1866	1609
Gm12.VB.77	Gm12	17950000	18260000	310000	4	W	B	B	B	B	W	I	10	1148	1152	1101	1180	10	206
Gm12.VB.78	Gm12	18260000	21810000	3550000	27	W	W	W	W	W	W	I	352	304	325	331	343	376	1814
Gm12.VB.79	Gm12	21810000	21830000	20000	0	W	W	S	G	W	W	I	1	4	9	11	5	0	11
Gm12.VB.80	Gm12	21830000	21850000	20000	0	W	W	W	W	W	W	W	0	0	8	1	7	4	5
Gm12.VB.81	Gm12	21850000	21980000	130000	4	W	W	W	W	W	W	I	191	191	193	191	171	163	476
Gm12.VB.82	Gm12	21980000	22110000	130000	0	W	W	W	W	W	W	I	18	20	20	14	13	17	76
Gm12.VB.83	Gm12	22110000	22350000	240000	5	W	W	W	W	W	W	I	664	642	651	641	657	662	963
Gm12.VB.84	Gm12	22350000	22360000	10000	0	W	W	W	W	W	W	I	5	4	5	5	5	4	84
Gm12.VB.85	Gm12	22360000	22450000	90000	1	W	W	W	W	W	W	I	16	9	10	11	11	13	265
Gm12.VB.86	Gm12	22450000	24260000	1810000	21	W	W	W	W	W	W	I	252	240	238	230	228	239	1661
Gm12.VB.87	Gm12	24260000	24560000	300000	10	W	W	W	W	W	W	I	1271	1260	1285	1220	1120	1156	1160
Gm12.VB.88	Gm12	24560000	24570000	10000	0	W	W	W	W	W	W	I	7	7	7	7	3	7	15
Gm12.VB.89	Gm12	24570000	26090000	1520000	29	W	W	W	W	W	W	I	92	100	94	94	93	91	3985
Gm12.VB.90	Gm12	26090000	26420000	330000	0	W	B	S	G	D	H	I	287	269	282	263	265	243	407
Gm12.VB.91	Gm12	26420000	33070000	6650000	120	W	W	W	W	W	W	I	453	455	436	404	404	431	7587
Gm12.VB.92	Gm12	33070000	33200000	130000	7	W	B	W	W	W	W	I	22	458	28	18	26	26	390
Gm12.VB.93	Gm12	33200000	33650000	450000	19	W	W	W	W	W	W	I	10	26	12	10	10	19	1984
Gm12.VB.94	Gm12	33650000	33670000	20000	3	W	B	W	W	W	B	I	0	37	0	0	0	34	44
Gm12.VB.95	Gm12	33670000	33830000	160000	5	W	B	W	W	W	W	I	1	91	1	1	1	7	608
Gm12.VB.96	Gm12	33830000	33840000	10000	1	W	B	W	W	W	B	I	0	9	0	0	0	9	35
Gm12.VB.97	Gm12	33840000	33880000	40000	1	W	W	W	W	W	H	I	0	3	0	0	0	110	51

Gm12.VB.98	Gm12	33880000	34090000	210000	15	W	W	W	W	W	W	I	12	29	12	12	13	31	681
Gm12.VB.99	Gm12	34090000	34220000	130000	12	W	B	W	W	W	W	I	11	193	13	13	10	17	593
Gm12.VB.100	Gm12	34220000	34280000	60000	5	W	W	W	W	W	W	I	1	9	1	1	1	5	49
Gm12.VB.101	Gm12	34280000	34370000	90000	6	W	W	W	W	W	H	I	5	12	5	6	3	68	272
Gm12.VB.102	Gm12	34370000	34480000	110000	10	W	B	W	W	W	B	I	1	762	1	1	1	761	388
Gm12.VB.103	Gm12	34480000	35280000	800000	68	W	B	W	W	W	W	I	86	2257	88	90	60	67	2492
Gm12.VB.104	Gm12	35280000	35330000	50000	6	W	B	W	W	W	B	I	0	105	0	0	0	103	157
Gm12.VB.105	Gm12	35330000	35490000	160000	13	W	B	W	W	W	W	I	3	451	2	3	1	9	170
Gm12.VB.106	Gm12	35490000	35710000	220000	19	W	B	W	W	W	H	I	16	623	21	20	14	410	510
Gm12.VB.107	Gm12	35710000	35760000	50000	6	W	B	W	W	B	H	I	1	71	1	1	64	45	41
Gm12.VB.108	Gm12	35760000	35980000	220000	21	W	W	W	W	W	H	I	28	29	29	28	29	163	623
Gm12.VB.109	Gm12	35980000	36280000	300000	36	W	W	W	W	W	W	I	29	31	25	27	24	34	817
Gm12.VB.110	Gm12	36280000	36350000	70000	7	W	W	W	W	W	W	I	7	8	7	6	1	14	220
Gm12.VB.111	Gm12	36350000	36440000	90000	9	W	B	W	W	B	B	I	15	377	15	14	357	356	217
Gm12.VB.112	Gm12	36440000	36950000	510000	55	W	W	W	W	W	W	I	24	42	28	27	30	40	1483
Gm12.VB.113	Gm12	36950000	37840000	890000	71	W	B	W	W	B	W	I	126	4490	135	98	4050	77	2522
Gm12.VB.114	Gm12	37840000	37950000	110000	11	W	W	W	W	W	W	I	6	9	4	6	4	4	390
Gm12.VB.115	Gm12	37950000	38210000	260000	22	W	B	W	W	W	W	I	14	1029	14	11	64	12	644
Gm12.VB.116	Gm12	38210000	38570000	360000	32	W	W	W	W	W	W	I	71	77	77	70	44	52	895
Gm12.VB.117	Gm12	38570000	38920000	350000	28	W	B	W	W	W	W	I	38	779	43	38	24	26	922
Gm12.VB.118	Gm12	38920000	40110000	1190000	111	W	W	W	W	W	W	I	65	96	71	60	50	57	3181
Gm12.VB.119	Gm12	40110000	40113140	3140	0	W	W	W	W	W	W	I	0	0	0	0	0	0	10
Gm13.VB.1	Gm13	0	230000	230000	23	W	W	S	W	S	H	I	14	16	448	14	405	112	491
Gm13.VB.2	Gm13	230000	400000	170000	15	W	B	S	B	S	H	I	36	522	520	511	451	348	315
Gm13.VB.3	Gm13	400000	560000	160000	13	W	W	W	W	W	W	I	10	18	22	19	17	15	289
Gm13.VB.4	Gm13	560000	680000	120000	9	W	B	W	B	W	W	I	4	192	15	184	14	11	112
Gm13.VB.5	Gm13	680000	720000	40000	3	W	B	B	B	B	B	I	0	221	225	221	191	189	140
Gm13.VB.6	Gm13	720000	800000	80000	8	W	B	W	B	W	H	I	11	193	11	198	12	225	213
Gm13.VB.7	Gm13	800000	3610000	2810000	151	W	W	W	W	W	W	I	201	235	203	233	147	167	8479
Gm13.VB.8	Gm13	3610000	3750000	140000	6	W	W	W	W	W	H	I	2	8	2	9	1	656	125
Gm13.VB.9	Gm13	3750000	4180000	430000	14	W	W	W	W	W	W	I	60	70	61	54	27	58	754
Gm13.VB.10	Gm13	4180000	4420000	240000	6	W	W	W	W	W	H	I	8	22	8	19	7	817	608
Gm13.VB.11	Gm13	4420000	4550000	130000	6	W	W	W	W	W	W	I	16	20	17	22	5	19	341
Gm13.VB.12	Gm13	4550000	4750000	200000	9	W	W	W	W	W	H	I	8	13	7	15	6	641	440
Gm13.VB.13	Gm13	4750000	4790000	40000	3	W	B	W	B	W	H	I	4	80	3	79	3	20	89
Gm13.VB.14	Gm13	4790000	5150000	360000	27	W	B	W	W	W	H	I	28	760	27	30	20	863	665
Gm13.VB.15	Gm13	5150000	5160000	10000	1	W	B	W	W	W	W	I	1	22	1	1	1	1	25
Gm13.VB.16	Gm13	5160000	5330000	170000	11	W	W	W	W	W	W	I	20	29	19	20	20	24	539
Gm13.VB.17	Gm13	5330000	5460000	130000	9	W	B	W	W	W	W	I	27	95	29	29	24	25	243
Gm13.VB.18	Gm13	5460000	5560000	100000	5	W	W	W	W	W	W	I	4	11	4	4	4	3	449
Gm13.VB.19	Gm13	5560000	5920000	360000	18	W	B	W	W	W	W	I	12	1480	16	15	11	14	1362
Gm13.VB.20	Gm13	5920000	6070000	150000	13	W	W	W	W	W	W	I	9	18	9	9	5	6	372
Gm13.VB.21	Gm13	6070000	7140000	1070000	74	W	B	W	W	W	W	I	90	4647	92	81	66	64	3646
Gm13.VB.22	Gm13	7140000	7450000	310000	23	W	B	W	W	W	H	I	48	1511	39	46	41	1924	775
Gm13.VB.23	Gm13	7450000	7660000	210000	7	W	W	W	W	W	H	I	7	22	14	6	7	757	620
Gm13.VB.24	Gm13	7660000	7980000	320000	23	W	B	W	W	W	H	I	15	1800	15	15	14	2112	1098
Gm13.VB.25	Gm13	7980000	8160000	180000	11	W	B	W	W	D	H	I	3	364	3	2	278	756	540
Gm13.VB.26	Gm13	8160000	10800000	2640000	67	W	W	W	W	W	H	I	131	265	135	259	228	10220	6303
Gm13.VB.27	Gm13	10800000	14780000	3980000	118	W	B	W	B	W	H	I	136	12745	117	12574	103	12492	10038
Gm13.VB.28	Gm13	14780000	15550000	770000	24	W	W	W	W	W	W	W	7	7	2	16	3	6	28
Gm13.VB.29	Gm13	15550000	16000000	450000	8	W	B	W	G	W	H	I	87	406	92	356	94	335	542
Gm13.VB.30	Gm13	16000000	16010000	10000	1	W	W	W	G	W	H	I	2	3	5	7	1	8	12
Gm13.VB.31	Gm13	16010000	16060000	50000	2	W	W	W	W	W	W	I	1	15	1	14	1	15	62
Gm13.VB.32	Gm13	16060000	16130000	70000	0	W	W	W	W	W	W	I	0	16	1	16	0	16	79
Gm13.VB.33	Gm13	16130000	16170000	40000	0	W	W	W	G	W	W	I	1	17	1	18	1	9	37
Gm13.VB.34	Gm13	16170000	16180000	10000	0	W	W	W	W	W	W	I	0	2	0	2	0	3	18
Gm13.VB.35	Gm13	16180000	16300000	120000	3	W	W	W	W	W	W	I	4	36	2	37	2	33	101
Gm13.VB.36	Gm13	16300000	20330000	4030000	29	W	B	W	B	W	H	I	303	5743	290	5628	294	5767	2354
Gm13.VB.37	Gm13	20330000	20600000	270000	23	W	W	W	W	W	W	I	9	17	13	18	10	14	414
Gm13.VB.38	Gm13	20600000	20610000	10000	0	W	B	W	B	W	W	I	0	25	2	26	0	0	10
Gm13.VB.39	Gm13	20610000	20710000	100000	12	W	W	W	W	W	W	I	5	8	8	7	6	5	200
Gm13.VB.40	Gm13	20710000	20720000	10000	2	W	W	S	W	S	W	I	0	0	10	0	8	0	20
Gm13.VB.41	Gm13	20720000	20770000	50000	7	W	W	S	W	S	H	I	0	2	174	2	164	82	93
Gm13.VB.42	Gm13	20770000	20780000	10000	1	W	W	S	W	S	W	I	0	1	5	1	5	3	11
Gm13.VB.43	Gm13	20780000	20840000	60000	9	W	W	W	W	W	W	I	0	2	0	2	0	0	102
Gm13.VB.44	Gm13	20840000	21000000	160000	16	W	B	W	B	W	W	I	26	414	39	406	36	30	271
Gm13.VB.45	Gm13	21000000	21150000	150000	15	W	W	W	W	W	W	I	23	21	39	20	33	29	263
Gm13.VB.46	Gm13	21150000	21230000	80000	3	W	W	S	W	S	H	I	3	4	250	3	237	146	183
Gm13.VB.47	Gm13	21230000	21500000	270000	30	W	W	W	W	W	W	I	3	3	9	3	9	15	384
Gm13.VB.48	Gm13	21500000	22780000	1280000	117	W	W	W	W	W	H	I	58	65	58	59	36	4021	2573
Gm13.VB.49	Gm13	22780000	22940000	160000	19	W	W	W	W	W	W	I	6	6	6	6	6	12	441
Gm13.VB.50	Gm13	22940000	23150000	210000	20	W	W	W	W	W	H	I	28	28	28	25	31	850	601
Gm13.VB.51	Gm13	23150000	23260000	110000	9	W	W	W	W	W	W	I	12	12	12	8	13	19	196
Gm13.VB.52	Gm13	23260000	23280000	20000	0	W	W	W	W	W	H	I	22	20	20	19	5	36	60
Gm13.VB.53	Gm13	23280000	23650000	370000	34	W	W	S	S	S	H	I	29	29	1294	1271	1221	724	1030
Gm13.VB.54	Gm13	23650000	23670000	20000	1	W	W	S	S	S	W	I	0	0	13	13	12	7	10
Gm13.VB.55	Gm13	23670000	24210000	540000	45	W	W	W	W	W	W	I	55	75	85	82	78	74	1493
Gm13.VB.56	Gm13	24210000	24410000	200000	15	W	B	B	B	B	B	I	13	374	383	368	327	342	295

Gm13.VB.57	Gm13	24410000	25160000	750000	51	W	W	W	W	W	W	I	63	108	121	107	112	109	2042
Gm13.VB.58	Gm13	25160000	25550000	390000	25	W	B	B	B	B	B	I	19	956	968	945	892	910	655
Gm13.VB.59	Gm13	25550000	26030000	480000	39	W	B	W	W	W	B	I	48	1382	56	53	45	1235	1050
Gm13.VB.60	Gm13	26030000	26140000	110000	6	W	B	B	B	B	B	I	0	458	442	440	429	432	159
Gm13.VB.61	Gm13	26140000	26260000	120000	10	W	W	W	W	W	W	I	1	6	9	9	9	6	204
Gm13.VB.62	Gm13	26260000	26270000	10000	0	W	W	S	S	S	W	I	7	11	43	39	34	7	10
Gm13.VB.63	Gm13	26270000	26380000	110000	4	W	B	B	B	B	H	I	11	413	401	399	362	286	206
Gm13.VB.64	Gm13	26380000	26520000	140000	14	W	W	S	S	S	W	I	20	28	506	501	471	22	350
Gm13.VB.65	Gm13	26520000	32030000	5510000	500	W	B	S	S	S	H	I	436	27326	26515	25418	23533	21398	15180
Gm13.VB.66	Gm13	32030000	32130000	100000	9	W	W	S	S	S	S	I	2	2	277	270	248	255	232
Gm13.VB.67	Gm13	32130000	32480000	350000	37	W	B	S	S	S	S	I	12	865	1047	1025	931	933	695
Gm13.VB.68	Gm13	32480000	32610000	130000	15	W	W	S	S	S	H	I	12	30	458	459	405	438	272
Gm13.VB.69	Gm13	32610000	32960000	350000	38	W	B	S	S	S	H	I	14	1190	903	859	825	454	403
Gm13.VB.70	Gm13	32960000	33470000	510000	62	W	W	S	S	S	S	I	12	37	2209	2139	1929	2057	1376
Gm13.VB.71	Gm13	33470000	33670000	200000	18	W	B	S	S	S	H	I	2	474	473	465	396	279	245
Gm13.VB.72	Gm13	33670000	33740000	70000	6	W	W	S	S	S	H	I	10	15	249	243	225	47	82
Gm13.VB.73	Gm13	33740000	33760000	20000	2	W	W	S	S	S	W	I	0	0	12	12	12	3	40
Gm13.VB.74	Gm13	33760000	34200000	440000	49	W	W	W	W	W	W	I	18	54	43	45	40	30	987
Gm13.VB.75	Gm13	34200000	34240000	40000	6	W	B	W	W	W	W	I	2	102	2	1	1	0	38
Gm13.VB.76	Gm13	34240000	34430000	190000	24	W	W	W	W	W	W	I	3	9	8	8	8	13	315
Gm13.VB.77	Gm13	34430000	34480000	50000	5	W	B	B	B	B	B	I	0	168	177	161	154	155	154
Gm13.VB.78	Gm13	34480000	34490000	10000	1	W	B	B	B	D	W	W	0	7	6	6	5	4	4
Gm13.VB.79	Gm13	34490000	34610000	120000	16	W	W	W	W	W	W	I	9	9	14	14	8	4	86
Gm13.VB.80	Gm13	34610000	35200000	590000	67	W	B	B	B	B	B	I	32	2126	2139	2098	1944	1919	1401
Gm13.VB.81	Gm13	35200000	35270000	70000	6	W	B	W	W	B	B	I	0	112	1	1	104	107	95
Gm13.VB.82	Gm13	35270000	35640000	370000	42	W	W	W	W	W	W	I	29	48	65	66	67	48	882
Gm13.VB.83	Gm13	35640000	35760000	120000	10	W	B	W	W	W	W	I	22	222	25	23	18	28	245
Gm13.VB.84	Gm13	35760000	35810000	50000	2	W	B	W	W	W	H	I	20	227	22	20	17	46	106
Gm13.VB.85	Gm13	35810000	36770000	960000	100	W	B	S	S	S	H	I	51	3797	3654	3529	3271	2037	1806
Gm13.VB.86	Gm13	36770000	36800000	30000	3	W	W	S	S	S	W	I	2	4	26	28	25	3	75
Gm13.VB.87	Gm13	36800000	36870000	70000	7	W	W	W	W	W	W	I	0	0	0	0	0	0	162
Gm13.VB.88	Gm13	36870000	36890000	20000	1	W	B	W	W	W	B	I	0	59	1	0	0	53	44
Gm13.VB.89	Gm13	36890000	37170000	280000	27	W	W	W	W	W	W	I	30	41	81	80	69	47	702
Gm13.VB.90	Gm13	37170000	37210000	40000	5	W	B	B	B	B	W	I	0	131	132	123	115	0	61
Gm13.VB.91	Gm13	37210000	37280000	70000	4	W	B	W	W	W	W	I	16	291	19	20	12	10	244
Gm13.VB.92	Gm13	37280000	37960000	680000	66	W	W	W	W	W	W	I	38	49	53	57	48	43	384
Gm13.VB.93	Gm13	37960000	38170000	210000	24	W	B	B	B	B	W	I	20	853	895	818	768	22	486
Gm13.VB.94	Gm13	38170000	38340000	170000	20	W	W	W	W	W	W	W	16	76	41	38	32	22	36
Gm13.VB.95	Gm13	38340000	38410000	70000	9	W	B	W	W	W	W	B	7	179	20	20	16	6	148
Gm13.VB.96	Gm13	38410000	38590000	180000	23	W	B	S	S	S	W	I	2	439	514	505	488	6	252
Gm13.VB.97	Gm13	38590000	38690000	100000	12	W	W	S	S	S	W	I	0	12	294	287	282	3	181
Gm13.VB.98	Gm13	38690000	38990000	300000	37	W	B	S	S	S	W	I	35	559	1095	1090	968	25	589
Gm13.VB.99	Gm13	38990000	39100000	110000	17	W	B	S	S	D	H	I	2	363	434	427	349	290	297
Gm13.VB.100	Gm13	39100000	39390000	290000	30	W	W	W	W	W	W	I	16	26	38	41	35	17	894
Gm13.VB.101	Gm13	39390000	39690000	300000	32	W	B	W	W	W	W	I	14	1593	51	51	44	12	961
Gm13.VB.102	Gm13	39690000	40110000	420000	46	W	B	S	S	S	W	I	39	1610	1035	861	876	50	708
Gm13.VB.103	Gm13	40110000	40440000	330000	28	W	W	W	W	W	W	I	10	13	13	13	9	17	461
Gm13.VB.104	Gm13	40440000	40760000	320000	27	W	W	W	W	W	H	I	10	11	12	13	8	816	412
Gm13.VB.105	Gm13	40760000	41280000	520000	54	W	W	S	S	S	S	I	46	48	846	813	700	697	627
Gm13.VB.106	Gm13	41280000	41320000	40000	3	W	W	S	S	W	S	I	13	17	59	53	5	40	26
Gm13.VB.107	Gm13	41320000	41390000	70000	9	W	W	S	W	W	S	I	9	6	46	6	5	48	246
Gm13.VB.108	Gm13	41390000	42530000	1140000	142	W	W	W	W	W	W	I	76	70	108	62	55	86	2804
Gm13.VB.109	Gm13	42530000	42940000	410000	45	W	W	S	W	W	W	I	16	18	1112	17	14	10	1116
Gm13.VB.110	Gm13	42940000	44400000	1460000	165	W	W	W	W	W	W	I	49	53	72	50	32	37	1550
Gm13.VB.111	Gm13	44400000	44408971	8971	1	W	W	W	W	W	W	I	0	0	0	0	0	0	4
Gm14.VB.1	Gm14	0	290000	290000	25	W	B	S	S	S	W	I	7	635	429	422	411	6	256
Gm14.VB.2	Gm14	290000	4910000	4620000	515	W	B	S	S	B	H	I	2822	15054	18792	18226	14418	14797	10044
Gm14.VB.3	Gm14	4910000	5060000	150000	16	W	W	S	S	W	S	I	4	4	600	575	4	565	313
Gm14.VB.4	Gm14	5060000	5920000	860000	81	W	W	S	S	W	W	I	53	79	3613	3542	64	65	2007
Gm14.VB.5	Gm14	5920000	6050000	130000	14	W	W	S	S	W	H	I	23	21	671	636	15	302	299
Gm14.VB.6	Gm14	6050000	6420000	370000	33	W	W	S	S	W	W	I	15	19	1993	1936	12	22	1229
Gm14.VB.7	Gm14	6420000	6610000	190000	14	W	W	S	S	W	H	I	16	20	960	920	12	283	497
Gm14.VB.8	Gm14	6610000	7060000	450000	36	W	W	S	S	W	W	I	34	33	1281	1260	36	34	1271
Gm14.VB.9	Gm14	7060000	7120000	60000	3	W	B	B	B	B	H	I	8	276	286	284	264	186	155
Gm14.VB.10	Gm14	7120000	7210000	90000	8	W	W	S	S	D	W	I	4	40	147	139	41	5	156
Gm14.VB.11	Gm14	7210000	7440000	230000	13	W	W	S	S	W	W	I	19	26	1132	1116	21	16	686
Gm14.VB.12	Gm14	7440000	7520000	80000	5	W	W	S	S	W	H	I	31	28	970	902	13	715	353
Gm14.VB.13	Gm14	7520000	7630000	110000	6	W	W	W	W	W	W	I	15	15	20	20	15	24	450
Gm14.VB.14	Gm14	7630000	7880000	250000	19	W	W	W	W	W	H	I	28	28	36	33	23	131	605
Gm14.VB.15	Gm14	7880000	8030000	150000	13	W	W	S	S	W	H	I	2	2	782	765	2	480	673
Gm14.VB.16	Gm14	8030000	8280000	250000	10	W	W	W	W	W	H	I	8	9	11	11	4	218	892
Gm14.VB.17	Gm14	8280000	8290000	10000	0	W	W	S	S	W	H	I	0	0	50	42	0	10	7
Gm14.VB.18	Gm14	8290000	8300000	10000	0	W	W	S	S	W	W	I	0	0	46	46	0	3	7
Gm14.VB.19	Gm14	8300000	8660000	360000	19	W	W	W	W	W	W	I	12	12	38	36	11	25	909
Gm14.VB.20	Gm14	8660000	9280000	620000	44	W	W	S	S	W	W	I	31	37	2081	2047	37	52	1657
Gm14.VB.21	Gm14	9280000	9290000	10000	0	W	W	W	W	W	W	I	7	7	7	7	7	10	14
Gm14.VB.22	Gm14	9290000	9620000	330000	13	W	W	W	W	W	H	I	24	22	27	19	14	236	1533
Gm14.VB.23	Gm14	9620000	9820000	200000	6	W	W	W	W	W	W	I	16	17	19	16	13	23	488

Gm14.VB.24	Gm14	9820000	10210000	390000	16	W	W	W	W	W	H	I	16	12	27	27	10	224	622
Gm14.VB.25	Gm14	10210000	10580000	370000	18	W	W	W	W	W	W	I	14	15	19	17	13	62	1299
Gm14.VB.26	Gm14	10580000	10960000	380000	8	W	W	W	W	W	H	I	22	21	25	25	10	270	1189
Gm14.VB.27	Gm14	10960000	12810000	1850000	41	W	W	W	W	W	W	I	85	92	111	99	110	124	2234
Gm14.VB.28	Gm14	12810000	13110000	300000	6	W	W	W	W	W	H	I	13	12	23	20	9	223	578
Gm14.VB.29	Gm14	13110000	19230000	6120000	97	W	W	W	W	W	W	I	364	501	487	441	435	452	13086
Gm14.VB.30	Gm14	19230000	19240000	10000	0	W	W	W	W	D	W	I	4	6	2	5	8	5	46
Gm14.VB.31	Gm14	19240000	19610000	370000	9	W	B	W	W	B	W	I	23	182	29	22	173	25	846
Gm14.VB.32	Gm14	19610000	42540000	22930000	289	W	W	W	W	W	W	I	1167	1887	1807	1646	1821	1619	64008
Gm14.VB.33	Gm14	42540000	42630000	90000	3	W	B	B	B	B	W	I	6	209	223	217	216	12	295
Gm14.VB.34	Gm14	42630000	42680000	50000	0	W	B	B	B	B	H	I	1	262	264	264	264	88	694
Gm14.VB.35	Gm14	42680000	43030000	350000	7	W	B	B	B	B	W	I	28	1360	1396	1353	1327	53	880
Gm14.VB.36	Gm14	43030000	43350000	320000	8	W	B	B	B	B	H	I	23	1767	1801	1749	1759	1028	1405
Gm14.VB.37	Gm14	43350000	43880000	530000	17	W	W	W	W	W	H	I	51	61	63	58	57	983	2139
Gm14.VB.38	Gm14	43880000	45020000	1140000	44	W	W	W	W	W	W	I	79	88	105	86	82	97	3361
Gm14.VB.39	Gm14	45020000	45030000	10000	0	W	W	S	S	W	W	I	0	0	26	26	0	0	26
Gm14.VB.40	Gm14	45030000	45150000	120000	10	W	W	W	W	W	W	I	3	5	7	7	4	3	539
Gm14.VB.41	Gm14	45150000	45510000	360000	20	W	B	W	W	B	W	I	3	2184	16	18	2071	4	1085
Gm14.VB.42	Gm14	45510000	45900000	390000	17	W	W	W	W	W	W	I	15	23	35	37	13	13	1441
Gm14.VB.43	Gm14	45900000	45910000	10000	0	W	W	S	S	W	W	W	0	0	70	60	1	1	1
Gm14.VB.44	Gm14	45910000	46160000	250000	21	W	W	W	W	W	W	I	12	11	16	16	7	15	1060
Gm14.VB.45	Gm14	46160000	46170000	10000	1	W	W	S	W	W	W	I	1	3	7	6	1	1	49
Gm14.VB.46	Gm14	46170000	46950000	780000	67	W	W	S	S	W	W	I	37	40	3663	3491	29	34	2567
Gm14.VB.47	Gm14	46950000	47180000	230000	20	W	W	S	S	W	H	I	22	20	1184	1146	19	555	444
Gm14.VB.48	Gm14	47180000	47330000	150000	14	W	W	W	W	W	H	I	27	34	35	33	35	265	293
Gm14.VB.49	Gm14	47330000	47560000	230000	20	W	W	S	S	W	H	I	30	358	1369	1314	345	746	836
Gm14.VB.50	Gm14	47560000	47690000	130000	6	W	B	S	S	D	H	I	9	817	845	789	742	717	438
Gm14.VB.51	Gm14	47690000	47760000	70000	5	W	B	S	S	D	W	I	15	181	473	465	191	16	202
Gm14.VB.52	Gm14	47760000	47820000	60000	3	W	W	S	S	W	W	I	6	9	303	295	8	6	172
Gm14.VB.53	Gm14	47820000	47880000	60000	3	W	W	S	S	W	H	I	1	3	635	597	3	689	636
Gm14.VB.54	Gm14	47880000	48080000	200000	16	W	B	B	B	B	H	I	4	597	604	595	558	495	381
Gm14.VB.55	Gm14	48080000	48210000	130000	14	W	W	S	S	W	S	I	1	9	844	817	9	781	473
Gm14.VB.56	Gm14	48210000	48450000	240000	23	W	B	S	S	B	S	I	14	635	1193	1160	571	1105	565
Gm14.VB.57	Gm14	48450000	48550000	100000	11	W	W	S	S	W	H	I	3	3	766	760	3	699	269
Gm14.VB.58	Gm14	48550000	48650000	100000	9	W	W	W	W	W	H	I	11	11	13	12	5	443	211
Gm14.VB.59	Gm14	48650000	48730000	80000	12	W	W	S	S	W	H	I	4	3	293	296	7	226	171
Gm14.VB.60	Gm14	48730000	49090000	360000	24	W	W	W	W	W	W	I	55	75	69	61	71	68	971
Gm14.VB.61	Gm14	49090000	49130000	40000	4	W	B	W	W	W	W	I	1	69	2	2	1	3	39
Gm14.VB.62	Gm14	49130000	49150000	20000	3	W	B	B	B	B	W	I	1	40	43	43	40	2	50
Gm14.VB.63	Gm14	49150000	49200000	50000	5	W	W	S	S	W	W	I	1	1	56	48	1	4	164
Gm14.VB.64	Gm14	49200000	49710000	510000	41	W	W	S	S	W	H	I	15	18	1910	1881	16	1503	1281
Gm14.VB.65	Gm14	49710000	49711204	1204	0	W	W	S	S	W	H	W	0	0	2	2	0	4	0
Gm15.VB.1	Gm15	0	180000	180000	13	W	W	W	W	W	W	I	3	15	23	16	15	19	357
Gm15.VB.2	Gm15	180000	210000	30000	4	W	B	B	B	B	B	I	0	76	74	76	66	73	68
Gm15.VB.3	Gm15	210000	580000	370000	40	W	W	W	W	W	W	I	19	44	46	44	35	36	731
Gm15.VB.4	Gm15	580000	740000	160000	24	W	B	W	B	B	W	I	6	405	55	405	385	51	200
Gm15.VB.5	Gm15	740000	830000	90000	12	W	B	S	B	B	S	I	26	141	101	140	133	88	43
Gm15.VB.6	Gm15	830000	840000	10000	2	W	W	S	W	W	W	W	3	4	15	4	4	5	3
Gm15.VB.7	Gm15	840000	980000	140000	12	W	B	W	B	B	W	I	16	448	16	446	409	6	382
Gm15.VB.8	Gm15	980000	1070000	90000	8	W	B	B	B	B	B	I	10	234	237	217	205	212	196
Gm15.VB.9	Gm15	1070000	1590000	520000	63	W	W	W	W	W	W	I	59	571	582	553	520	535	1252
Gm15.VB.10	Gm15	1590000	1650000	60000	8	W	W	W	W	W	W	I	11	303	298	295	292	294	131
Gm15.VB.11	Gm15	1650000	1710000	60000	3	W	B	S	G	W	W	W	9	189	194	189	136	144	79
Gm15.VB.12	Gm15	1710000	1900000	190000	20	W	W	S	W	W	H	I	24	732	780	733	690	797	700
Gm15.VB.13	Gm15	1900000	3030000	1130000	135	W	W	W	W	W	W	I	121	2187	284	2162	1999	276	2152
Gm15.VB.14	Gm15	3030000	3040000	10000	1	W	W	S	W	D	H	W	7	56	37	57	54	33	18
Gm15.VB.15	Gm15	3040000	3560000	520000	53	W	W	W	W	W	W	I	86	602	196	583	515	407	1059
Gm15.VB.16	Gm15	3560000	4070000	510000	66	W	W	W	W	W	H	I	45	250	44	245	233	987	854
Gm15.VB.17	Gm15	4070000	4080000	10000	0	W	W	W	W	W	W	I	1	23	21	22	22	10	28
Gm15.VB.18	Gm15	4080000	4660000	580000	75	W	W	S	W	W	W	I	54	593	1591	594	537	250	1232
Gm15.VB.19	Gm15	4660000	5160000	500000	66	W	W	W	W	W	W	I	24	410	106	411	371	113	1162
Gm15.VB.20	Gm15	5160000	5190000	30000	3	W	W	S	W	W	W	I	0	0	99	0	0	4	57
Gm15.VB.21	Gm15	5190000	5220000	30000	5	W	W	S	W	W	S	I	10	10	199	10	1	185	129
Gm15.VB.22	Gm15	5220000	5340000	120000	13	W	W	W	W	W	H	I	11	11	14	9	11	392	283
Gm15.VB.23	Gm15	5340000	5500000	160000	18	W	W	W	W	W	W	I	9	7	15	7	9	13	263
Gm15.VB.24	Gm15	5500000	5700000	200000	24	W	W	W	W	W	H	I	12	11	13	11	8	788	560
Gm15.VB.25	Gm15	5700000	6010000	310000	31	W	W	W	W	W	W	I	31	38	41	31	18	26	635
Gm15.VB.26	Gm15	6010000	6020000	10000	2	W	W	S	W	W	W	I	0	0	13	0	0	2	10
Gm15.VB.27	Gm15	6020000	6090000	70000	7	W	W	S	W	W	S	I	1	1	249	1	1	239	172
Gm15.VB.28	Gm15	6090000	6630000	540000	58	W	W	W	W	W	W	I	20	22	37	22	15	26	1361
Gm15.VB.29	Gm15	6630000	7240000	610000	61	W	W	W	W	W	H	I	29	34	31	33	24	2024	1333
Gm15.VB.30	Gm15	7240000	7530000	290000	29	W	W	W	W	W	W	I	17	17	17	17	16	23	792
Gm15.VB.31	Gm15	7530000	8000000	470000	55	W	W	W	W	W	H	I	21	18	19	15	12	1497	1118
Gm15.VB.32	Gm15	8000000	8130000	130000	9	W	W	W	W	W	W	I	21	21	19	22	13	19	269
Gm15.VB.33	Gm15	8130000	8260000	130000	13	W	W	W	W	W	H	I	10	19	11	20	20	245	220
Gm15.VB.34	Gm15	8260000	8760000	500000	51	W	B	W	B	B	B	I	30	1451	25	1426	1338	1370	966
Gm15.VB.35	Gm15	8760000	8830000	70000	5	W	W	W	W	W	H	I	9	8	9	11	10	183	177
Gm15.VB.36	Gm15	8830000	9000000	170000	21	W	W	W	W	W	W	I	20	25	20	21	20	15	328

Gm15.VB.37	Gm15	9000000	9050000	50000	6	W	B	W	B	W	W	I	0	203	0	210	3	2	129
Gm15.VB.38	Gm15	9050000	9070000	20000	1	W	B	S	B	S	W	I	0	17	14	17	13	1	44
Gm15.VB.39	Gm15	9070000	10070000	1000000	100	W	B	B	B	B	H	I	59	4021	4121	3983	3680	3644	2618
Gm15.VB.40	Gm15	10070000	10150000	80000	8	W	B	W	B	W	H	I	12	297	15	294	5	335	155
Gm15.VB.41	Gm15	10150000	10250000	100000	6	W	W	W	W	W	W	I	5	11	9	9	6	9	301
Gm15.VB.42	Gm15	10250000	10340000	90000	9	W	B	W	B	W	B	I	9	354	9	360	17	328	178
Gm15.VB.43	Gm15	10340000	10430000	90000	7	W	B	B	B	D	D	I	45	542	554	554	482	497	74
Gm15.VB.44	Gm15	10430000	11240000	810000	68	W	B	W	B	W	B	I	88	2570	97	2544	60	2328	1779
Gm15.VB.45	Gm15	11240000	11440000	200000	16	W	B	S	B	W	B	I	18	498	584	500	58	453	702
Gm15.VB.46	Gm15	11440000	11530000	90000	7	W	W	S	W	W	W	I	18	21	263	20	14	20	213
Gm15.VB.47	Gm15	11530000	11880000	350000	26	W	W	W	W	W	W	I	23	38	22	40	16	36	1090
Gm15.VB.48	Gm15	11880000	11940000	60000	5	W	W	S	W	W	W	I	9	11	231	10	9	11	232
Gm15.VB.49	Gm15	11940000	12040000	100000	12	W	W	W	W	W	W	I	3	4	16	4	3	4	539
Gm15.VB.50	Gm15	12040000	12250000	210000	15	W	B	B	B	D	B	I	8	799	783	791	608	757	714
Gm15.VB.51	Gm15	12250000	12820000	570000	40	W	B	W	B	W	B	I	30	2074	28	2032	33	1971	1515
Gm15.VB.52	Gm15	12820000	15960000	3140000	164	W	B	S	B	D	B	I	246	20635	19290	20326	15437	19539	13082
Gm15.VB.53	Gm15	15960000	16790000	830000	30	W	W	W	W	W	W	I	42	92	88	92	92	85	2091
Gm15.VB.54	Gm15	16790000	17260000	470000	21	W	B	W	B	W	B	I	40	4104	77	4036	60	3891	3257
Gm15.VB.55	Gm15	17260000	20430000	3170000	70	W	W	W	W	W	W	I	136	260	256	266	229	217	10779
Gm15.VB.56	Gm15	20430000	20510000	80000	0	W	W	S	W	S	W	I	1	4	134	4	126	0	939
Gm15.VB.57	Gm15	20510000	34640000	14130000	179	W	B	B	B	B	W	I	622	46790	47252	46077	46745	567	16680
Gm15.VB.58	Gm15	34640000	34750000	110000	0	W	W	W	W	W	W	W	50	11	14	15	21	39	38
Gm15.VB.59	Gm15	34750000	44110000	9360000	154	W	B	B	B	B	W	I	526	42817	43253	42186	41725	536	17877
Gm15.VB.60	Gm15	44110000	45670000	1560000	35	W	W	W	W	W	W	I	207	256	257	234	246	204	3901
Gm15.VB.61	Gm15	45670000	45680000	10000	1	W	B	W	W	B	W	I	0	7	3	4	7	0	15
Gm15.VB.62	Gm15	45680000	46930000	1250000	38	W	B	B	B	B	W	I	75	6765	7113	6690	6548	87	4097
Gm15.VB.63	Gm15	46930000	46990000	60000	0	W	B	B	B	B	B	I	6	293	293	288	296	288	241
Gm15.VB.64	Gm15	46990000	47740000	750000	47	W	W	W	W	W	W	I	53	69	72	71	58	63	3480
Gm15.VB.65	Gm15	47740000	47790000	50000	3	W	B	W	B	W	W	I	1	225	6	214	5	5	132
Gm15.VB.66	Gm15	47790000	47810000	20000	1	W	B	S	B	S	W	I	1	76	13	71	13	1	85
Gm15.VB.67	Gm15	47810000	47840000	30000	3	W	W	W	W	W	W	I	0	2	2	2	2	3	118
Gm15.VB.68	Gm15	47840000	47960000	120000	9	W	W	S	W	S	S	I	0	9	569	8	536	539	654
Gm15.VB.69	Gm15	47960000	48130000	170000	5	W	B	S	B	S	H	I	5	731	1124	722	1067	859	481
Gm15.VB.70	Gm15	48130000	48230000	100000	5	W	B	S	B	S	W	I	7	512	113	480	101	14	373
Gm15.VB.71	Gm15	48230000	48320000	90000	1	W	B	B	B	B	B	I	3	332	324	325	295	305	230
Gm15.VB.72	Gm15	48320000	48580000	260000	18	W	W	S	W	S	W	I	48	58	803	54	742	48	884
Gm15.VB.73	Gm15	48580000	48970000	390000	20	W	B	S	B	S	B	I	1	2404	2222	2395	2074	2319	1619
Gm15.VB.74	Gm15	48970000	49290000	320000	15	W	W	S	W	S	W	I	22	37	1867	37	1762	25	1134
Gm15.VB.75	Gm15	49290000	49470000	180000	6	W	B	B	B	B	H	I	13	1419	1379	1393	1318	728	750
Gm15.VB.76	Gm15	49470000	49580000	110000	9	W	B	B	B	B	W	I	0	465	500	468	462	5	333
Gm15.VB.77	Gm15	49580000	49630000	50000	4	W	W	S	W	S	W	I	0	4	351	4	301	2	175
Gm15.VB.78	Gm15	49630000	49690000	60000	4	W	W	W	W	W	W	I	2	3	6	1	5	4	390
Gm15.VB.79	Gm15	49690000	49760000	70000	6	W	W	W	W	W	H	I	7	9	11	9	11	147	146
Gm15.VB.80	Gm15	49760000	49930000	170000	11	W	W	S	W	S	H	I	13	14	1015	14	932	616	658
Gm15.VB.81	Gm15	49930000	50180000	250000	14	W	W	W	W	W	H	I	7	8	7	9	21	921	895
Gm15.VB.82	Gm15	50180000	50430000	250000	17	W	B	W	B	W	H	I	7	1905	8	1876	6	1582	1062
Gm15.VB.83	Gm15	50430000	50930000	500000	24	W	B	W	B	W	W	I	51	2129	54	2126	46	53	2357
Gm15.VB.84	Gm15	50930000	50939160	9160	0	W	B	W	B	W	W	I	11	91	10	85	9	11	78
Gm16.VB.1	Gm16	0	250000	250000	34	W	B	W	B	B	W	I	11	304	8	296	286	8	404
Gm16.VB.2	Gm16	250000	350000	100000	5	W	W	W	W	W	W	I	0	11	0	11	11	0	318
Gm16.VB.3	Gm16	350000	490000	140000	10	W	B	W	B	B	W	I	13	535	13	539	515	8	383
Gm16.VB.4	Gm16	490000	630000	140000	12	W	W	W	W	W	W	I	3	12	3	11	14	3	270
Gm16.VB.5	Gm16	630000	680000	50000	7	W	B	W	B	B	W	I	3	129	3	121	115	3	87
Gm16.VB.6	Gm16	680000	810000	130000	9	W	B	W	B	W	W	I	10	610	10	595	15	8	329
Gm16.VB.7	Gm16	810000	980000	170000	19	W	W	W	W	W	W	I	27	46	29	49	32	47	240
Gm16.VB.8	Gm16	980000	990000	10000	2	W	B	W	B	W	B	I	1	21	1	21	1	20	17
Gm16.VB.9	Gm16	990000	1100000	110000	11	W	W	W	W	W	W	I	10	10	10	11	7	7	207
Gm16.VB.10	Gm16	1100000	1240000	140000	14	W	B	W	B	W	W	I	4	192	4	180	4	16	349
Gm16.VB.11	Gm16	1240000	1320000	80000	7	W	B	W	B	W	H	I	6	50	7	51	6	147	200
Gm16.VB.12	Gm16	1320000	1890000	570000	47	W	B	W	B	B	H	I	13	2597	17	2564	2365	1829	1279
Gm16.VB.13	Gm16	1890000	1960000	70000	3	W	B	W	B	B	W	I	11	307	11	297	300	14	237
Gm16.VB.14	Gm16	1960000	2240000	280000	24	W	W	W	W	W	W	I	54	63	53	64	58	50	584
Gm16.VB.15	Gm16	2240000	2300000	60000	2	W	B	W	B	B	W	I	5	106	6	106	96	5	90
Gm16.VB.16	Gm16	2300000	2660000	360000	33	W	B	W	B	B	H	I	27	1106	25	1101	1005	714	933
Gm16.VB.17	Gm16	2660000	2760000	100000	13	W	W	W	W	W	H	I	16	19	16	19	19	64	166
Gm16.VB.18	Gm16	2760000	3370000	610000	63	W	B	W	B	B	H	I	25	2755	23	2701	2450	1242	1809
Gm16.VB.19	Gm16	3370000	3520000	150000	11	W	B	W	B	D	W	I	6	196	4	194	583	5	358
Gm16.VB.20	Gm16	3520000	4060000	540000	53	W	B	W	B	B	H	I	45	2091	41	2059	1888	1394	1177
Gm16.VB.21	Gm16	4060000	4250000	190000	15	W	W	W	W	W	W	I	14	33	16	34	32	23	541
Gm16.VB.22	Gm16	4250000	4340000	90000	11	W	B	W	B	B	H	I	15	294	14	290	280	336	274
Gm16.VB.23	Gm16	4340000	4750000	410000	27	W	W	W	W	W	H	I	41	57	42	61	54	946	1142
Gm16.VB.24	Gm16	4750000	4920000	170000	17	W	B	W	B	B	H	I	5	448	5	449	416	372	415
Gm16.VB.25	Gm16	4920000	5000000	80000	5	W	B	W	B	W	W	I	9	148	9	143	12	37	129
Gm16.VB.26	Gm16	5000000	5310000	310000	20	W	W	W	W	W	H	I	13	29	18	27	16	472	815
Gm16.VB.27	Gm16	5310000	5430000	120000	11	W	B	W	B	W	H	I	12	694	13	685	13	412	379
Gm16.VB.28	Gm16	5430000	5600000	170000	15	W	B	W	B	D	H	I	30	1032	28	1020	922	510	636
Gm16.VB.29	Gm16	5600000	5940000	340000	31	W	B	W	B	W	H	I	60	2561	58	2517	58	1026	1061
Gm16.VB.30	Gm16	5940000	6020000	80000	8	W	B	W	B	W	W	I	5	376	5	362	6	7	214

Gm16.VB.31	Gm16	6020000	6060000	40000	4	W	W	W	W	D	W	I	1	4	1	4	172	6	93
Gm16.VB.32	Gm16	6060000	6660000	600000	42	W	B	W	B	D	H	I	93	3116	108	3068	3258	2946	2370
Gm16.VB.33	Gm16	6660000	7240000	580000	38	W	W	W	W	W	W	I	67	69	79	66	53	74	2201
Gm16.VB.34	Gm16	7240000	9790000	2550000	85	W	W	S	W	W	H	I	140	135	13130	130	121	7433	8127
Gm16.VB.35	Gm16	9790000	9970000	180000	5	W	W	S	W	W	W	I	12	13	93	21	20	39	398
Gm16.VB.36	Gm16	9970000	14710000	4740000	49	W	W	S	W	W	H	I	243	252	10131	219	216	6029	6134
Gm16.VB.37	Gm16	14710000	14900000	190000	2	W	W	S	W	W	W	I	11	14	240	11	13	39	194
Gm16.VB.38	Gm16	14900000	16800000	1900000	19	W	W	S	W	W	H	I	95	93	5599	87	113	855	4435
Gm16.VB.39	Gm16	16800000	17370000	570000	6	W	W	S	W	W	W	I	60	58	1249	55	61	224	1047
Gm16.VB.40	Gm16	17370000	18630000	1260000	16	W	W	W	W	W	W	I	56	49	57	50	51	206	2987
Gm16.VB.41	Gm16	18630000	18680000	50000	1	W	W	S	W	W	W	I	0	1	25	1	1	7	162
Gm16.VB.42	Gm16	18680000	19530000	850000	26	W	W	S	W	W	H	I	42	45	4794	45	36	1112	3036
Gm16.VB.43	Gm16	19530000	21100000	1570000	19	W	W	S	W	W	W	I	75	64	1151	61	77	283	4194
Gm16.VB.44	Gm16	21100000	22060000	960000	27	W	W	S	W	W	H	I	66	72	2727	71	59	485	2184
Gm16.VB.45	Gm16	22060000	22640000	580000	2	W	W	S	W	W	W	I	28	27	1305	27	29	214	1099
Gm16.VB.46	Gm16	22640000	23690000	1050000	7	W	W	W	W	W	W	I	34	41	46	37	37	173	2093
Gm16.VB.47	Gm16	23690000	25290000	1600000	29	W	W	W	W	W	H	I	68	50	73	58	62	1345	6875
Gm16.VB.48	Gm16	25290000	25420000	130000	3	W	W	W	W	W	W	I	4	5	4	5	5	34	820
Gm16.VB.49	Gm16	25420000	28720000	3300000	138	W	W	W	W	W	H	I	177	199	179	184	201	13201	16193
Gm16.VB.50	Gm16	28720000	28970000	250000	15	W	B	W	B	B	B	I	30	1788	30	1725	1604	1652	1132
Gm16.VB.51	Gm16	28970000	29070000	100000	6	W	B	W	B	D	W	I	3	552	4	537	532	11	241
Gm16.VB.52	Gm16	29070000	29330000	260000	18	W	B	W	B	B	H	I	36	1944	39	1907	1787	1687	708
Gm16.VB.53	Gm16	29330000	29860000	530000	29	W	W	W	W	W	W	I	20	34	20	36	32	45	2729
Gm16.VB.54	Gm16	29860000	30160000	300000	18	W	B	W	B	B	B	I	15	1605	19	1564	1443	1474	842
Gm16.VB.55	Gm16	30160000	30270000	110000	7	W	W	W	W	W	W	I	10	15	10	15	14	13	495
Gm16.VB.56	Gm16	30270000	30330000	60000	5	W	B	W	B	B	W	I	0	399	0	377	369	4	211
Gm16.VB.57	Gm16	30330000	30340000	10000	2	W	B	W	B	B	H	I	0	13	0	15	14	21	24
Gm16.VB.58	Gm16	30340000	30900000	560000	47	W	W	W	W	W	H	I	67	65	60	62	51	2627	2232
Gm16.VB.59	Gm16	30900000	31080000	180000	17	W	W	W	W	W	W	I	16	15	15	15	10	14	764
Gm16.VB.60	Gm16	31080000	31620000	540000	51	W	W	W	W	W	H	I	25	22	22	21	22	2790	1709
Gm16.VB.61	Gm16	31620000	31830000	210000	21	W	W	W	W	W	W	I	14	18	14	13	14	19	650
Gm16.VB.62	Gm16	31830000	31910000	80000	6	W	B	W	W	D	D	I	24	416	28	22	287	284	158
Gm16.VB.63	Gm16	31910000	33400000	1490000	112	W	W	W	W	W	H	I	104	103	108	100	89	4410	3386
Gm16.VB.64	Gm16	33400000	33530000	130000	21	W	W	W	W	W	W	I	8	7	8	7	8	5	556
Gm16.VB.65	Gm16	33530000	33570000	40000	2	W	W	W	W	W	H	I	0	1	0	0	0	239	153
Gm16.VB.66	Gm16	33570000	35490000	1920000	156	W	W	W	W	W	W	I	66	76	78	69	80	85	3387
Gm16.VB.67	Gm16	35490000	35530000	40000	2	W	B	W	B	W	W	I	8	90	7	92	1	2	107
Gm16.VB.68	Gm16	35530000	35580000	50000	6	W	B	W	B	B	W	I	0	190	0	182	178	0	148
Gm16.VB.69	Gm16	35580000	35750000	170000	14	W	W	W	W	W	W	I	16	20	16	19	15	13	476
Gm16.VB.70	Gm16	35750000	36650000	900000	114	W	B	W	B	B	W	I	27	5017	26	4890	4544	19	2760
Gm16.VB.71	Gm16	36650000	37030000	380000	38	W	B	W	B	B	H	I	21	2104	22	2064	1916	1191	1057
Gm16.VB.72	Gm16	37030000	37390000	360000	39	W	B	W	B	B	W	I	21	1148	25	1102	1091	19	767
Gm16.VB.73	Gm16	37390000	37397385	7385	0	W	B	W	W	W	W	I	0	3	0	2	2	0	18
Gm17.VB.1	Gm17	0	340000	340000	24	W	W	W	W	W	W	I	14	19	10	12	8	11	633
Gm17.VB.2	Gm17	340000	800000	460000	56	W	W	W	W	W	H	I	14	21	12	10	8	1567	951
Gm17.VB.3	Gm17	800000	1320000	520000	65	W	W	W	W	W	W	I	12	30	12	13	12	24	1166
Gm17.VB.4	Gm17	1320000	1420000	100000	12	W	W	W	W	W	H	I	5	10	5	5	5	280	283
Gm17.VB.5	Gm17	1420000	1470000	50000	6	W	B	W	W	W	H	I	7	108	7	7	6	193	76
Gm17.VB.6	Gm17	1470000	1580000	110000	14	W	W	W	W	W	H	I	13	17	12	15	13	96	223
Gm17.VB.7	Gm17	1580000	1990000	410000	49	W	W	W	W	W	W	I	34	33	31	31	26	55	480
Gm17.VB.8	Gm17	1990000	2010000	20000	2	W	W	W	W	W	H	I	6	6	6	6	6	55	12
Gm17.VB.9	Gm17	2010000	2150000	140000	16	W	W	W	W	W	W	I	12	11	11	6	9	19	289
Gm17.VB.10	Gm17	2150000	2340000	190000	23	W	W	W	W	W	H	I	7	13	7	13	6	278	308
Gm17.VB.11	Gm17	2340000	2510000	170000	26	W	B	W	B	W	B	I	3	719	3	717	5	683	549
Gm17.VB.12	Gm17	2510000	3000000	490000	57	W	W	W	W	W	W	I	2	22	3	21	2	12	739
Gm17.VB.13	Gm17	3000000	3440000	440000	48	W	B	W	W	W	B	I	14	712	14	27	14	708	806
Gm17.VB.14	Gm17	3440000	3510000	70000	7	W	B	W	B	W	B	I	15	212	9	210	7	187	131
Gm17.VB.15	Gm17	3510000	3600000	90000	7	W	W	W	W	W	W	I	10	19	11	16	5	18	149
Gm17.VB.16	Gm17	3600000	3660000	60000	4	W	W	S	S	S	W	I	5	5	253	256	235	6	159
Gm17.VB.17	Gm17	3660000	3880000	220000	23	W	W	W	W	W	W	I	16	24	19	24	13	13	499
Gm17.VB.18	Gm17	3880000	4480000	600000	74	W	B	W	B	W	W	I	16	675	16	718	9	26	910
Gm17.VB.19	Gm17	4480000	4510000	30000	4	W	W	W	W	W	W	I	19	21	17	18	0	0	56
Gm17.VB.20	Gm17	4510000	4730000	220000	23	W	W	W	W	W	W	I	11	24	11	26	10	10	395
Gm17.VB.21	Gm17	4730000	4990000	260000	30	W	B	W	B	W	W	I	6	601	10	602	7	7	494
Gm17.VB.22	Gm17	4990000	5070000	80000	7	W	B	W	W	W	W	I	4	178	4	9	4	4	114
Gm17.VB.23	Gm17	5070000	5170000	100000	11	W	W	W	W	W	W	I	1	7	3	3	3	1	168
Gm17.VB.24	Gm17	5170000	5230000	60000	6	W	W	S	S	S	W	I	3	5	154	145	136	3	98
Gm17.VB.25	Gm17	5230000	5290000	60000	5	W	W	W	W	W	W	I	2	10	3	5	2	2	107
Gm17.VB.26	Gm17	5290000	5340000	50000	7	W	B	W	W	W	W	I	0	241	1	1	1	0	23
Gm17.VB.27	Gm17	5340000	5720000	380000	35	W	W	W	W	W	W	I	27	47	36	41	31	26	526
Gm17.VB.28	Gm17	5720000	5800000	80000	10	W	W	W	W	W	H	I	2	6	2	7	1	121	164
Gm17.VB.29	Gm17	5800000	6180000	380000	42	W	B	W	G	W	W	I	24	1388	24	863	26	23	845
Gm17.VB.30	Gm17	6180000	6220000	40000	7	W	W	W	G	W	W	I	0	2	0	115	0	1	53
Gm17.VB.31	Gm17	6220000	6600000	380000	44	W	W	W	W	W	W	I	8	18	6	30	8	7	725
Gm17.VB.32	Gm17	6600000	6890000	290000	36	W	B	W	W	W	W	I	8	888	8	51	7	8	522
Gm17.VB.33	Gm17	6890000	7280000	390000	38	W	W	W	W	W	W	I	13	24	12	32	13	29	719
Gm17.VB.34	Gm17	7280000	7400000	120000	14	W	W	W	G	W	G	I	2	4	2	116	1	110	299
Gm17.VB.35	Gm17	7400000	7560000	160000	17	W	W	W	W	W	H	I	2	4	2	6	1	263	204

Gm17.VB.36	Gm17	7560000	7750000	190000	20	W	W	W	W	W	W	I	2	2	2	2	2	2	317
Gm17.VB.37	Gm17	7750000	7790000	40000	2	W	B	W	B	W	B	I	0	78	0	78	0	78	48
Gm17.VB.38	Gm17	7790000	8330000	540000	60	W	B	W	B	B	B	I	23	883	26	863	771	879	1173
Gm17.VB.39	Gm17	8330000	8640000	310000	36	W	B	B	B	B	B	I	32	964	986	956	901	929	678
Gm17.VB.40	Gm17	8640000	8780000	140000	18	W	B	W	B	B	W	I	2	153	11	153	139	10	268
Gm17.VB.41	Gm17	8780000	9070000	290000	24	W	B	W	B	B	H	I	26	1197	40	1181	1066	882	542
Gm17.VB.42	Gm17	9070000	9290000	220000	21	W	B	S	B	B	H	I	9	628	618	615	592	203	410
Gm17.VB.43	Gm17	9290000	9490000	200000	27	W	W	S	W	W	W	I	4	11	384	7	10	7	456
Gm17.VB.44	Gm17	9490000	9640000	150000	15	W	B	B	B	B	W	I	5	408	423	419	399	14	229
Gm17.VB.45	Gm17	9640000	9690000	50000	5	W	B	B	B	B	B	I	1	63	62	64	60	58	66
Gm17.VB.46	Gm17	9690000	9750000	60000	2	W	W	W	W	W	W	I	2	7	7	7	7	4	65
Gm17.VB.47	Gm17	9750000	11030000	1280000	126	W	W	W	W	W	W	I	92	127	155	146	95	114	2614
Gm17.VB.48	Gm17	11030000	11170000	140000	15	W	W	W	W	W	W	I	3	3	59	58	3	7	451
Gm17.VB.49	Gm17	11170000	11270000	100000	8	W	W	W	W	W	W	I	1	1	10	10	1	5	183
Gm17.VB.50	Gm17	11270000	11290000	20000	1	W	W	S	S	W	W	I	0	0	155	151	0	2	22
Gm17.VB.51	Gm17	11290000	11400000	110000	12	W	W	S	S	W	H	I	13	11	602	579	12	461	343
Gm17.VB.52	Gm17	11400000	11610000	210000	13	W	W	S	S	W	W	I	2	2	432	435	2	8	454
Gm17.VB.53	Gm17	11610000	11650000	40000	3	W	W	W	W	W	W	I	7	7	10	10	6	9	76
Gm17.VB.54	Gm17	11650000	11750000	100000	12	W	W	W	W	W	H	I	19	22	24	24	19	318	218
Gm17.VB.55	Gm17	11750000	11900000	150000	16	W	B	S	S	B	W	I	5	904	782	755	822	2	540
Gm17.VB.56	Gm17	11900000	12100000	200000	15	W	W	S	S	W	W	I	7	21	1096	1040	18	7	572
Gm17.VB.57	Gm17	12100000	13390000	1290000	91	W	B	B	B	B	W	I	58	7552	7852	7621	6825	46	4822
Gm17.VB.58	Gm17	13390000	13490000	100000	3	W	B	W	W	B	W	I	8	193	20	18	180	4	218
Gm17.VB.59	Gm17	13490000	13500000	10000	1	W	B	S	S	W	W	I	1	6	36	35	3	1	17
Gm17.VB.60	Gm17	13500000	13650000	150000	8	W	W	S	S	W	W	I	6	13	381	354	13	6	545
Gm17.VB.61	Gm17	13650000	13670000	20000	2	W	B	B	B	B	W	I	0	80	80	80	80	0	104
Gm17.VB.62	Gm17	13670000	13950000	280000	15	W	W	W	W	W	W	I	30	44	50	43	43	28	701
Gm17.VB.63	Gm17	13950000	14400000	450000	29	W	W	S	S	W	W	I	25	39	2538	2437	26	19	1269
Gm17.VB.64	Gm17	14400000	15050000	650000	21	W	B	S	S	B	W	I	27	2959	3403	3313	2876	34	2362
Gm17.VB.65	Gm17	15050000	15460000	410000	17	W	W	S	S	W	W	I	61	66	2193	2171	46	36	1708
Gm17.VB.66	Gm17	15460000	15720000	260000	7	W	B	S	S	B	W	I	21	1165	1138	1075	1166	13	1394
Gm17.VB.67	Gm17	15720000	29860000	14140000	179	W	W	S	S	W	W	I	790	1555	43240	42159	1499	1380	20687
Gm17.VB.68	Gm17	29860000	29930000	70000	1	W	W	W	W	W	W	W	0	2	11	19	0	2	10
Gm17.VB.69	Gm17	29930000	30000000	70000	0	W	W	W	W	W	W	I	3	6	3	5	2	3	123
Gm17.VB.70	Gm17	30000000	30040000	40000	0	W	W	W	W	W	W	W	22	22	17	10	24	31	6
Gm17.VB.71	Gm17	30040000	31690000	1650000	24	W	W	S	S	W	W	I	68	183	5818	5703	162	161	4254
Gm17.VB.72	Gm17	31690000	31850000	160000	5	W	W	S	S	W	W	I	2	58	426	403	70	68	102
Gm17.VB.73	Gm17	31850000	33710000	1860000	51	W	W	S	S	W	W	I	76	159	7864	7719	137	138	3676
Gm17.VB.74	Gm17	33710000	33970000	260000	6	W	B	S	S	B	B	I	22	844	1371	1295	777	793	897
Gm17.VB.75	Gm17	33970000	34490000	520000	13	W	W	S	S	W	W	I	11	45	1767	1723	37	41	1681
Gm17.VB.76	Gm17	34490000	34960000	470000	13	W	B	S	S	B	B	I	25	1312	1638	1649	1252	1272	1468
Gm17.VB.77	Gm17	34960000	35120000	160000	4	W	W	S	S	W	W	I	0	23	925	926	20	23	403
Gm17.VB.78	Gm17	35120000	35320000	200000	11	W	B	S	S	B	B	I	9	548	1307	1287	521	521	747
Gm17.VB.79	Gm17	35320000	35470000	150000	4	W	W	S	S	W	W	I	4	38	178	176	33	39	914
Gm17.VB.80	Gm17	35470000	36850000	1380000	44	W	B	S	S	B	B	I	121	3310	5639	5412	3209	3301	4780
Gm17.VB.81	Gm17	36850000	37220000	370000	12	W	B	W	W	B	B	I	44	2768	83	69	2685	2695	1931
Gm17.VB.82	Gm17	37220000	37290000	70000	2	W	W	W	W	W	W	I	0	4	4	3	5	5	275
Gm17.VB.83	Gm17	37290000	37380000	90000	4	W	W	S	S	W	W	I	1	6	353	324	7	6	278
Gm17.VB.84	Gm17	37380000	37400000	20000	2	W	B	S	S	B	B	I	2	33	83	82	20	26	80
Gm17.VB.85	Gm17	37400000	37420000	20000	1	W	W	S	S	W	W	I	0	0	75	75	0	0	36
Gm17.VB.86	Gm17	37420000	37740000	320000	21	W	W	W	W	W	W	I	23	30	37	35	25	18	513
Gm17.VB.87	Gm17	37740000	37750000	10000	1	W	W	W	W	W	H	I	0	0	1	1	0	7	19
Gm17.VB.88	Gm17	37750000	37910000	160000	8	W	W	S	S	W	H	I	14	14	435	419	7	314	505
Gm17.VB.89	Gm17	37910000	38100000	190000	20	W	W	W	W	W	W	I	3	6	19	19	3	1	926
Gm17.VB.90	Gm17	38100000	38790000	690000	45	W	W	S	S	W	W	I	80	78	3195	3115	70	69	2301
Gm17.VB.91	Gm17	38790000	39030000	240000	21	W	W	W	W	W	W	I	21	21	30	29	14	11	813
Gm17.VB.92	Gm17	39030000	39070000	40000	2	W	W	S	S	S	W	I	0	1	173	174	155	0	131
Gm17.VB.93	Gm17	39070000	39080000	10000	1	W	W	W	W	W	W	I	1	2	4	4	4	1	23
Gm17.VB.94	Gm17	39080000	39400000	320000	29	W	B	W	W	W	W	I	32	793	46	42	40	28	688
Gm17.VB.95	Gm17	39400000	39500000	100000	9	W	B	S	S	S	W	I	48	549	415	400	356	42	340
Gm17.VB.96	Gm17	39500000	39720000	220000	17	W	W	S	S	S	W	I	27	27	1381	1322	1228	23	516
Gm17.VB.97	Gm17	39720000	39910000	190000	16	W	W	W	W	W	W	I	9	8	14	14	14	7	593
Gm17.VB.98	Gm17	39910000	40050000	140000	10	W	W	S	S	S	W	I	4	4	514	501	486	3	366
Gm17.VB.99	Gm17	40050000	40110000	60000	4	W	W	S	S	W	W	I	11	11	164	162	11	11	103
Gm17.VB.100	Gm17	40110000	40210000	100000	11	W	W	W	W	W	W	I	4	3	7	5	4	3	277
Gm17.VB.101	Gm17	40210000	40220000	10000	2	W	W	S	S	W	W	I	0	4	5	5	4	0	28
Gm17.VB.102	Gm17	40220000	40500000	280000	23	W	B	B	B	B	W	I	36	804	766	749	699	23	461
Gm17.VB.103	Gm17	40500000	40510000	10000	0	W	B	W	W	B	W	I	1	6	1	1	6	1	38
Gm17.VB.104	Gm17	40510000	40640000	130000	12	W	W	W	W	W	W	I	3	7	10	10	7	3	358
Gm17.VB.105	Gm17	40640000	40780000	140000	13	W	B	W	W	B	W	I	6	177	13	13	138	3	376
Gm17.VB.106	Gm17	40780000	41060000	280000	35	W	B	B	B	B	W	I	7	542	545	549	511	5	789
Gm17.VB.107	Gm17	41060000	41080000	20000	0	W	B	B	B	B	B	I	0	95	97	97	94	82	31
Gm17.VB.108	Gm17	41080000	41180000	100000	12	W	B	B	B	B	W	I	7	55	56	53	52	9	228
Gm17.VB.109	Gm17	41180000	41240000	60000	6	W	B	B	B	B	B	I	0	170	169	168	158	144	74
Gm17.VB.110	Gm17	41240000	41390000	150000	11	W	B	B	B	B	W	I	10	327	332	327	308	26	469
Gm17.VB.111	Gm17	41390000	41520000	130000	12	W	B	B	B	B	H	I	6	580	585	576	535	304	263
Gm17.VB.112	Gm17	41520000	41660000	140000	13	W	B	B	B	B	W	I	2	71	72	72	66	5	240
Gm17.VB.113	Gm17	41660000	41720000	60000	7	W	B	B	B	B	B	I	4	206	206	195	195	198	56

Gm17.VB.114	Gm17	41720000	41790000	70000	11	W	W	W	W	W	H	I	0	3	2	3	1	124	106
Gm17.VB.115	Gm17	41790000	41900000	110000	10	W	W	W	W	W	W	I	5	15	17	19	11	42	174
Gm17.VB.116	Gm17	41900000	41906774	6774	0	W	W	W	W	W	W	I	11	11	11	12	9	11	0
Gm18.VB.1	Gm18	0	150000	150000	16	W	W	W	W	W	W	W	0	6	0	6	2	9	17
Gm18.VB.2	Gm18	150000	270000	120000	16	W	W	W	W	W	H	I	5	15	5	14	6	203	209
Gm18.VB.3	Gm18	270000	480000	210000	28	W	B	W	B	W	H	I	12	431	12	422	9	178	416
Gm18.VB.4	Gm18	480000	510000	30000	4	W	B	W	B	W	W	I	3	54	2	53	2	2	77
Gm18.VB.5	Gm18	510000	590000	80000	12	W	W	W	W	W	W	I	1	1	1	1	1	4	358
Gm18.VB.6	Gm18	590000	660000	70000	8	W	W	W	W	W	H	I	3	5	3	5	3	130	126
Gm18.VB.7	Gm18	660000	920000	260000	36	W	B	W	B	W	H	I	5	695	5	694	5	689	576
Gm18.VB.8	Gm18	920000	1070000	150000	19	W	B	W	B	W	W	I	15	236	16	220	18	23	282
Gm18.VB.9	Gm18	1070000	2010000	940000	106	W	B	W	B	W	H	I	60	3283	62	3228	41	3144	1800
Gm18.VB.10	Gm18	2010000	2580000	570000	65	W	B	W	B	B	H	I	39	1729	42	1700	1558	1884	1353
Gm18.VB.11	Gm18	2580000	2700000	120000	13	W	B	W	B	B	W	I	20	403	15	393	361	28	179
Gm18.VB.12	Gm18	2700000	2920000	220000	23	W	B	W	B	B	H	I	10	396	11	382	356	435	505
Gm18.VB.13	Gm18	2920000	3030000	110000	4	W	W	W	W	W	W	I	6	16	6	13	13	8	258
Gm18.VB.14	Gm18	3030000	3280000	250000	21	W	B	W	B	B	B	I	4	947	3	940	891	903	794
Gm18.VB.15	Gm18	3280000	3390000	110000	8	W	W	W	W	W	W	I	14	25	16	24	21	17	371
Gm18.VB.16	Gm18	3390000	3490000	100000	7	W	W	W	W	W	H	I	30	27	31	31	26	412	81
Gm18.VB.17	Gm18	3490000	4320000	830000	74	W	W	W	W	W	W	I	56	68	60	62	62	58	2488
Gm18.VB.18	Gm18	4320000	4520000	200000	17	W	B	W	B	B	W	I	15	719	17	720	656	16	536
Gm18.VB.19	Gm18	4520000	4620000	100000	11	W	W	W	W	W	W	I	9	17	9	17	16	7	364
Gm18.VB.20	Gm18	4620000	5200000	580000	53	W	B	W	B	B	W	I	57	2227	53	2196	2035	57	1609
Gm18.VB.21	Gm18	5200000	5410000	210000	11	W	W	W	W	W	W	I	30	35	26	30	24	16	730
Gm18.VB.22	Gm18	5410000	5610000	200000	14	W	B	W	B	B	W	I	21	809	23	800	687	20	620
Gm18.VB.23	Gm18	5610000	5910000	300000	25	W	W	W	W	W	W	I	70	88	77	91	74	52	1450
Gm18.VB.24	Gm18	5910000	6030000	120000	7	W	B	W	B	B	W	I	6	444	6	447	375	5	121
Gm18.VB.25	Gm18	6030000	6400000	370000	21	W	W	W	W	W	W	I	45	57	37	44	54	42	726
Gm18.VB.26	Gm18	6400000	6670000	270000	15	W	W	W	W	W	H	I	8	12	8	13	12	765	581
Gm18.VB.27	Gm18	6670000	6950000	280000	16	W	B	W	B	B	H	I	19	1306	16	1304	1185	1532	1026
Gm18.VB.28	Gm18	6950000	7050000	100000	9	W	B	W	B	B	W	I	1	507	2	502	459	4	319
Gm18.VB.29	Gm18	7050000	7070000	20000	2	W	B	W	G	D	H	I	2	199	2	179	159	66	47
Gm18.VB.30	Gm18	7070000	9140000	2070000	116	W	B	W	B	B	W	I	143	10747	147	10595	10021	149	7640
Gm18.VB.31	Gm18	9140000	9210000	70000	3	W	W	W	W	D	W	I	11	29	11	8	499	8	306
Gm18.VB.32	Gm18	9210000	9240000	30000	3	W	W	W	W	D	W	I	0	6	0	0	181	0	274
Gm18.VB.33	Gm18	9240000	9670000	430000	21	W	W	W	W	W	W	I	21	36	19	20	25	17	1365
Gm18.VB.34	Gm18	9670000	10000000	330000	18	W	B	W	W	W	W	I	44	927	41	34	48	42	1384
Gm18.VB.35	Gm18	10000000	10030000	30000	2	W	B	W	W	D	W	I	0	33	0	0	38	0	107
Gm18.VB.36	Gm18	10030000	10230000	200000	9	W	W	W	W	W	W	I	9	24	8	8	18	8	453
Gm18.VB.37	Gm18	10230000	10770000	540000	20	W	B	W	W	B	W	I	7	476	19	7	482	12	1753
Gm18.VB.38	Gm18	10770000	10780000	10000	0	W	W	W	W	D	W	I	0	4	0	0	5	0	5
Gm18.VB.39	Gm18	10780000	11680000	900000	31	W	W	W	W	W	W	I	31	83	31	26	93	34	3570
Gm18.VB.40	Gm18	11680000	13870000	2190000	58	W	B	W	W	B	W	I	100	13054	102	76	12832	89	10583
Gm18.VB.41	Gm18	13870000	13990000	120000	0	W	W	W	W	W	W	W	52	69	57	49	49	64	73
Gm18.VB.42	Gm18	13990000	14150000	160000	0	W	W	W	W	W	W	W	39	53	47	46	35	40	88
Gm18.VB.43	Gm18	14150000	18430000	4280000	96	W	B	W	W	B	W	I	292	26368	313	267	25853	271	18887
Gm18.VB.44	Gm18	18430000	24710000	6280000	99	W	B	W	W	B	H	I	310	29511	308	275	29175	28338	19838
Gm18.VB.45	Gm18	24710000	24740000	30000	0	W	W	W	W	W	W	W	0	4	0	0	0	1	0
Gm18.VB.46	Gm18	24740000	25430000	690000	4	W	B	B	B	B	W	W	38	4542	4529	4425	4546	30	275
Gm18.VB.47	Gm18	25430000	25440000	10000	0	W	B	W	W	W	W	I	3	7	1	3	0	2	7
Gm18.VB.48	Gm18	25440000	25630000	190000	0	W	W	W	W	W	W	W	26	12	8	14	14	18	23
Gm18.VB.49	Gm18	25630000	25650000	20000	0	W	W	W	W	W	W	W	2	8	0	0	0	2	1
Gm18.VB.50	Gm18	25650000	25670000	20000	1	W	B	W	W	W	W	I	0	16	1	0	6	1	21
Gm18.VB.51	Gm18	25670000	27510000	1840000	24	W	B	B	B	B	W	I	156	2201	2199	2146	2184	152	1429
Gm18.VB.52	Gm18	27510000	27530000	20000	0	W	W	W	W	D	W	W	1	11	9	7	16	3	10
Gm18.VB.53	Gm18	27530000	27550000	20000	0	W	W	W	W	W	W	W	3	12	7	6	6	12	7
Gm18.VB.54	Gm18	27550000	31500000	3950000	29	W	B	W	W	B	H	I	180	9684	181	166	9811	10703	8258
Gm18.VB.55	Gm18	31500000	31670000	170000	1	W	B	W	W	B	W	W	0	417	0	0	413	7	21
Gm18.VB.56	Gm18	31670000	33490000	1820000	12	W	B	W	W	B	H	I	69	5150	60	56	5332	7221	5501
Gm18.VB.57	Gm18	33490000	33610000	120000	1	W	W	W	W	W	W	W	2	38	1	2	25	8	13
Gm18.VB.58	Gm18	33610000	36110000	2500000	21	W	B	W	W	B	H	I	132	6515	131	130	6508	6590	5650
Gm18.VB.59	Gm18	36110000	36220000	110000	0	W	W	W	W	W	W	W	5	13	3	3	11	8	2
Gm18.VB.60	Gm18	36220000	37430000	1210000	12	W	B	W	W	B	H	I	58	3459	48	51	3506	3592	2164
Gm18.VB.61	Gm18	37430000	37530000	100000	0	W	W	W	W	W	W	W	11	16	6	7	15	12	20
Gm18.VB.62	Gm18	37530000	39260000	1730000	25	W	B	W	W	B	H	I	82	5747	81	73	5757	6212	5480
Gm18.VB.63	Gm18	39260000	40160000	900000	31	W	B	W	W	B	W	I	28	3136	33	30	3043	91	1437
Gm18.VB.64	Gm18	40160000	40330000	170000	3	W	W	W	W	W	W	I	7	25	5	3	7	13	161
Gm18.VB.65	Gm18	40330000	40380000	50000	1	W	B	W	W	W	W	I	0	35	0	0	21	9	83
Gm18.VB.66	Gm18	40380000	40500000	120000	1	W	W	W	W	W	W	I	12	27	11	13	23	20	234
Gm18.VB.67	Gm18	40500000	41740000	1240000	22	W	B	W	W	B	W	I	84	2093	82	69	2139	145	1965
Gm18.VB.68	Gm18	41740000	42070000	330000	3	W	B	W	W	B	H	I	24	1354	26	21	1357	554	580
Gm18.VB.69	Gm18	42070000	42710000	640000	16	W	B	W	W	B	W	I	23	1763	23	25	1757	56	1326
Gm18.VB.70	Gm18	42710000	42820000	110000	1	W	W	W	W	W	W	W	3	14	1	1	17	2	10
Gm18.VB.71	Gm18	42820000	43760000	940000	17	W	B	W	W	B	W	I	33	3793	30	30	3802	83	2196
Gm18.VB.72	Gm18	43760000	44840000	1080000	6	W	W	W	W	W	W	I	43	100	39	36	98	75	2280
Gm18.VB.73	Gm18	44840000	45510000	670000	14	W	B	W	W	B	W	I	19	2764	22	20	2788	61	1901
Gm18.VB.74	Gm18	45510000	45930000	420000	11	W	B	W	W	B	H	I	38	2796	44	36	2715	773	1562
Gm18.VB.75	Gm18	45930000	46010000	80000	3	W	W	W	W	W	H	I	0	1	1	3	1	646	107

Gm18.VB.76	Gm18	46010000	47420000	1410000	21	W	W	W	W	W	W	I	36	77	36	40	69	112	4512
Gm18.VB.77	Gm18	47420000	48530000	1110000	29	W	W	W	W	W	H	I	64	117	70	56	89	5075	4028
Gm18.VB.78	Gm18	48530000	49390000	860000	36	W	B	W	W	B	B	I	42	5084	34	41	5002	4894	3839
Gm18.VB.79	Gm18	49390000	50890000	1500000	39	W	W	W	W	W	W	I	106	178	98	83	172	168	5968
Gm18.VB.80	Gm18	50890000	52170000	1280000	48	W	B	W	W	B	W	I	58	10102	58	55	9714	49	9613
Gm18.VB.81	Gm18	52170000	52310000	140000	7	W	W	W	W	W	W	I	3	15	3	5	13	3	343
Gm18.VB.82	Gm18	52310000	52600000	290000	11	W	B	W	W	B	W	I	39	714	37	37	675	32	1014
Gm18.VB.83	Gm18	52600000	53540000	940000	48	W	B	W	W	B	H	I	62	8276	68	68	7759	7425	5209
Gm18.VB.84	Gm18	53540000	53820000	280000	20	W	B	W	W	B	W	I	10	1833	11	11	1730	12	1661
Gm18.VB.85	Gm18	53820000	54630000	810000	59	W	W	W	W	W	W	I	29	36	30	29	33	30	3912
Gm18.VB.86	Gm18	54630000	55040000	410000	33	W	W	W	W	W	H	I	4	5	3	3	5	2331	1439
Gm18.VB.87	Gm18	55040000	55160000	120000	9	W	W	W	W	W	W	I	7	12	7	4	3	5	390
Gm18.VB.88	Gm18	55160000	58290000	3130000	273	W	B	W	W	B	H	I	306	18708	305	294	17242	16429	10100
Gm18.VB.89	Gm18	58290000	58860000	570000	47	W	B	B	W	B	B	I	48	3513	3605	49	3236	3212	2119
Gm18.VB.90	Gm18	58860000	58870000	10000	1	W	W	S	W	W	S	I	0	4	5	0	4	5	19
Gm18.VB.91	Gm18	58870000	59050000	180000	18	W	W	W	W	W	W	I	2	2	3	2	3	5	575
Gm18.VB.92	Gm18	59050000	59290000	240000	30	W	W	W	W	W	H	I	0	0	0	0	0	444	420
Gm18.VB.93	Gm18	59290000	59870000	580000	63	W	B	B	B	B	H	I	43	3373	3412	3336	3021	2166	1884
Gm18.VB.94	Gm18	59870000	60080000	210000	24	W	B	B	B	B	W	I	24	639	642	621	568	9	432
Gm18.VB.95	Gm18	60080000	60310000	230000	29	W	B	B	B	B	H	I	6	1248	1273	1248	1155	862	654
Gm18.VB.96	Gm18	60310000	60620000	310000	33	W	W	W	W	W	W	I	11	17	19	18	10	10	946
Gm18.VB.97	Gm18	60620000	60690000	70000	5	W	W	W	W	W	H	I	5	3	5	3	3	53	139
Gm18.VB.98	Gm18	60690000	60920000	230000	29	W	W	W	W	W	W	I	5	5	4	5	2	12	835
Gm18.VB.99	Gm18	60920000	62300000	1380000	144	W	W	W	W	W	H	I	42	45	48	45	36	1028	3111
Gm18.VB.100	Gm18	62300000	62308140	8140	0	W	B	W	G	B	H	I	9	2	8	5	2	17	21
Gm19.VB.1	Gm19	0	170000	170000	13	W	W	S	W	W	H	I	21	21	705	21	8	748	514
Gm19.VB.2	Gm19	170000	280000	110000	9	W	W	W	W	W	W	I	9	14	22	12	12	17	261
Gm19.VB.3	Gm19	280000	690000	410000	43	W	W	S	W	W	H	I	60	85	1558	82	59	699	1121
Gm19.VB.4	Gm19	690000	900000	210000	21	W	W	S	W	S	H	I	20	20	1209	21	1079	395	649
Gm19.VB.5	Gm19	900000	1000000	100000	8	W	W	S	W	S	W	I	0	0	524	0	457	2	126
Gm19.VB.6	Gm19	1000000	1110000	110000	9	W	W	W	W	W	W	I	16	14	19	14	4	7	440
Gm19.VB.7	Gm19	1110000	1160000	50000	4	W	W	S	W	S	W	I	0	4	170	4	161	4	108
Gm19.VB.8	Gm19	1160000	1230000	70000	7	W	W	S	W	S	H	I	17	19	492	19	443	371	251
Gm19.VB.9	Gm19	1230000	1430000	200000	16	W	W	W	W	W	H	I	29	39	37	44	25	647	653
Gm19.VB.10	Gm19	1430000	1530000	100000	5	W	W	W	W	W	W	I	12	12	9	10	5	11	261
Gm19.VB.11	Gm19	1530000	1540000	10000	0	W	W	W	W	W	H	I	0	0	0	0	0	30	27
Gm19.VB.12	Gm19	1540000	1750000	210000	10	W	W	W	W	W	W	I	24	27	33	26	17	21	503
Gm19.VB.13	Gm19	1750000	1780000	30000	1	W	W	S	W	S	W	I	2	2	64	2	62	2	102
Gm19.VB.14	Gm19	1780000	1790000	10000	1	W	W	W	W	W	W	I	0	0	0	0	0	0	35
Gm19.VB.15	Gm19	1790000	1820000	30000	4	W	B	W	B	W	W	I	0	65	1	64	1	3	53
Gm19.VB.16	Gm19	1820000	1930000	110000	5	W	W	W	W	W	W	I	0	13	7	13	5	10	303
Gm19.VB.17	Gm19	1930000	2170000	240000	15	W	B	W	B	W	B	I	30	1052	42	1032	25	1006	592
Gm19.VB.18	Gm19	2170000	2210000	40000	4	W	W	S	W	W	W	I	2	1	77	1	4	2	126
Gm19.VB.19	Gm19	2210000	2450000	240000	12	W	B	S	B	D	B	I	43	613	1118	584	276	580	585
Gm19.VB.20	Gm19	2450000	2650000	200000	7	W	B	S	B	S	W	I	38	521	377	516	339	34	737
Gm19.VB.21	Gm19	2650000	2850000	200000	8	W	W	W	W	W	W	I	8	29	31	25	21	7	1137
Gm19.VB.22	Gm19	2850000	3300000	450000	19	W	B	B	B	B	W	I	16	1729	1743	1692	1725	17	1655
Gm19.VB.23	Gm19	3300000	3440000	140000	8	W	W	W	W	W	W	I	27	35	36	36	33	30	718
Gm19.VB.24	Gm19	3440000	3490000	50000	5	W	B	B	B	B	W	I	0	205	204	200	195	0	274
Gm19.VB.25	Gm19	3490000	6790000	3300000	127	W	W	W	W	W	W	I	202	272	315	261	288	271	11243
Gm19.VB.26	Gm19	6790000	7530000	740000	19	W	B	B	B	B	W	I	32	2170	2204	2139	2154	51	2294
Gm19.VB.27	Gm19	7530000	7580000	50000	1	W	W	W	W	W	W	I	1	19	18	17	15	1	97
Gm19.VB.28	Gm19	7580000	7690000	110000	1	W	W	W	W	W	W	W	5	18	17	24	20	3	43
Gm19.VB.29	Gm19	7690000	7730000	40000	1	W	W	W	W	W	W	I	0	5	6	9	9	1	42
Gm19.VB.30	Gm19	7730000	8550000	820000	34	W	B	S	B	S	W	I	89	2649	4127	2595	3910	97	3233
Gm19.VB.31	Gm19	8550000	8900000	350000	13	W	B	S	B	S	B	I	13	1339	1585	1330	1507	1270	1000
Gm19.VB.32	Gm19	8900000	14520000	5620000	59	W	W	S	W	S	H	I	333	388	20396	358	20484	19973	15444
Gm19.VB.33	Gm19	14520000	14530000	10000	0	W	W	W	W	D	W	I	0	1	2	1	8	0	43
Gm19.VB.34	Gm19	14530000	14820000	290000	1	W	W	W	W	W	W	I	11	15	26	14	23	17	623
Gm19.VB.35	Gm19	14820000	14910000	90000	0	W	W	W	W	W	W	I	1	2	7	2	3	10	176
Gm19.VB.36	Gm19	14910000	27440000	12530000	89	W	W	S	W	S	S	I	521	707	35329	683	35679	36072	27538
Gm19.VB.37	Gm19	27440000	27450000	10000	0	W	B	S	B	D	D	I	11	16	84	17	56	60	17
Gm19.VB.38	Gm19	27450000	29880000	2430000	47	W	B	W	B	W	W	I	118	14668	180	14518	176	244	8506
Gm19.VB.39	Gm19	29880000	33080000	3200000	74	W	B	B	B	B	W	I	146	16433	16333	16283	15919	319	10119
Gm19.VB.40	Gm19	33080000	33840000	760000	14	W	W	S	W	S	W	I	62	109	3337	106	3221	82	2349
Gm19.VB.41	Gm19	33840000	33860000	20000	1	W	B	S	B	S	W	I	2	139	85	136	85	2	67
Gm19.VB.42	Gm19	33860000	34290000	430000	14	W	B	W	B	W	W	I	23	1887	31	1861	32	40	1184
Gm19.VB.43	Gm19	34290000	34570000	280000	12	W	W	W	W	W	W	I	21	32	17	37	18	35	1163
Gm19.VB.44	Gm19	34570000	34610000	40000	2	W	B	W	B	W	W	I	2	119	2	120	2	3	115
Gm19.VB.45	Gm19	34610000	34800000	190000	8	W	W	W	W	W	W	I	6	17	7	17	5	6	533
Gm19.VB.46	Gm19	34800000	35560000	760000	35	W	B	W	B	W	W	I	43	3134	42	3147	38	61	2317
Gm19.VB.47	Gm19	35560000	36090000	530000	22	W	B	W	B	W	H	I	41	2397	39	2379	27	2767	2340
Gm19.VB.48	Gm19	36090000	36220000	130000	3	W	W	W	W	W	H	I	1	13	1	13	1	570	396
Gm19.VB.49	Gm19	36220000	36280000	60000	5	W	B	W	B	W	H	I	0	606	0	600	0	275	139
Gm19.VB.50	Gm19	36280000	36390000	110000	5	W	W	W	W	W	H	I	10	21	11	19	8	802	567
Gm19.VB.51	Gm19	36390000	36420000	30000	5	W	W	W	W	W	W	I	0	3	0	3	0	3	88
Gm19.VB.52	Gm19	36420000	36540000	120000	6	W	B	W	B	W	W	I	82	385	77	380	49	57	605
Gm19.VB.53	Gm19	36540000	36910000	370000	24	W	B	W	B	W	H	I	21	1157	21	1167	14	1390	1558

Gm19.VB.54	Gm19	36910000	37150000	240000	14	W	W	W	W	W	W	I	21	29	19	27	5	14	702
Gm19.VB.55	Gm19	37150000	37160000	10000	0	W	B	W	B	W	W	I	0	69	0	69	0	0	18
Gm19.VB.56	Gm19	37160000	37400000	240000	11	W	W	W	W	W	W	I	35	45	31	44	22	21	976
Gm19.VB.57	Gm19	37400000	37410000	10000	0	W	B	W	B	W	W	I	0	14	0	13	0	1	39
Gm19.VB.58	Gm19	37410000	37590000	180000	14	W	B	W	B	W	H	I	5	284	3	277	0	149	594
Gm19.VB.59	Gm19	37590000	37810000	220000	16	W	W	W	W	W	H	I	7	29	6	34	6	602	669
Gm19.VB.60	Gm19	37810000	38220000	410000	37	W	B	W	B	W	H	I	18	1063	21	1027	20	1175	1198
Gm19.VB.61	Gm19	38220000	38260000	40000	2	W	W	W	W	W	H	I	0	2	0	2	0	124	82
Gm19.VB.62	Gm19	38260000	38400000	140000	10	W	W	W	W	W	W	I	13	18	15	15	9	14	472
Gm19.VB.63	Gm19	38400000	38440000	40000	1	W	W	W	W	W	H	I	0	0	0	0	0	179	62
Gm19.VB.64	Gm19	38440000	38480000	40000	1	W	B	W	B	W	H	I	5	193	5	184	5	188	87
Gm19.VB.65	Gm19	38480000	38940000	460000	28	W	W	W	W	W	H	I	36	54	39	56	33	942	1217
Gm19.VB.66	Gm19	38940000	39080000	140000	11	W	W	W	W	W	W	W	12	20	12	20	5	18	13
Gm19.VB.67	Gm19	39080000	39100000	20000	1	W	W	W	W	W	H	W	0	3	0	3	0	64	0
Gm19.VB.68	Gm19	39100000	39290000	190000	14	W	B	W	B	W	B	I	6	842	6	829	5	753	555
Gm19.VB.69	Gm19	39290000	39610000	320000	22	W	B	W	B	W	W	I	21	1411	15	1339	16	35	1103
Gm19.VB.70	Gm19	39610000	40190000	580000	44	W	B	W	B	W	H	I	38	2397	43	2330	34	2332	1230
Gm19.VB.71	Gm19	40190000	40790000	600000	60	W	B	S	B	S	S	I	36	2037	2554	2001	2272	2344	1617
Gm19.VB.72	Gm19	40790000	40930000	140000	9	W	W	W	W	W	W	I	2	9	11	9	10	11	216
Gm19.VB.73	Gm19	40930000	41030000	100000	8	W	W	S	W	S	S	I	32	37	469	37	401	399	211
Gm19.VB.74	Gm19	41030000	41080000	50000	6	W	B	B	B	B	B	I	0	243	229	239	204	209	160
Gm19.VB.75	Gm19	41080000	41320000	240000	25	W	W	S	W	D	S	I	4	14	1126	14	208	1022	458
Gm19.VB.76	Gm19	41320000	41340000	20000	1	W	B	S	B	B	S	I	1	42	63	40	36	62	37
Gm19.VB.77	Gm19	41340000	41350000	10000	3	W	B	S	B	W	S	I	0	12	46	12	4	45	32
Gm19.VB.78	Gm19	41350000	41380000	30000	3	W	B	W	B	W	W	W	1	46	1	46	1	1	13
Gm19.VB.79	Gm19	41380000	41550000	170000	10	W	W	W	W	W	W	I	13	25	24	24	14	21	310
Gm19.VB.80	Gm19	41550000	41630000	80000	7	W	W	S	W	W	H	I	16	17	121	18	10	98	82
Gm19.VB.81	Gm19	41630000	41810000	180000	14	W	B	S	B	S	S	I	21	356	336	358	271	320	309
Gm19.VB.82	Gm19	41810000	41940000	130000	12	W	B	S	B	W	S	I	15	192	255	179	18	234	260
Gm19.VB.83	Gm19	41940000	42200000	260000	24	W	B	S	G	D	S	I	12	782	428	657	238	367	750
Gm19.VB.84	Gm19	42200000	42350000	150000	11	W	B	S	S	W	S	I	25	93	337	329	20	301	306
Gm19.VB.85	Gm19	42350000	42470000	120000	10	W	B	S	S	D	S	I	8	566	125	126	84	116	315
Gm19.VB.86	Gm19	42470000	42700000	230000	24	W	B	W	W	W	W	I	7	304	18	17	9	18	500
Gm19.VB.87	Gm19	42700000	42830000	130000	12	W	B	S	S	D	W	I	0	204	464	454	123	3	360
Gm19.VB.88	Gm19	42830000	42850000	20000	1	W	B	S	S	W	W	I	1	21	45	45	3	0	51
Gm19.VB.89	Gm19	42850000	42960000	110000	9	W	W	S	S	W	W	I	0	4	420	423	4	2	343
Gm19.VB.90	Gm19	42960000	43130000	170000	14	W	B	B	B	B	W	I	11	441	421	399	339	6	318
Gm19.VB.91	Gm19	43130000	43350000	220000	26	W	W	W	W	W	W	I	20	30	30	30	14	13	470
Gm19.VB.92	Gm19	43350000	43890000	540000	57	W	B	W	W	W	W	I	10	1349	43	40	33	26	975
Gm19.VB.93	Gm19	43890000	44040000	150000	13	W	W	W	W	W	W	I	0	3	2	2	1	2	199
Gm19.VB.94	Gm19	44040000	44270000	230000	20	W	W	S	S	W	W	I	6	20	1230	1186	16	23	512
Gm19.VB.95	Gm19	44270000	44280000	10000	0	W	B	S	S	W	H	W	2	8	13	12	0	18	3
Gm19.VB.96	Gm19	44280000	44580000	300000	35	W	B	W	W	W	H	I	0	1221	9	9	5	1240	738
Gm19.VB.97	Gm19	44580000	44960000	380000	35	W	B	S	S	S	H	I	4	765	854	830	808	854	777
Gm19.VB.98	Gm19	44960000	45800000	840000	90	W	B	W	W	W	B	I	29	3169	54	56	54	2997	2074
Gm19.VB.99	Gm19	45800000	46100000	300000	34	W	B	S	S	S	B	I	23	818	881	869	795	843	528
Gm19.VB.100	Gm19	46100000	46270000	170000	21	W	W	W	W	W	H	I	3	14	5	5	5	306	278
Gm19.VB.101	Gm19	46270000	46300000	30000	5	W	B	W	W	W	H	I	19	117	17	7	4	117	95
Gm19.VB.102	Gm19	46300000	46390000	90000	14	W	W	W	W	W	H	I	0	6	1	2	1	44	141
Gm19.VB.103	Gm19	46390000	46810000	420000	50	W	W	W	W	W	W	I	36	57	53	46	38	47	824
Gm19.VB.104	Gm19	46810000	46960000	150000	17	W	B	W	W	W	W	I	13	146	11	15	9	14	216
Gm19.VB.105	Gm19	46960000	46970000	10000	1	W	W	W	W	W	W	I	0	0	0	0	0	0	14
Gm19.VB.106	Gm19	46970000	47020000	50000	7	W	W	S	S	S	W	I	3	6	61	62	59	4	95
Gm19.VB.107	Gm19	47020000	47130000	110000	13	W	W	S	S	S	S	I	1	5	339	339	327	311	292
Gm19.VB.108	Gm19	47130000	47230000	100000	13	W	W	S	S	D	W	I	4	8	108	104	82	4	165
Gm19.VB.109	Gm19	47230000	47580000	350000	30	W	W	S	S	S	S	I	16	28	1288	1240	1149	1151	686
Gm19.VB.110	Gm19	47580000	48340000	760000	89	W	B	B	B	B	H	I	34	3145	3247	3136	2923	3066	1596
Gm19.VB.111	Gm19	48340000	48590000	250000	31	W	W	W	W	W	W	I	15	25	24	22	22	19	605
Gm19.VB.112	Gm19	48590000	48890000	300000	38	W	W	W	W	W	H	I	4	14	14	14	12	479	814
Gm19.VB.113	Gm19	48890000	49010000	120000	16	W	W	W	W	W	W	I	0	11	12	12	11	14	291
Gm19.VB.114	Gm19	49010000	49810000	800000	91	W	B	B	B	B	H	I	55	1373	1390	1345	1257	1462	1673
Gm19.VB.115	Gm19	49810000	50280000	470000	59	W	W	W	W	W	W	I	13	33	33	33	33	37	716
Gm19.VB.116	Gm19	50280000	50450000	170000	16	W	B	B	B	B	B	I	1	173	170	175	152	159	265
Gm19.VB.117	Gm19	50450000	50580000	130000	10	W	W	W	W	W	W	I	2	24	25	23	19	20	305
Gm19.VB.118	Gm19	50580000	50589441	9441	1	W	W	W	W	W	W	I	0	1	1	1	0	0	11
Gm20.VB.1	Gm20	0	60000	60000	5	W	B	W	W	B	B	I	10	58	9	10	56	56	44
Gm20.VB.2	Gm20	60000	170000	110000	8	W	W	W	W	W	W	I	9	21	9	9	21	21	208
Gm20.VB.3	Gm20	170000	360000	190000	18	W	B	W	W	B	B	I	5	662	6	8	617	622	444
Gm20.VB.4	Gm20	360000	480000	120000	8	W	W	W	W	W	W	I	0	9	1	0	6	10	196
Gm20.VB.5	Gm20	480000	530000	50000	5	W	B	W	W	D	H	I	1	222	0	3	111	174	153
Gm20.VB.6	Gm20	530000	540000	10000	2	W	B	W	W	W	B	I	0	17	0	0	1	17	9
Gm20.VB.7	Gm20	540000	640000	100000	8	W	W	W	W	W	W	I	5	10	5	3	3	9	195
Gm20.VB.8	Gm20	640000	690000	50000	8	W	W	W	W	W	H	I	0	6	4	4	4	104	117
Gm20.VB.9	Gm20	690000	730000	40000	4	W	B	B	B	B	B	I	0	135	134	133	130	130	71
Gm20.VB.10	Gm20	730000	740000	10000	0	W	B	B	B	B	W	I	0	8	8	8	9	4	15
Gm20.VB.11	Gm20	740000	840000	100000	13	W	W	S	S	W	W	I	0	5	434	427	3	3	289
Gm20.VB.12	Gm20	840000	1740000	900000	65	W	B	S	B	D	H	I	481	2899	2426	2912	1171	1597	2179
Gm20.VB.13	Gm20	1740000	2040000	300000	15	W	B	S	B	D	W	I	15	957	1319	951	468	18	846

Gm20.VB.14	Gm20	2040000	2200000	160000	16	W	W	S	W	W	H	I	3	56	248	54	20	207	414
Gm20.VB.15	Gm20	2200000	2470000	270000	14	W	B	B	B	B	B	I	19	1305	1303	1299	1252	1257	696
Gm20.VB.16	Gm20	2470000	2650000	180000	5	W	W	W	W	W	W	I	9	23	17	20	11	11	452
Gm20.VB.17	Gm20	2650000	2990000	340000	22	W	B	W	B	W	B	I	8	953	19	937	86	918	636
Gm20.VB.18	Gm20	2990000	3010000	20000	1	W	W	S	W	W	W	I	0	0	129	0	0	0	79
Gm20.VB.19	Gm20	3010000	3080000	70000	2	W	B	S	B	D	B	I	6	733	374	719	231	723	334
Gm20.VB.20	Gm20	3080000	3100000	20000	1	W	W	S	W	W	W	I	0	0	49	0	0	0	54
Gm20.VB.21	Gm20	3100000	3140000	40000	1	W	B	W	B	W	B	I	0	42	0	42	5	42	172
Gm20.VB.22	Gm20	3140000	11250000	8110000	121	W	W	W	W	W	W	I	415	791	674	739	493	779	18193
Gm20.VB.23	Gm20	11250000	11310000	60000	1	W	W	W	G	W	W	I	0	24	6	25	6	20	50
Gm20.VB.24	Gm20	11310000	14100000	2790000	38	W	W	W	W	W	W	I	196	389	257	366	234	378	6322
Gm20.VB.25	Gm20	14100000	15530000	1430000	16	W	B	W	B	W	B	I	41	7680	134	7589	357	7871	6164
Gm20.VB.26	Gm20	15530000	15640000	110000	3	W	B	W	B	D	B	I	7	738	12	724	55	715	414
Gm20.VB.27	Gm20	15640000	15800000	160000	4	W	B	W	B	W	B	I	1	583	10	571	33	543	388
Gm20.VB.28	Gm20	15800000	17340000	1540000	25	W	W	W	W	W	W	I	54	106	95	107	71	106	3305
Gm20.VB.29	Gm20	17340000	17820000	480000	3	W	B	W	B	W	B	I	16	1818	42	1786	94	1833	1459
Gm20.VB.30	Gm20	17820000	17980000	160000	0	W	W	W	W	W	W	I	18	17	18	15	7	20	178
Gm20.VB.31	Gm20	17980000	18050000	70000	0	W	W	W	W	W	W	I	0	17	9	7	5	3	190
Gm20.VB.32	Gm20	18050000	18060000	10000	0	W	W	W	G	W	W	I	0	0	1	8	1	4	40
Gm20.VB.33	Gm20	18060000	18390000	330000	3	W	B	W	B	W	B	I	36	1146	56	1091	80	1115	1194
Gm20.VB.34	Gm20	18390000	22460000	4070000	30	W	W	W	W	W	W	I	141	356	262	333	196	367	5125
Gm20.VB.35	Gm20	22460000	22470000	10000	0	W	B	W	G	W	W	I	0	10	4	5	3	3	24
Gm20.VB.36	Gm20	22470000	23170000	700000	8	W	B	W	B	W	B	I	17	373	63	371	74	372	1768
Gm20.VB.37	Gm20	23170000	23470000	300000	5	W	W	W	W	W	W	I	2	17	21	19	18	20	1143
Gm20.VB.38	Gm20	23470000	23590000	120000	1	W	B	B	B	B	B	I	0	386	377	382	378	392	69
Gm20.VB.39	Gm20	23590000	23700000	110000	4	W	W	W	W	W	W	I	5	31	33	27	26	31	59
Gm20.VB.40	Gm20	23700000	23750000	50000	1	W	B	B	B	B	B	I	1	41	43	44	41	45	385
Gm20.VB.41	Gm20	23750000	24350000	600000	11	W	W	W	W	W	W	I	16	62	64	59	47	55	367
Gm20.VB.42	Gm20	24350000	26810000	2460000	30	W	B	B	B	B	B	I	124	11829	11989	11729	11948	12035	9558
Gm20.VB.43	Gm20	26810000	30180000	3370000	46	W	W	W	W	W	W	I	151	359	355	334	260	352	13276
Gm20.VB.44	Gm20	30180000	30510000	330000	7	W	B	B	B	B	B	I	1	1433	1445	1416	1407	1439	1180
Gm20.VB.45	Gm20	30510000	32580000	2070000	67	W	W	W	W	W	W	I	123	243	128	127	218	231	5196
Gm20.VB.46	Gm20	32580000	32710000	130000	13	W	B	W	W	W	B	I	9	599	9	9	9	575	463
Gm20.VB.47	Gm20	32710000	33040000	330000	22	W	W	W	W	W	W	I	12	27	13	11	10	24	1277
Gm20.VB.48	Gm20	33040000	33070000	30000	6	W	B	W	W	W	W	I	0	65	0	0	0	3	112
Gm20.VB.49	Gm20	33070000	34200000	1130000	104	W	B	W	W	W	H	I	41	4619	46	41	36	5355	3695
Gm20.VB.50	Gm20	34200000	34370000	170000	16	W	B	S	S	S	H	I	19	827	844	807	737	697	476
Gm20.VB.51	Gm20	34370000	34530000	160000	15	W	B	B	B	B	W	I	10	780	782	768	716	14	421
Gm20.VB.52	Gm20	34530000	34580000	50000	3	W	B	W	W	W	H	I	1	193	1	1	0	91	156
Gm20.VB.53	Gm20	34580000	34630000	50000	4	W	W	W	W	W	H	I	10	13	12	13	13	61	79
Gm20.VB.54	Gm20	34630000	34760000	130000	7	W	W	W	W	W	W	I	7	26	23	17	12	13	555
Gm20.VB.55	Gm20	34760000	34830000	70000	5	W	W	W	W	W	H	I	14	14	11	11	15	99	171
Gm20.VB.56	Gm20	34830000	35050000	220000	17	W	B	W	W	W	H	I	41	977	40	38	39	435	853
Gm20.VB.57	Gm20	35050000	35240000	190000	14	W	W	W	W	W	H	I	37	40	37	35	28	128	934
Gm20.VB.58	Gm20	35240000	35330000	90000	10	W	B	W	W	W	H	I	4	520	4	4	4	225	205
Gm20.VB.59	Gm20	35330000	35340000	10000	2	W	B	S	W	S	W	W	0	101	6	4	6	1	4
Gm20.VB.60	Gm20	35340000	35680000	340000	31	W	B	B	B	B	H	I	29	1699	1708	1647	1460	997	1054
Gm20.VB.61	Gm20	35680000	35940000	260000	24	W	B	W	W	W	W	I	19	1183	30	25	20	18	920
Gm20.VB.62	Gm20	35940000	36170000	230000	31	W	B	W	W	W	H	I	3	632	9	8	8	456	659
Gm20.VB.63	Gm20	36170000	36270000	100000	12	W	W	W	W	W	W	I	2	4	2	2	2	5	274
Gm20.VB.64	Gm20	36270000	36290000	20000	3	W	B	W	W	W	B	I	0	67	0	0	0	65	43
Gm20.VB.65	Gm20	36290000	36400000	110000	8	W	W	W	W	W	W	I	2	12	5	3	3	10	460
Gm20.VB.66	Gm20	36400000	36470000	70000	6	W	W	W	W	W	W	I	1	6	1	1	1	26	363
Gm20.VB.67	Gm20	36470000	36600000	130000	14	W	B	W	W	W	H	I	13	415	14	13	7	377	461
Gm20.VB.68	Gm20	36600000	36760000	160000	13	W	W	W	W	W	H	I	27	28	29	24	32	110	671
Gm20.VB.69	Gm20	36760000	36900000	140000	19	W	W	W	W	W	W	I	13	18	11	12	11	15	485
Gm20.VB.70	Gm20	36900000	36950000	50000	6	W	B	W	W	W	W	I	1	126	1	1	1	3	97
Gm20.VB.71	Gm20	36950000	37120000	170000	16	W	W	W	W	W	W	I	10	20	23	17	11	10	380
Gm20.VB.72	Gm20	37120000	37150000	30000	3	W	B	W	W	W	W	I	0	61	1	1	1	0	62
Gm20.VB.73	Gm20	37150000	37650000	500000	39	W	W	W	W	W	W	I	47	58	66	65	46	144	1287
Gm20.VB.74	Gm20	37650000	37670000	20000	1	W	W	W	W	W	H	I	0	1	1	1	1	31	96
Gm20.VB.75	Gm20	37670000	37890000	220000	21	W	W	W	W	W	W	I	13	21	22	23	19	22	566
Gm20.VB.76	Gm20	37890000	37990000	100000	6	W	W	S	S	S	W	I	15	12	542	541	462	12	263
Gm20.VB.77	Gm20	37990000	38050000	60000	7	W	B	S	S	S	W	I	9	251	328	297	271	10	206
Gm20.VB.78	Gm20	38050000	38300000	250000	22	W	B	W	W	W	W	I	27	1603	33	29	27	44	452
Gm20.VB.79	Gm20	38300000	38430000	130000	12	W	B	S	S	S	W	I	10	606	603	555	493	8	231
Gm20.VB.80	Gm20	38430000	38630000	200000	23	W	B	W	W	W	W	I	6	709	5	7	4	12	318
Gm20.VB.81	Gm20	38630000	38700000	70000	5	W	B	W	W	W	H	I	6	258	7	6	5	71	88
Gm20.VB.82	Gm20	38700000	38840000	140000	18	W	B	W	W	W	W	I	12	435	14	13	5	11	200
Gm20.VB.83	Gm20	38840000	39370000	530000	47	W	W	W	W	W	W	I	50	77	51	51	34	51	1008
Gm20.VB.84	Gm20	39370000	39420000	50000	2	W	B	W	W	W	W	I	0	164	0	0	0	2	145
Gm20.VB.85	Gm20	39420000	39710000	290000	28	W	B	W	W	W	B	I	29	1103	28	28	24	1015	522
Gm20.VB.86	Gm20	39710000	39910000	200000	20	W	W	W	W	W	W	I	29	29	33	31	34	32	382
Gm20.VB.87	Gm20	39910000	39930000	20000	0	W	B	W	B	W	B	I	6	71	6	71	2	66	18
Gm20.VB.88	Gm20	39930000	40080000	150000	13	W	W	W	W	W	W	I	10	21	10	23	10	21	286
Gm20.VB.89	Gm20	40080000	40490000	410000	39	W	B	W	B	W	H	I	24	1139	24	1106	20	1184	983
Gm20.VB.90	Gm20	40490000	40690000	200000	13	W	W	W	W	W	H	I	18	18	18	17	16	284	364
Gm20.VB.91	Gm20	40690000	41270000	580000	39	W	W	W	W	W	W	I	62	60	66	63	59	73	1078

Gm20.VB.92	Gm20	41270000	41520000	250000	22	W	W	W	W	W	H	I	21	22	21	19	12	492	513
Gm20.VB.93	Gm20	41520000	41630000	110000	8	W	W	W	W	W	W	W	0	0	1	0	1	6	40
Gm20.VB.94	Gm20	41630000	42080000	450000	34	W	W	W	W	W	H	I	39	39	45	48	34	764	1218
Gm20.VB.95	Gm20	42080000	42930000	850000	91	W	W	W	W	W	W	I	28	43	28	42	20	38	1808
Gm20.VB.96	Gm20	42930000	42940000	10000	0	W	W	W	W	W	H	I	1	5	1	5	1	6	19
Gm20.VB.97	Gm20	42940000	43230000	290000	31	W	B	W	B	W	B	I	35	814	40	803	31	734	512
Gm20.VB.98	Gm20	43230000	43430000	200000	21	W	W	W	W	W	W	I	17	25	18	24	17	26	260
Gm20.VB.99	Gm20	43430000	43480000	50000	5	W	B	W	B	W	W	I	19	285	21	258	18	25	115
Gm20.VB.100	Gm20	43480000	43590000	110000	9	W	B	W	B	B	W	I	34	555	38	540	526	33	297
Gm20.VB.101	Gm20	43590000	44710000	1120000	114	W	B	W	B	B	H	I	46	4404	48	4351	4052	3571	2518
Gm20.VB.102	Gm20	44710000	44850000	140000	16	W	B	W	B	B	W	I	7	447	5	452	419	7	395
Gm20.VB.103	Gm20	44850000	44870000	20000	2	W	B	W	B	B	B	I	0	147	0	138	131	142	109
Gm20.VB.104	Gm20	44870000	45360000	490000	40	W	W	W	W	W	W	I	13	27	13	34	24	14	905
Gm20.VB.105	Gm20	45360000	45450000	90000	10	W	B	W	B	B	W	I	3	153	3	151	129	5	193
Gm20.VB.106	Gm20	45450000	45820000	370000	47	W	B	W	B	B	B	I	14	939	13	931	884	858	699
Gm20.VB.107	Gm20	45820000	45970000	150000	15	W	B	W	B	B	W	I	5	586	5	583	564	9	348
Gm20.VB.108	Gm20	45970000	46210000	240000	29	W	W	W	W	W	W	I	19	22	20	23	18	15	365
Gm20.VB.109	Gm20	46210000	46540000	330000	33	W	B	W	B	B	W	I	15	739	15	737	675	16	637
Gm20.VB.110	Gm20	46540000	46770000	230000	24	W	W	W	W	W	W	I	20	29	23	26	24	23	294
Gm20.VB.111	Gm20	46770000	46773167	3167	0	W	W	W	W	W	W	I	0	0	0	0	0	0	3
