# Supplementary material for: Variation block-based genomics method for crop plants
Source: BMC Genomics. 2014 Jun 15;15:477. doi: 10.1186/1471-2164-15-477 (PMC4229737; doi:10.1186/1471-2164-15-477)
Supplement: Additional file 13: Table S4 — Hilum colors and the HD8.386 indel marker analysis results for the 86 soybean cultivars. [file 1471-2164-15-477-S13.pdf]

Table S4 - Hilum colors and HD8.386 indel marker analysis results of 86 soybean cultivars.
The genotype of each *I* and putative *HC* loci were determined by indel marker analysis.

No.	Cultivar	Hilum color	HD8.386	Genotype	No.	Cultivar	Hilum color	HD8.386	Genotype
1	HwangKeum (*I*)	Yellow	-	$i^i + I^h$	44	JangYeop	Yellow	-	$i^i + I^h$
2	Williams 82 (i^i)	Black	Deletion	$i^i + i^h$	45	JinMi	Yellow	-	$i^i + I^h$
3	Enrei (*I*)	Yellow	-	$i^i + I^h$	46	JinPum	Yellow	-	$i^i + I^h$
4	AlChan	Brown	Deletion	$i^i + i^h$	47	JinPum 2	Yellow	-	$i^i + I^h$
5	AnPyeong	Yellow	-	$i^i + I^h$	48	JoNam	Yellow	-	$i^i + I^h$
6	BaekCheon	Imperfect black	Deletion	$i^i + i^h$	49	KeunOl	Yellow	-	$i^i + I^h$
7	BaekUn	Brown	Deletion	$i^i + i^h$	50	ManRi	Yellow	-	$i^i + I^h$
8	BangSa	Brown	Deletion	$i^i + i^h$	51	ManSu	Yellow	-	$i^i + I^h$
9	BoGwang	Yellow	-	$i^i + I^h$	52	MilYang	Imperfect black	Deletion	$i^i + i^h$
10	BoSeok	Brown	Deletion	$i^i + i^h$	53	MuHan	Imperfect black	Deletion	$i^i + i^h$
11	BuGwang	Brown	Deletion	$i^i + i^h$	54	MyeongJu	Imperfect black	Deletion	$i^i + i^h$
12	ChengDu 1	Black	Deletion	$i^i + i^h$	55	NamHae	Imperfect black	Deletion	$i^i + i^h$
13	DaChae	Imperfect black	Deletion	$i^i + i^h$	56	NamPoong	Buff	Deletion	$i^i + i^h$
14	DaeHwang	Yellow	-	$i^i + I^h$	57	PalDal	Black	Deletion	$i^i + i^h$
15	DaeMang	Imperfect black	Deletion	$i^i + i^h$	58	PalDo	Imperfect black	Deletion	$i^i + i^h$
16	DaeMang 2	Imperfect black	Deletion	$i^i + i^h$	59	PoongSan	Yellow	-	$i^i + I^h$
17	DaePoong	Brown	Deletion	$i^i + i^h$	60	PoongWon	Buff	Deletion	$i^i + i^h$
18	DaeWon	Yellow	-	$i^i + I^h$	61	SamNam	Buff	Deletion	$i^i + i^h$
19	DaeYang	Yellow	-	$i^i + I^h$	62	SangWon	Yellow	-	$i^i + I^h$
20	DaGi	Brown	Deletion	$i^i + i^h$	63	SeaByeol	Brown	Deletion	$i^i + i^h$
21	DaJang	Imperfect black	Deletion	$i^i + i^h$	64	SeaOl	Buff	Deletion	$i^i + i^h$
22	DaJin	Yellow	-	$i^i + I^h$	65	SeokYang	Brown	Deletion	$i^i + i^h$
23	DanBack	Brown	Deletion	$i^i + i^h$	66	SeoNam	Imperfect black	Deletion	$i^i + i^h$
24	DanMi	Yellow	-	$i^i + I^h$	67	SeonYu	Yellow	-	$i^i + I^h$
25	DanWon	Imperfect black	Deletion	$i^i + i^h$	68	SinGang	Brown	Deletion	$i^i + i^h$
26	DeokKyu	Imperfect black	Deletion	$i^i + i^h$	69	SinGi	Buff	Deletion	$i^i + i^h$
27	DoReMi	Imperfect black	Deletion	$i^i + i^h$	70	SinHwa	Buff	Deletion	$i^i + i^h$
28	DuYu	Black	Deletion	$i^i + i^h$	71	SinPalDal	Imperfect black	Deletion	$i^i + i^h$
29	EunHa	Imperfect black	Deletion	$i^i + i^h$	72	SinPalDal 2	Black	Deletion	$i^i + i^h$
30	GeumGang	Imperfect black	Deletion	$i^i + i^h$	73	SinRok	Yellow	-	$i^i + I^h$
31	GwangAn	Imperfect black	Deletion	$i^i + i^h$	74	SoBack	Yellow	-	$i^i + I^h$
32	GwangGyo	Imperfect black	Deletion	$i^i + i^h$	75	SoDam	Yellow	-	$i^i + I^h$
33	HanNam	Yellow	-	$i^i + I^h$	76	SoGang	Buff	Deletion	$i^i + i^h$
34	HoJang	Yellow	-	$i^i + I^h$	77	SoHo	Brown	Deletion	$i^i + i^h$
35	HoSeo	Brown	Deletion	$i^i + i^h$	78	SoHwang	Yellow	-	$i^i + I^h$
36	HwaEom	Yellow	-	$i^i + I^h$	79	SoJin	Buff	Deletion	$i^i + i^h$
37	HwaSeong	Buff	Deletion	$i^i + i^h$	80	SoMyeong	Buff	Deletion	$i^i + i^h$
38	IkSan	Imperfect black	Deletion	$i^i + i^h$	81	SongHak	Yellow	-	$i^i + I^h$
39	IlMi	Brown	Deletion	$i^i + i^h$	82	SoRok	Black	Deletion	$i^i + i^h$
40	JangGi	Buff	Deletion	$i^i + i^h$	83	SoWon	Buff	Deletion	$i^i + i^h$
41	JangGyeong	Imperfect black	Deletion	$i^i + i^h$	84	TaeGwang	Yellow	-	$i^i + I^h$
42	JangSu	Imperfect black	Deletion	$i^i + i^h$	85	WonGwang	Imperfect black	Deletion	$i^i + i^h$
43	JangWon	Yellow	-	$i^i + I^h$	86	WonHwang	Buff	Deletion	$i^i + i^h$
